# Nonreciprocal complementation of KNOX gene function in land plants

**DOI:** 10.1111/nph.14318

**Published:** 2016-11-25

**Authors:** Eftychios Frangedakis, Denis Saint‐Marcoux, Laura A. Moody, Ester Rabbinowitsch, Jane A. Langdale

**Affiliations:** ^1^ Department of Plant Sciences University of Oxford South Parks Road Oxford OX1 3RB UK

**Keywords:** Arabidopsis, *Ceratopteris richardii*, cross‐species complementation, KNOTTED homeobox genes, land plant evolution, phylogeny, *Physcomitrella patens*, *Selaginella kraussiana*

## Abstract

- Class I KNOTTED‐LIKE HOMEOBOX (KNOX) proteins regulate development of the multicellular diploid sporophyte in both mosses and flowering plants; however, the morphological context in which they function differs.
- In order to determine how Class I KNOX function was modified as land plants evolved, phylogenetic analyses and cross‐species complementation assays were performed.
- Our data reveal that a duplication within the charophyte sister group to land plants led to distinct Class I and Class II KNOX gene families. Subsequently, Class I sequences diverged substantially in the nonvascular bryophyte groups (liverworts, mosses and hornworts), with moss sequences being most similar to those in vascular plants. Despite this similarity, moss mutants were not complemented by vascular plant KNOX genes. Conversely, the Arabidopsis *brevipedicellus* (*bp‐9*) mutant was complemented by the *PpMKN2* gene from the moss *Physcomitrella patens*. Lycophyte KNOX genes also complemented *bp‐9* whereas fern genes only partially complemented the mutant. This lycophyte/fern distinction is mirrored in the phylogeny of KNOX‐interacting BELL proteins, in that a gene duplication occurred after divergence of the two groups.
- Together, our results imply that the moss MKN2 protein can function in a broader developmental context than vascular plant KNOX proteins, the narrower scope having evolved progressively as lycophytes, ferns and flowering plants diverged.

## Introduction

Morphogenesis in all major eukaryotic lineages is regulated at least in part by homeodomain transcription factors (Holland, 2013). Based on phylogeny, two classes of homeodomain transcription factors are assumed to have been present in the eukaryote ancestor – the canonical homeodomain proteins which have a 60 amino acid DNA binding domain and the three amino acid loop extension (TALE) proteins which have a 63 amino acid DNA binding domain (Bharathan *et al*., 1997; Burglin, 1997; Derelle *et al*., 2007). In the green plant lineage, two families of TALE proteins have been distinguished: KNOTTED‐LIKE HOMEOBOX (KNOX) and BELL (Mukherjee *et al*., 2009). KNOX proteins are encoded by a single gene in the unicellular chlorophyte algae that are sister group to the streptophytes (charophyte algae and land plants) (Serikawa & Mandoli, 1999; Lee *et al*., 2008). In the chlorophyte alga *Chlamydomanas reinhardtii*, the KNOX protein GSM accumulates in the cytoplasm of ‘minus’ type gametes, and the BELL protein GSP similarly accumulates in ‘plus’ type gametes. When the two types of gamete fuse, KNOX/BELL dimerization leads to nuclear localization and formation of the diploid zygote (Lee *et al*., 2008). The KNOX protein in the unicellular green alga *Acetabularia acetabulum* similarly becomes nuclear‐localized during the vegetative to reproductive transition (Serikawa & Mandoli, 1999). KNOX/BELL interactions may therefore have evolved in the chlorophytes to regulate the transition from vegetative to reproductive development and/or to specifically facilitate formation of the diploid zygote.

In contrast to the single copy KNOX genes found in chlorophyte algae, several duplication events have led to multiple copies being present in all land plant genomes examined to date (Mukherjee *et al*., 2009). Similarly, BELL gene numbers have expanded from the one or two seen in algal genomes to the many that are present in flowering plants (Mukherjee *et al*., 2009). Members of the KNOX gene family are split into two classes, with representatives of both Class I and Class II genes present in the bryophyte moss *Physcomitrella patens* (Singer & Ashton, 2007; Sakakibara *et al*., 2008), the lycophyte genus *Selaginella* (Harrison *et al*., 2005; Kawai *et al*., 2010), the monilophyte fern *Ceratopteris richardii* (Sano *et al*., 2005) and in all flowering plants examined (Kerstetter *et al*., 1994; Mukherjee *et al*., 2009). This phylogenetic distribution suggests that the gene duplication that led to distinct Class I and Class II KNOX genes occurred in a common ancestor of the mosses and vascular plants.

In *P. patens*, although KNOX genes are expressed in the gametophyte, triple mutants that are null for the three Class I genes *PpMKN2*,* PpMKN4* and *PpMKN5* form normal gametophytes*,* and zygote formation is not disrupted (Sakakibara *et al*., 2008). However, mutants exhibit perturbed cell proliferation in the diploid generation of the life cycle, which in bryophytes is represented by a multicellular sporophyte as opposed to the unicellular zygotes of chlorophyte algae. Although Class I genes promote cell proliferation in the sporophyte, the Class II genes *PpMKN1* and *PpMKN6* promote tissue differentiation, with loss‐of‐function leading to diploid structures having gametophyte bodyplans (Sakakibara *et al*., 2013). Neofunctionalization following the duplication of KNOX genes thus enabled the development of a multicellular diploid sporophyte, which was a bryophyte innovation.

Opposing roles for Class I and Class II KNOX genes also are observed in the flowering plant Arabidopsis, where Class I genes promote cell proliferation in the shoot apical meristem (Long *et al*., 1996; Venglat *et al*., 2002; Belles‐Boix *et al*., 2006) and Class II genes promote tissue differentiation (Furumizu *et al*., 2015). Although the role of Class I genes in promoting cell proliferation in the multicellular sporophyte is conserved in mosses and flowering plants, the context in which the genes operate was modified during the course of land plant evolution. For example, bryophyte sporophytes are determinate, developing as a single unbranched stalk subtending a sporangium (Ligrone *et al*., 2012a), whereas vascular plant sporophytes have highly variable and complex shoot architecture that results from the sustained activity of indeterminate shoot meristems giving rise to determinate lateral organs (leaves) (Sussex & Kerk, 2001). The indeterminate nature of shoot growth in the three vascular plant lineages further varies in terms of the structure of the shoot apex, with lycophyte and monilophyte shoots developing from one or a few apical initials (Steeves & Sussex, 1989; Harrison *et al*., 2007; Jones & Drinnan, 2009; Harrison & Langdale, 2010; Sanders *et al*., 2011; Vasco *et al*., 2013), and seed plant shoots developing from multicellular meristems (Steeves & Sussex, 1989; Barton, 2010). There is currently very little understanding of how Class I KNOX gene function evolved as the developmental mechanisms underpinning shoot growth changed.

In Arabidopsis and other flowering plants with simple leaves, Class I KNOX genes function in the indeterminate shoot meristem but are switched off in determinate leaf primordia (Hay & Tsiantis, 2010). Gene function maintains indeterminacy in the meristem by promoting cytokinin (CK) signalling and inhibiting gibberellic acid (GA) signalling (Sakamoto *et al*., 2001; Hay *et al*., 2002; Jasinski *et al*., 2005). Repression of KNOX activity in the leaf therefore de‐represses GA and suppresses CK, leading to determinate leaf development. A failure to suppress KNOX gene expression in the leaf, through transgenic manipulation or dominant gain of function mutation, results in continued repression of GA, promotion of CK activity, ectopic cellular proliferation, and a resultant lobed or knotted leaf phenotype (Smith *et al*., 1992; Lincoln *et al*., 1994; Sakamoto *et al*., 2001, 2006; Hay *et al*., 2002). Notably, constitutive expression of Class I KNOX genes from both *P. patens* and *C. richardii*, also leads to a lobed leaf phenotype in Arabidopsis (Sano *et al*., 2005; Sakakibara *et al*., 2008). The similar phenotypes seen in lines with transgenes from mosses, ferns and flowering plants indicate that genes from the different lineages have equivalent potential to regulate GA and/or CK targets in the context of the Arabidopsis leaf. However, a true understanding of differences in KNOX gene activity between lineages cannot be obtained from constitutive overexpression studies; instead gene function must be analysed in endogenous expression domains.

In this report we demonstrate that the duplication leading to Class I and Class II KNOX genes occurred in the charophyte algae, before the evolution of a multicellular diploid sporophyte. Representatives of both classes are retained in all land plant lineages except hornworts, where Class I genes could not be identified in the species examined. Although Class I genes from the moss *P. patens* share sequence similarity with those from vascular plants, lycophyte, fern and flowering plant genes all failed to complement loss‐of‐function moss mutants. By contrast, *MKN2* from *P. patens* fully complemented loss‐of‐function *brevipedicellus* (*bp*) mutants in Arabidopsis. *Selaginella kraussiana* Class I genes also complemented *bp* mutants, whereas genes from *C. richardii* only partially complemented them. These results suggest that genes in earlier diverging land plant lineages are able to function in a broader developmental context than those in later diverging lineages. Notably, the BELL gene phylogeny indicates that specialized function in ferns and flowering plants may have been achieved at least in part through modified KNOX‐BELL interactions.

## Materials and Methods

### Plant strains and growth conditions

The Gransden strain *Physcomitrella patens* subsp. *patens* (Engel, 1968) was grown and maintained under sterile conditions on BCD or BCDAT medium (Nishiyama *et al*., 2000) and grown at 24°C with a diurnal cycle of 16 h : 8 h, light (300 μmol m^−2^ s^−1^) : dark. For sporophyte induction, 1‐wk‐old protonemal tissue was transferred into Magenta pots containing 100 ml solidified BCD medium supplemented with 1 mM CaCl_2_, and grown at 25°C for 6 wk with a 16 h : 8 h, light (300 μmol m^−2^ s^−1^): dark cycle. Magenta pots containing tall gametophores were then transferred to 16°C, with an 8 h : 16 h, light (150 μmol m^−2^ s^−1^): dark cycle. Sterile water (5–6 ml) was added to each pot immediately after transfer to 16°C. Gametangia formation started within 2–3 wk and sporophytes were visible within 5–6 wk. The triple *mkn2;mkn4;mkn5* mutant (Sakakibara *et al*., 2008) was obtained from Mitsuyasu Hasebe, Okazaki, Japan. Arabidopsis (*Arabidopsis thaliana*) ecotypes Columbia and Landsberg erecta were grown at 22°C in a glasshouse with a 16 h : 8 h, light (300 μmol m^−2^ s^−1^) : dark cycle. The *bp*‐9 (Col) mutant (Smith & Hake, 2003) was obtained from the Nottingham Arabidopsis Stock Centre, UK. Conditions for growth of *Marchantia polymorpha*,* Spirogyra pratensis*,* Anthoceros agrestis*,* Selaginella kraussiana* and *Ceratopteris richardii* are provided in Supporting Information Methods S1.

### Phylogenetic analysis

KNOTTED‐LIKE HOMEOBOX (KNOX) and BELL homologous sequences were obtained by interrogating databases at NCBI (http://ncbi.nlm.nih.gov), Phytozome (http://phytozome.jgi.doe.gov) and OneKP (http://bioinfodata.org/Blast4OneKP) (Johnson *et al*., 2012; Wickett *et al*., 2014; Xie *et al*., 2014), plus rice (http://rice.plantbiology.msu.edu), *Klebsormidium* (http://www.plantmorphogenesis.bio.titech.ac.jp/~algae_genome_project/klebsormidium), *Amborella* (http://amborella.org) and spruce (http://congenie.org) genome projects, as well as in house genomic and transcriptomic resources for *Anthoceros punctatus*,* A. agrestis*,* M. polymorpha, C. richardii* and *S. pratensis* (Li *et al*., 2014; Szövényi *et al*., 2015; unpublished data), with a variety of KNOX or BELL homologues as Blast queries. Homology was assessed using reciprocal Blast and examination of alignments. *Selaginella kraussiana* BELL sequences were identified in this study (see Methods S1; Notes S1, S2).

Sequences were only included in the dataset if they comprised the conserved domains defined for each gene family: MEINOX, ELK and TALE‐HD domains for the KNOX family; SKY, PBC and TALE‐HD domains for the BELL family. *Marchantia polymorpha*,* S. pratensis* and *A. agrestis* KNOX sequences were verified by PCR amplification (see Methods S1; Notes S1) and dideoxy sequencing (Notes S2). In addition to species used in the KNOX dataset (Notes S3), sequences from *Picea abies* and *Amborella trichopoda* were added to the BELL dataset (Notes S4). A number of sequences from Arabidopsis, rice and *P. patens* could not be placed with confidence and consequently were excluded to improve overall tree robustness (listed in Notes S4). Putative homologues were identified in *S. pratensis* and other charophytes but these sequences appeared only remotely related to land plant BELL proteins, and when added to the dataset, showed very long branches and destabilized the resulting phylogenetic trees. As a consequence, charophyte sequences were not included in the final dataset. For the same reason, *Chlorophyta* sequences also were omitted. Because of these restrictions, the number of sequences for any particular species in the resulting phylogenies does not always reflect the total number of homologues in the genome of that species (Notes S3, S4).

KNOX protein alignment was achieved using Clustal Omega v.1.2.0 (Sievers *et al*., 2011) with 10 iterations and a HMM profile that was built using Pfam database alignments (http://pfam.xfam.org) of the MEINOX, ELK and TALE‐HD domains as a guide. The amino acids were then translated back to their corresponding codon sequences using a custom Perl script. BELL protein alignment was obtained using MergeAlign (Collingridge & Kelly, 2012) with default parameters. The aligned sequences were then edited and trimmed manually to conserve homologous sites only (Fig. S1; Notes S5, S6). Maximum‐likelihood trees were obtained with RAxML v.8.2.3 (Stamatakis, 2014) and Bayesian trees were obtained using MrBayes v.3.2.5 (Ronquist & Huelsenbeck, 2003) (full details in Methods S1).

### Generation of constructs for complementation in *Physcomitrella patens*

*MKN2* coding sequence was synthesized by Integrated DNA Technologies (Coralville, IA, USA). Remaining sequences were amplified from genomic DNA or by reverse transcription polymerase chain reaction (RT‐PCR) (Methods S1) and cloned into pJET1.2 before dideoxy sequencing. The p35S‐loxP‐BSD plasmid (NCBI no. AB537973) was used as a backbone for all *P. patens* transformation constructs. Constructs were designed to express *PpMKN2*,* SkKNOX1*,* SkKNOX2*,* CrKNOX1*,* CrKNOX2*,* BP or STM* genes under the control of the endogenous *PpMKN2* gene promoter (*MKN2pro:KNOX* constructs) (Fig. S2). During the generation of the triple *mkn2;mkn4;mkn5* mutant, the entire genomic sequence of *PpMKN2* and a 208‐bp fragment of 5′ *MKN2* flanking sequence was replaced by the G418 resistance cassette (Sakakibara *et al*., 2008). To reintroduce this 208‐bp fragment into the endogenous locus, it was included as part of the 5′ *MKN2* flanking sequence that was used as homologous sequence for recombination. A total of 1.211 kb of 5′ flanking sequence was fused with the coding sequence of *PpMKN2*,* SkKNOX1*,* SkKNOX2*,* CrKNOX1 CrKNOX2*,* BP* or *STM*. The *nos* terminator was fused 3′ to the coding sequence followed by the blasticidin resistance cassette and then a 1.833‐kb fragment of 3′ *MKN2* flanking sequence as homologous sequence for recombination. Expression of the blasticidin resistance gene was driven by the *35S CaMV* promoter.

### *Physcomitrella patens* transformation

*Physcomitrella patens* was transformed using the polyethylene glycol‐based transformation method (Schaefer *et al*., 1991). In five to seven independent transformation experiments, the *MKN2pro:KNOX* constructs were introduced into *mkn2;mkn4;mkn5* triple mutant protoplasts. Protoplasts were regenerated for 7 d and then transferred to fresh media containing antibiotics for selection (blasticidin at 75 mg ml^−1^). After 2 wk, plants were transferred back to nonselective BCDAT medium for another week and then stable transformants were selected by plating on selective media. After generating stable transgenic moss lines, each one was analysed by PCR to check that the construct was integrated into the desired locus (using primers listed in Notes S1). To determine the transgene copy number in each line, DNA gel blot analysis was carried out using 6 μg of genomic *P. patens* DNA according to Langdale *et al*. (1988). A 510‐bp fragment of the blasticidin resistance gene was hybridized to *NdeI* restricted genomic DNA – if the construct was correctly targeted to the *PpMKN2* locus, a single fragment was detected after hybridization. Genomic DNA from the *mkn2;mkn4;mkn5* triple mutant lines was used as a negative control.

### Expression analysis of *Physcomitrella patens* transformants by RT‐PCR

Total RNA was extracted from approximately 300 archegonia that contained either unfertilized eggs or early developing embryos. cDNA was synthesized using Superscript III reverse transcriptase (Invitrogen) and 500 ng of DNase treated (Turbo DNAse; Ambion) total RNA per sample. Primer pairs (Notes S1) designed to amplify a *c*.500‐bp fragment of the 5′ region of the Arabidopsis*, S. kraussiana* or *C. richardii* Class I KNOX genes, or to amplify *PpMKN2*, were used to check transcript accumulation. Amplification of the *Glycolytic glyceraldehyde‐3‐phosphate dehydrogenase* (*GAPC1*) gene (×72381) was used as an internal control (as used in Sakakibara *et al*., 2008). Each PCR was performed with both sets of primer pairs and adjusted so that the reactions were in the exponential phase. Optimization of RT‐PCR conditions to generate identical levels of *GAPC1* amplification was not possible due to the difficulty of extracting sufficient amounts of RNA.

### Arabidopsis transformation

Six constructs were designed to express *P. patens*,* S. kraussiana*,* C. richardii* and Arabidopsis Class I KNOX genes under the endogenous *BP* promoter. The final constructs (Fig. S3) consisted of the *BP* 5′ *cis*‐regulatory sequence (from −5474 bp to −1 bp relative to the ATG) fused to the coding sequence of *PpMKN2*,* SkKNOX1*,* SkKNOX2*,* CrKNOX1*,* CrKNOX2* or *BP*. The *octopine synthase* (*ocs3′*) terminator sequence was fused to the 3′ end of the various *KNOX* gene coding sequences. The *BPpro:KNOX:ocs3′* cassette was transferred as a *Not*I fragment into the binary vector pART27 (Gleave, 1992) and transformed into *bp*‐9 plants by floral dipping (Clough & Bent, 1998). Transformed plants were selected on the basis of kanamycin resistance and were self‐pollinated to generate T_1_, T_2_, and T_3_ populations that segregated the transgenes. Five T_3_ lines with a single transgenic copy were analysed for each combination of transgene/background, with the exception of *BPpro:SkKNOX1* and *BPpro:MKN2* for which 6 and 3 lines were analysed, respectively.

### Expression analysis of Arabidopsis transformants by qPCR

Transgene expression was analysed in Arabidopsis by quantitative real‐time reverse transcription‐PCR (qPCR). Total RNA was extracted from 12 inflorescences and then cDNA was synthesized using Superscript III reverse transcriptase (Invitrogen) and 1 μg of DNase treated (Turbo DNAse, Ambion) total RNA. Primer pairs were designed to amplify a *c*. 100‐bp fragment of the 3′ end of specific KNOX genes, with the exception of *PpMKN2* and *BP* which were designed to amplify the middle of the coding region (Notes S1). Amplification was detected using the SYBR Green master mix (Life Technologies, Carlsbad, CA, USA) according to manufacturer's instructions on a 7300 Real‐Time PCR System (Applied Biosystems, Foster City, CA, USA). Cycling conditions were: 95°C for 5 min, and 40 cycles of 95°C for 15 s and 60°C for 1 min. Three technical replicates were performed for three different biological samples of each independent line. The mean value between the two technical replicates with the closest values was calculated. Two housekeeping genes, *EF1Balpha2* (At5g19510) and *UBP6* (At1g51710) were used as constitutive controls. To test for genomic DNA contamination, primers that amplify only the genomic sequence of *EF1Balpha2* (At5g19510) were used (Wang *et al*., 2007).

Data analysis was performed using the LinRegPCR program (Ramakers *et al*., 2003). LinRegPCR calculates a value called *N*_0_, expressed in arbitrary fluorescence units, which reflects the initial amount of template in the reaction mix, using the following formula: *N*_0_ = *N*_q_/*E*_m_^Cq^. *N*_q_ is the fluorescence threshold, *C*_q_ is the cycle number at which the PCR reaction reaches *N*_q_, and *E*_m_ is the mean PCR efficiency for a particular primer pair amplicon. Samples were normalized using the geometric mean of the expression values of *EF1BalphaA2* and *UBP6* reference genes. Finally, *N*_0_ values obtained from the reactions that amplify genomic sequence of *EF1Balpha2,* reflecting genomic contamination, were subtracted from *N*_0_ value for each line.

### Silique angle measurement

Measurements were performed on 7‐wk‐old plants. Silique angles on the main inflorescence were measured for 10 plants per line using a protractor. Statistical analyses were carried out as in Methods S1 and Notes S7.

### Microscopy

*Physcomitrella patens* images were captured using a QImaging Micropublishing 5.0 RTV camera on a Leica M165C microscope. Photographs of 30 sporophytes were taken and then the surface area of the sporophyte was compared with the area occupied by spores. Area measurements were carried out using the ImageJ software (http://rsb.info.nih.gov/ij/). The mean of the sporophyte area divided by the area occupied by spores was calculated for each line.

## Results

### KNOX genes duplicated before the evolution of land plants

To date, liverwort and hornwort sequences have not been included in KNOX gene phylogenies, and in the one example where charophyte sequences have been included (Sakakibara, 2016) the topology contradicted a better‐supported tree that defined distinct *Chlorophyta*, KNOX1 and KNOX2 clades (Furumizu *et al*., 2015). To better define relationships in early diverging lineages, transcriptome and/or genome data from the charophyte alga *S. pratensis*, the liverwort *M. polymorpha*, and the hornworts *A. punctatus* (Li *et al*., 2014) and *A. agrestis* (Szövényi *et al*., 2015) were searched for KNOX‐homologous sequences using TBlastN and BlastP algorithms and a variety of KNOX homologues as queries. Retrieved sequences were classified as KNOX genes if they encoded both a MEINOX domain and a TALE‐HD, using careful sequence examination of alignments and reciprocal Blast (a number of MEINOX‐only sequences from *M. polymorpha* were discarded). A single KNOX gene sequence was identified in both *A. punctatus* and *A. agrestis*, whereas two gene sequences were identified in both *S. pratensis* and *M. polymorpha*. To validate gene models, coding sequences were confirmed by sequencing PCR‐amplified cDNA (Notes S2). Notably, the annotation model for the *MpKNOX2* gene includes a long repetitive region between the MEINOX and ELK domains that could not be amplified by PCR (Notes S2). As this region could not be validated molecularly, it was excluded from subsequent alignments used for tree inference. In summary, a single KNOX gene was verified in each of the hornwort genomes, whereas two genes were verified in the charophyte *S. pratensis* and the liverwort *M. polymorpha*.

**Figure 1:**
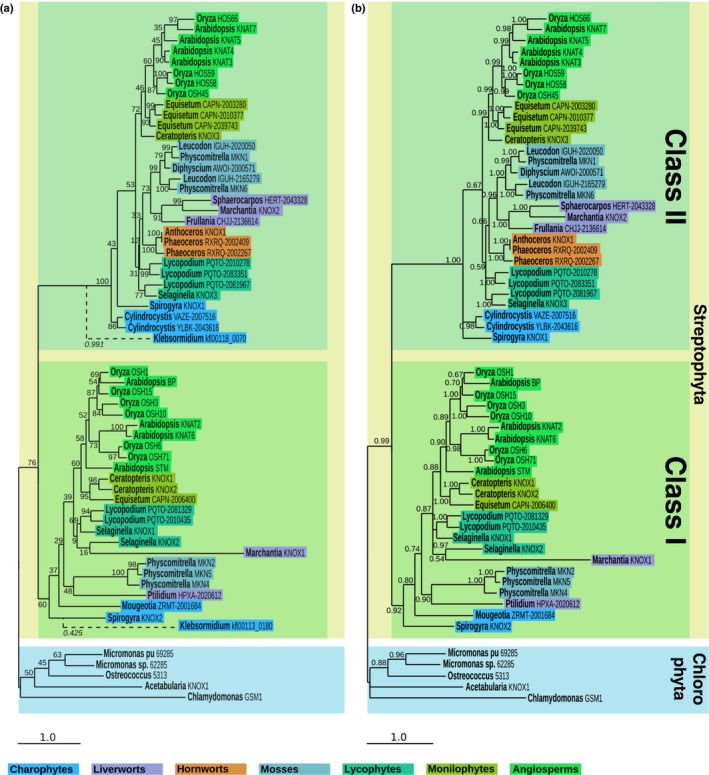
Maximum‐likelihood and Bayesian phylogenetic analysis of KNOTTED‐LIKE HOMEOBOX (KNOX) homologues. (a, b) Phylogenetic trees inferred with (a) RA x ML or (b) MrBayes, using a partitioned nucleotide dataset of 59 + 2 sequences (Supporting Information Fig. S1) evolving under the GTR + Γ + I model. Support values (a, bootstrap values; b, posterior probabilities) are indicated next to the corresponding branch. To avoid destabilization at the base of the Class I clade by *Klebsormidium flaccidum* sequences, kfl00113_0180 and kfl00118_0070 were added to the maximum‐likelihood tree after the phylogeny was inferred using the evolutionary placement algorithm of RAxML. As a consequence, *K*. *flaccidum* sequence branches are dashed and their associated likelihood weight ratios (Stamatakis, 2014) are indicated in italics. Each sequence was placed on its recipient tree branch with respect to the actual distance derived from a maximum likelihood tree inferred using all 61 sequences (Fig. S2). Colour‐coded boxes correspond to the species’ phyla. Both trees were rooted using the *Chlorophyta* clade as an outgroup.

Phylogenetic analyses were carried out using the five newly identified sequences from *S. pratensis*,* M. polymorpha* and *A. agrestis*, all of the KNOX sequences identified in the model flowering plants Arabidopsis (eight genes) and rice (10 genes) (Mukherjee *et al*., 2009), and sequences that have previously been molecularly validated in the monilophyte fern *C. richardii* (Sano *et al*., 2005), the lycophyte *S. kraussiana* (Harrison *et al*., 2005), the moss *P. patens* (Sakakibara *et al*., 2008) and the chlorophyte algae *A. acetabulum* (Serikawa & Mandoli, 1999) and *C. reinhardtii* (Lee *et al*., 2008). To provide a more balanced species distribution, additional nonflowering plant and charophyte sequences were retrieved from the OneKP dataset (Matasci *et al*., 2014) and from the *Klebsormidium* genome project database (Hori *et al*., 2014). Further chlorophyte sequences were retrieved from the Phytozome database (Notes S3). After alignment of protein sequences and reverse translation back to nucleotide sequence (Fig. S1; Notes S5), both maximum‐likelihood and Bayesian trees were inferred (Figs 1, S4). The topology of both trees essentially reflected the species tree, with three statistically well‐supported KNOX gene clades – *Chlorophyta*, Class I and Class II. Notably, charophyte sequences are present in both Class I and Class II clades, whereas hornwort sequences are restricted to Class II. The duplication leading to Class I and Class II KNOX genes therefore occurred within the charophyte sister group to land plants, with subsequent events in the bryophytes leading to conserved Class II sequences but substantially different (or absent) Class I sequences.

### Vascular plant Class I KNOX genes are unable to complement loss‐of‐function mutations in the moss *Physcomitrella patens*

**Figure 2:**
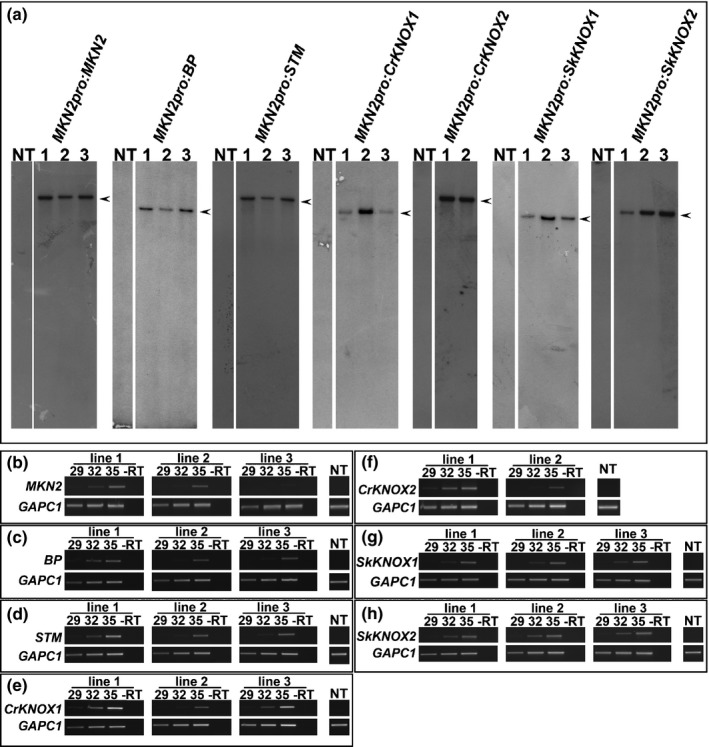
Transgene integration and expression in *Physcomitrella patens mkn* triple mutants. (a) DNA gel blot analysis of *Nde*I digested genomic DNA from *mkn2;mkn4;mkn5* mutants transformed with *MKN2pro:KNOX* constructs. Blots were hybridized with a fragment of the blasticidin resistance gene present in the transgene construct (see Supporting Information Fig. S2). Hybridizing fragments were not detected in DNA samples from nontransformed mutants (NT), whereas 11.8‐kb (*MKN2pro:MKN2*), 10‐kb (*MKN2pro:BP*), 11.59‐kb (*MKN2pro:STM*), 9.9‐kb (*MKN2pro:CrKNOX1*), 11.78‐kb (*MKN2pro:CrKNOX2*), 9.42‐kb (*MKN2pro:SkKNOX1*) and 9.72‐kb (*MKN2pro:SkKNOX2*) fragments were detected in transgenic lines. (b–h) reverse transcription polymerase chain reaction (RT‐PCR) showing transgene transcript accumulation in *mkn2;mkn4;mkn5* mutant plants transformed with (b) *MKN2pro:MKN2*, (c) *MKN2pro:BP*, (d) *MKN2pro:STM*, (e) *MKN2pro:CrKNOX1*, (f) *MKN2pro:CrKNOX2*, (g) *MKN2pro:SkKNOX1* and (h) *MKN2pro:SkKNOX2*. *GAPC1* was used as an amplification and loading control in each case. Amplifications were performed for 29, 32 and 35 cycles (as indicated) to ensure amplification in the exponential phase. Negative control reactions were carried out in the absence of reverse transcriptase (‐RT) and with cDNA isolated from nontransformed triple mutants (NT).

**Figure 3:**
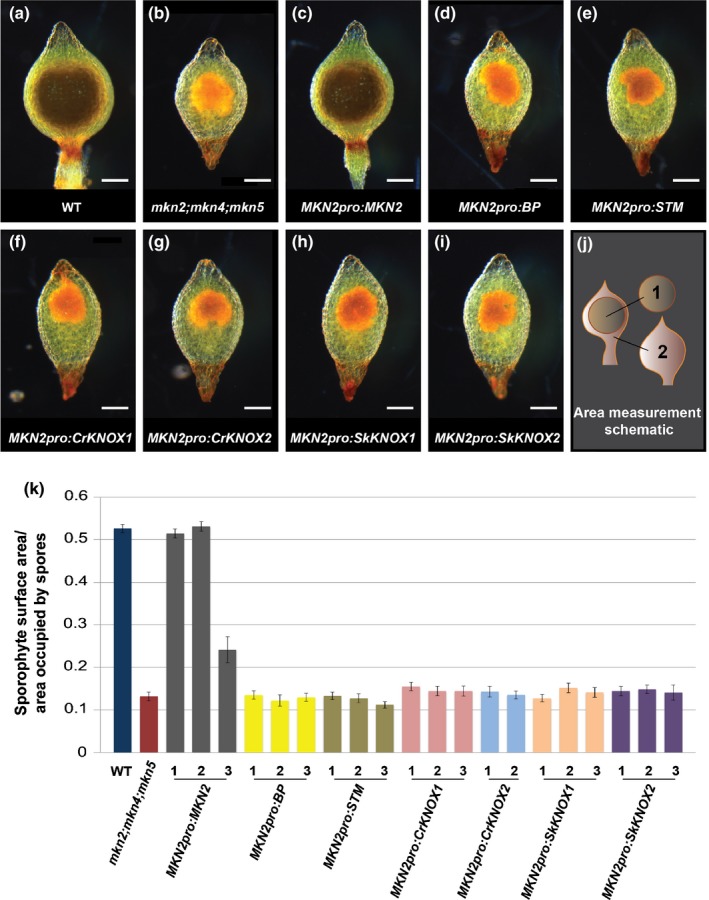
Cross‐species complementation tests with *Physcomitrella patens mkn* triple mutants. (a–i) Representative sporophyte phenotype of (a) wild‐type (WT), (b) *mkn2;mkn4;mkn5* mutant, and (c–i) *mkn2;mkn4;mkn5* mutants transformed with (c) *MKN2pro:MKN2*, (d) *MKN2pro:BP*, (e) *MKN2pro:STM*, (f) *MKN2pro:CrKNOX1*, (g) *MKN2pro:CrKNOX2*, (h) *MKN2pro:SkKNOX1* and (i) *MKN2pro:SkKNOX2* constructs. Bars, 200 μm. (j, k) Schematic depiction of measurements taken (j) and average ratios of sporophyte surface area to the area occupied by spores (k) for the lines exemplified in (a–i). Thirty sporophytes from each of three independent transformed lines were analysed for each construct, with the exception of *MKN2pro:CrKNOX2* for which only two independent lines were recovered. Error bars represent ± SEM.

In order to determine the extent to which Class I KNOX gene function diverged beyond the bryophytes, experiments were designed to test whether Class I KNOX genes from vascular plants can complement loss‐of‐gene‐function in *P. patens*. Sporophyte defects in triple loss‐of‐function *mkn2;mkn4;mkn5* mutants are more severe than those in *mkn4* and *mkn5* single mutants but are very similar to those in single *mkn2* mutants (Sakakibara *et al*., 2008). This suggests that *PpMKN2* plays a more substantial role in sporophyte development than the other two Class I KNOX genes. To test this, a construct was designed to replace the G418 marker in the disrupted *mkn2* locus of the triple mutant with a functional *PpMKN2* gene and a blasticidin selectable marker (Fig. S2). Homologous recombination should lead to both the *PpMKN2* coding sequence and the blasticidin resistance gene being driven by the endogenous *PpMKN2* promoter, with both genes being flanked by the endogenous *PpMKN2* 3′ sequence. Three independent transgenic lines were selected on blasticidin and a single insertion of the expected size was confirmed in each line by DNA gel blot analysis (Fig. 2a). RT‐PCR analysis confirmed that the *PpMKN2* coding sequence was expressed in each case (Fig. 2b) and a comparison between the phenotype of wild‐type (Fig. 3a), triple mutant (Fig. 3b) and triple mutant plus the *MKN2pro:MKN2* transgene (Fig. 3c) lines demonstrated that sporophyte size and morphology was restored by the presence of the functional *PpMKN2* gene (Fig. 3j,k), with the extent of complementation broadly correlated with transgene expression level (compare Figs 2b, 3k). Loss‐of‐function in all three *P. patens* Class I KNOX genes can thus be complemented by *PpMKN2* alone.

In order to determine whether Class I KNOX genes from vascular plant lineages can substitute for *PpMKN2* function in *P. patens*, constructs analogous to that used for *PpMKN2* were designed for homologous recombination with two of the four Class I KNOX genes in Arabidopsis (*BP* and *SHOOTMERISTEMLESS*–*STM*), the two genes in *C. richardii* (*CrKNOX1* and *CrKNOX2*) and the two genes in *S. kraussiana* (*SkKNOX1* and *SkKNOX2*) (Fig. S2b–g). As with *MKN2pro:MKN2*, three independent transgenic lines were selected for each construct (with the exception of *MKN2pro:CrKNOX2* for which only two lines regenerated), all were confirmed by DNA gel blot analysis to have single transgene insertions of the expected size (Fig. 2a) and all showed transgene expression (Fig. 2c–h). Unlike *MKN2pro:MKN2* lines, however, sporophyte phenotypes in lines expressing genes from the vascular plant lineages were the same as in the triple mutant (Fig. 3d–i). Quantification of the total sporophyte area and the area occupied by spores (Fig. 3j,k) confirmed the lack of complementation in each case. As such, Class I KNOX genes from vascular plants cannot substitute for *PpMKN2* function in *P. patens*.

### Class I KNOX genes from *Physcomitrella patens* and *Selaginella kraussiana* fully complement loss‐of‐function mutations in the Arabidopsis *BP* gene – but those from *Ceratopteris richardii* do not

**Figure 4:**
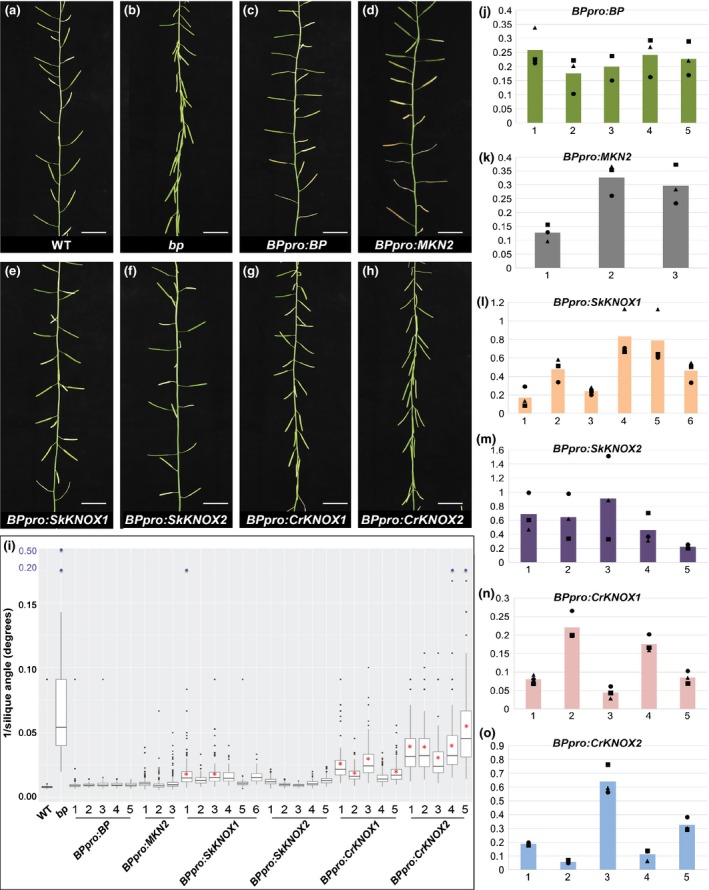
Cross‐species complementation tests with Arabidopsis *bp‐9* mutants. (a–h) Representative inflorescence phenotype of (a) wild‐type (WT), (b) *bp‐9* mutant, and (c–h) homozygous *bp‐9* mutant plants transformed with (c) *BPpro:BP*, (d) *BPpro:MKN2*, (e) *BPpro:SkKNOX1*, (f) *BPproSkKNOX2*, (g) *BPpro:CrKNOX1* and (h) *BPpro:CrKNOX2* constructs. Bars, 2 cm. (i) Boxplot showing range of silique angles on the primary inflorescence of lines exemplified in (a–h). Primary inflorescences from 10 plants of at least three independent lines per transgene were analysed. Red asterisks indicate lines that are significantly different (*P *<* *0.05) from WT. All lines are significantly different (*P *<* *0.05) from *bp‐9* mutants. Note that blue points on the *y*‐axis are not to scale relative to those in black. (j–o) Quantitative reverse transcription polymerase chain reaction (qRT‐PCR) analyses of (j) *BPpro:BP*, (k) *BPpro:MKN2*, (l) *BPpro:SkKNOX1*, (m)*BPpro:SkKNOX2*, (n) *BPpro:CrKNOX1* and (o) *BPpro:CrKNOX2* transgene expression. Numbers on the *x*‐axis represent independent transgenic lines as indicated in (i). The *y*‐axis represents transcript levels (*N*_0_) normalized against the *EF1Balpha2* and *UBP6* genes. Three technical replicates were performed for three different biological samples of each independent line. Scatter blots represent values of individual biological replicates; bars represent mean of three biological replicates.

Class I KNOX genes from vascular plants may be unable to complement loss‐of‐function moss mutants because inherently distinct roles have evolved in the two groups. To test this hypothesis, the ability of *PpMKN2* to complement loss‐of‐function Arabidopsis mutants was determined. Ideally, both *stm* and *bp* mutants would be analysed but loss‐of‐function *stm* mutants exhibit severe and/or often pleiotropic shoot defects that are extremely difficult to quantify (Barton & Poethig, 1993; Endrizzi *et al*., 1996). *bp* mutants can also exhibit a range of different shoot phenotypes; however, the *bp‐9* allele has a consistent mutant phenotype whereby cell division defects in the developing inflorescence lead to downward‐pointing siliques (Venglat *et al*., 2002) (Fig. 4a). Experiments were thus carried out with the *bp‐9* allele. Quantification of silique angles demonstrated that the mutant phenotype was fully rescued by a construct comprising the full‐length *BP* coding sequence driven by 5.474 kb of the endogenous promoter (Figs 4c,i, S3). This observation demonstrated that the 5.474‐kb sequence is sufficient to ensure appropriate temporal and spatial regulation of *BP* gene expression during shoot development. The same promoter sequence was therefore used to generate constructs for transgenic expression of *PpMKN2* (Fig. S3). Analysis of three lines showed that when the *pBPpro:MKN2* transgene was expressed (Fig. 4k), the *bp‐9* mutant phenotype was fully complemented (Fig. 4d,i). As such, PpMKN2 is not inherently different from BP in that it can activate or repress targets (either directly or indirectly) that are misexpressed in *bp‐9* mutants, and in so doing restore normal cell division patterns.

Given that PpMKN2 shares only 35% amino acid identity with BP whereas the vascular plant proteins share 43% (SkKNOX2) to 52% (CrKNOX1) identity, we predicted that the lycophyte and fern genes also would complement *bp‐9* mutants. To test this prediction, transgenic *bp‐9* mutant lines were generated in which expression of full‐length *SkKNOX1, SkKNOX2, CrKNOX1* or *CrKNOX2* coding sequence was driven from the 5.474‐kb *BP* promoter (Fig. S3). Analysis of at least five independent lines for each transgene demonstrated that both *BPpro:SkKNOX1* and *BPpro:SkKNOX2* can fully rescue the mutant phenotype (Fig. 4e,f,i). Quantitative RT‐PCR revealed that the extent of phenotypic rescue roughly correlated with the amount of transcript present in the inflorescence (compare Fig. 4i,l,m), with lines accumulating the most transcript showing a similar level of complementation to lines expressing the *BPpro*:*BP* construct. By contrast, *BPpro:CrKNOX1* and *BPpro:CrKNOX2* constructs showed only partial rescue of the *bp‐9* mutant phenotype (Fig. 4g–i), even when relatively high levels of transgene transcripts were detected (compare Fig. 4i,n,o). These results suggest that despite the fern KNOX proteins being more similar in sequence to BP, the lycophyte proteins are better able to regulate BP targets in the context of the Arabidopsis silique development.

### Distinct evolutionary trajectories for BELL proteins in lycophytes and euphyllophytes

**Figure 5:**
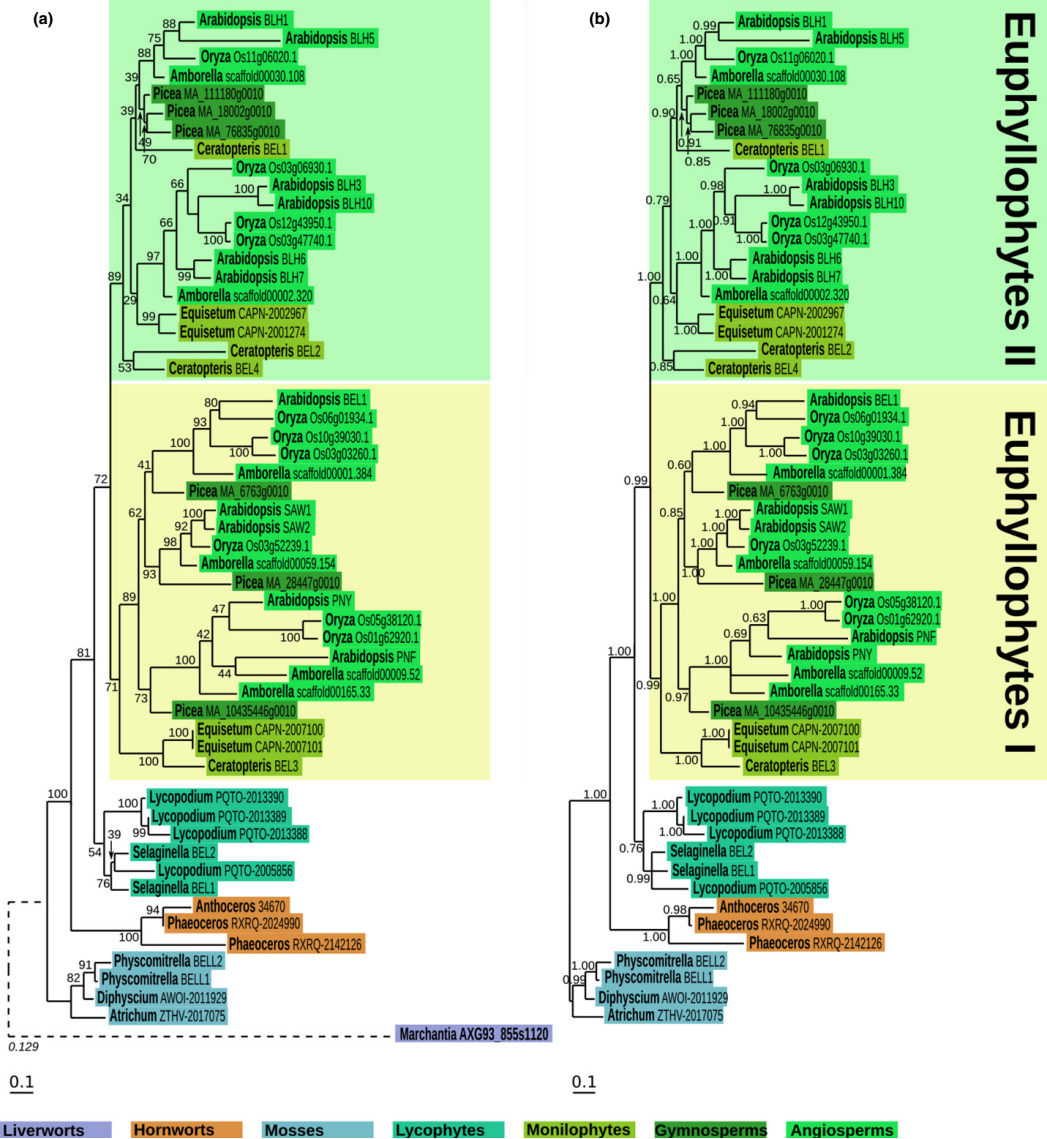
Maximum‐likelihood and Bayesian phylogenetic analysis of BELL homologues. (a, b) Phylogenetic trees inferred with (a) RA x ML or (b) MrBayes, using a protein alignment evolving under the JTT + Γ + I model. Support values (a, bootstrap values; b, posterior probabilities) are indicated next to the corresponding branch. In order to avoid destabilization of the early divergent clades, the identified BELL homologue in *Marchantia polymorpha* was placed using the evolutionary placement algorithm of RA x ML and the maximum‐likelihood tree as recipient. As a consequence, the *M. polymorpha* BELL sequence branch is dashed and the associated likelihood weight ratio (Stamatakis, 2014) is indicated in italics. Colour coded boxes correspond to the species phyla. (a) The maximum‐likelihood tree was rooted using the *M. polymorpha* BELL homologue and (b) the Bayesian tree was rooted using the moss clade.

In order to determine whether the differential ability of lycophyte and fern KNOX proteins to complement the Arabidopsis *bp‐9* mutant could reflect conserved vs divergent molecular interactions, existing BELL gene phylogenies (Mukherjee *et al*., 2009; Furumizu *et al*., 2015; Sakakibara, 2016) were examined for evidence of coevolution. Unfortunately, the critical lycophyte/fern distinction could not be examined because none of the published phylogenies included fern sequences. BELL gene sequences were therefore aligned and used to infer phylogeny (see the Materials and Methods section; Methods S1; Notes S4, S6). The resultant maximum‐likelihood and Bayesian phylogenies both align well with the species tree and both resolve single clades in each bryophyte group and in the lycophytes, but two distinctive clades in the euphyllophytes (Euphyllophytes I and Euphyllophytes II) (Fig. 5). The topology of the BELL phylogeny indicates that a gene duplication event predated the divergence of the monilophytes and seed plants, and that copies of both duplicates have been retained in each lineage. There is thus a clear lycophyte/fern distinction in the BELL phylogeny.

## Discussion

Using newly acquired genome and transcriptome datasets, supported by molecular validation of gene models, we have generated KNOTTED‐LIKE HOMEOBOX (KNOX) and BELL gene phylogenies that have refined the timing of the duplication that led to distinct Class I and Class II KNOX genes, and identified a BELL gene duplication before the divergence of monilophytes and seed plants that distinguishes bryophyte and lycophyte lineages from euphyllophytes. The integration of these phylogenetic data with the results of reciprocal cross‐species complementation experiments reveals an evolutionary trajectory whereby KNOX function becomes more specialized as progressively more gene duplications occur and lineages diverge. Notwithstanding the limitations of heterologous complementation assays, particularly where it is only feasible and/or practical to analyse the activity of single genes from multigene families, this study provides the most comprehensive functional comparison of Class I KNOX genes to date, in that it incorporates genes from all of the major land plant lineages.

### Class I and Class II KNOX genes in charophytes

The discovery of Class I and Class II KNOX genes in the charophyte alga *Spirogyra pratensis,* plus confirmation of single KNOX gene sequences in all chlorophyte algae examined (Fig. 1) refines the timing of the first KNOX duplication event and raises a question about the ancestral function of the two gene classes. It has previously been suggested that the ability to balance activity between Class I and Class II gene function enabled the alternation of generations to evolve in land plants (Sakakibara *et al*., 2013; Furumizu *et al*., 2015). Whilst this may be correct, KNOX genes must play a different role in charophyte algae because the diploid phase of the lifecycle is invariantly restricted to the unicellular zygote. Possibly the gene duplication facilitated multicellularity in the gametophyte, a suggestion supported by the observation that transcriptomes of the unicellular charophyte *Cylindrocystis* contains just a Class II gene whereas both classes are present in the filamentous charophytes *S. pratensis* and *Klebsormidium flaccidum* (Figs 1, S4). The presence of the two gene classes in the *K. flaccidum* genome further infers that the duplication occurred before the divergence of Charales, Zygnemetales and Coleochaetales. As such, the duplication may have occurred concomitant with the chlorophyte/streptophyte divergence, but genome sequence from *Clorokybus* is needed to confirm or refute this suggestion because existing transcriptome datasets (Timme *et al*., 2012) do not contain KNOX gene sequences.

### Divergent Class I KNOX genes in bryophytes

To date, KNOX gene function in bryophytes has been characterized only in the moss *Physcomitrella patens*, where *PpMKN2* is expressed in the gametophyte, and after fertilization all three Class I genes regulate cell proliferation in the developing sporophyte (Singer & Ashton, 2007; Sakakibara *et al*., 2008; Frank & Scanlon, 2015a,b; Horst *et al*., 2016). Class II genes also are expressed in the sporophyte, where they are proposed to suppress the gametophyte developmental program (Sakakibara *et al*., 2013). Both Class I and Class II genes have been identified in liverworts, although at least in *Marchantia polymorpha* there is substantial sequence divergence in the Class I gene as evidenced by poor sequence alignment, a very long branch, and unsupported placement in the lycophyte clade (Figs 1, S1, S4). The failure to identify Class I genes in the three hornwort species examined suggests even greater sequence divergence in this group, or even gene loss. These observations are surprising given the phylogenetic position of mosses in relation to the other two bryophyte groups. Possibly the role of Class I KNOX genes in the *P. patens* sporophyte is a moss (or even *P. patens*)‐specific trait within the bryophytes. If this is the case, given that Class I KNOX genes also regulate cell proliferation in vascular plant sporophytes, this role either evolved independently in mosses and vascular plants, substantially diverged and/or was lost twice independently in liverworts and hornworts or, contrary to current phylogenetic interpretations (Ligrone *et al*., 2012b; Cox *et al*., 2014; Wickett *et al*., 2014), mosses are the sister group to vascular plants.

### Evolution of KNOX function

Reciprocal cross‐complementation experiments have revealed that the Class I KNOX gene *PpMKN2* from *P. patens* is able to complement the Arabidopsis *bp‐9* mutant when expressed in the endogenous *BP* expression domain (Fig. 4), whereas BP cannot substitute for PpMKN2 function in *P. patens* (Figs 2, 3). As such, it can be concluded that PpMKN2 is able to activate the same downstream gene targets as BP, either on its own or as a heterodimer with Arabidopsis BELL proteins, but that BP is unable to activate PpMKN2 downstream targets. This distinction suggests that sub‐ or neofunctionalization of Class I genes in Arabidopsis has constrained the activity of BP relative to PpMKN2. Examples of both subfunctionalization (stomatal development, MacAlister & Bergmann, 2011; ABA signalling, Marella *et al*., 2006) and neofunctionalization (LEAFY function, Maizel *et al*., 2005) during the evolutionary transition from bryophytes to flowering plants have been reported previously. However, in most cases, whereas complementation of Arabidopsis mutants by homologous *P. patens* genes have been reported (e.g. in root hair development (Menand *et al*., 2007), stomatal opening (Chater *et al*., 2011) and photomorphogenesis (Ranjan *et al*., 2014)), reciprocal experiments with moss mutants were not carried out and thus no trend could be inferred. An exception to this was reported for transcription factors that regulate chloroplast development. In this case, as with Class I KNOX genes, a *P. patens GOLDEN2‐LIKE* gene complemented an Arabidopsis loss‐of‐function mutant (Yasumura *et al*., 2005), whereas an Arabidopsis gene could not rescue a *P. patens* mutant (Bravo‐Garcia *et al*., 2009).

The inability of Class I KNOX genes from any of the vascular plant lineages to complement the *P. patens mkn2;mkn4;mkn5* mutant (Figs 2, 3) is striking because it suggests divergence of gene function after the bryophyte/lycophyte divergence. However, even though the lycophyte genes *SkKNOX1* and *SkKNOX2* cannot substitute for *PpMKN2* in moss, both lycophyte genes complement the *bp‐9* mutant to the same extent as *PpMKN2* (Fig. 4; Notes S7). This observation indicates an equivalent ability to regulate downstream targets of BP in Arabidopsis (either directly or indirectly), which in the case of promoting appropriate cell division patterns in the silique, requires repression of the Class I genes *KNAT2* and *KNAT6* (Li *et al*., 2012). By contrast, the monilophyte fern genes *CrKNOX1* and *CrKNOX2* only partially complement the *bp‐9* mutant phenotype (Fig. 4; Notes S7). Previous reports have suggested that *PpMKN2* (Sakakibara *et al*., 2008), *CrKNOX* (Sano *et al*., 2005) and *BP* (Lincoln *et al*., 1994) genes function equivalently in Arabidopsis because in each case constitutive expression leads to a lobed leaf phenotype. However, these reports may have overlooked the functional distinction between PpMKN2 and CrKNOX because of the contextual difference between ectopic KNOX activity in the leaf vs loss of endogenous activity in the shoot.

### Evolution of KNOX/BELL interactions

Because KNOX proteins function by dimerizing with BELL proteins in both chlorophyte algae (Lee *et al*., 2008) and flowering plants (reviewed in Hay & Tsiantis, 2010), changes in KNOX protein structure and function must have influenced, and been influenced by, changes in BELL protein structure and function. Despite this assumption, there is little evidence of co‐evolution in the gene phylogenies (Figs 1, 5; Furumizu *et al*., 2015). KNOX genes underwent a duplication event within the charophytes, and then any subsequent duplications were phylum‐ or species‐specific with structural motifs clearly conserved throughout the green lineage. In contrast to the conserved structure of KNOX proteins, BELL protein structure is highly variant in both chlorophyte and charophyte algae, with clearly recognizable domains only apparent in land plants. Previous phylogenies nested lycophyte BELL sequences within a flowering plant clade (Furumizu *et al*., 2015) but by increasing the taxon sampling to include ferns, our data have revealed that BELL gene duplications were phylum‐ or species‐specific before the divergence of lycophytes and euphyllophytes. As such, the multiple BELL genes in hornworts, mosses and lycophytes are all derived from a single ancestral sequence at the base of each lineage. By contrast, a gene duplication before the divergence of monilophytes and seed plants resulted in two gene families in euphyllophytes (Euphyllophytes I and II; Fig. 5). Notably the euphyllophyte duplication may have provided the potential for monilophyte and seed plant KNOX proteins to evolve more restricted interactions with BELL proteins, as compared with their lycophyte and bryophyte counterparts. As such, the inability of CrKNOX genes to fully complement *bp‐9* mutants could reflect restricted capacity (as compared with BP, SkKNOX1/2 and MKN2) to interact with the Arabidopsis BELL proteins that partner BP. This suggestion may seem counterintuitive given that CrKNOX proteins share the greatest amino acid identity with BP (50%, 48%, 44%, 40% and 44% for CrKNOX1, CrKNOX2, SkKNOX1, SkKNOX2 and MKN2, respectively) in the MEINOX domain that interacts with BELL proteins. However, *in silico* secondary structure analyses (http://www.sbg.bio.ic.ac.uk/phyre2/; Kelley *et al*., 2015) predict the folding of two alpha helices in the MEINOX domain of BP, SkKNOX1, SkKNOX2 and MKN2, but predict the presence of just a single helix in CrKNOX2 and only weakly support the presence of a second helix in CrKNOX1 (Fig. S5). The suggestion that differential complementation capacity results from differential ability to form KNOX/BELL dimers needs to be confirmed experimentally, but given that the results of *in vitro* and heterologous interaction assays are often conflicting depending on the assay used, or have been overturned by *in vivo* genetic experiments (Furumizu *et al*., 2015), it is not a trivial hypothesis to validate and thus must remain speculation at this stage.

## Author contributions

E.F. conducted Arabidopsis and moss complementation plus hornwort KNOX gene isolation; D.S‐M. conducted Spirogyra and Marchantia transcriptomes/gene isolation plus phylogenetic analysis; L.A.M. conducted BELL gene isolation; E.R. conducted plant culture and maintenance plus RT‐PCR analyses; J.A.L. conducted initial project design; and E.F., L.A.M., D.S‐M. and J.A.L. conducted data analysis and manuscript preparation.

## Supporting information

Please note: Wiley Blackwell are not responsible for the content or functionality of any Supporting Information supplied by the authors. Any queries (other than missing material) should be directed to the *New Phytologist* Central Office.

**Fig. S1** Graphical representation of KNOX alignment showing KNOX conserved domains and partitioning used for inferring phylogeny.
**Fig. S2** Construct design for homologous recombination of KNOX genes in the *Physcomitrella patens MKN2* locus.
**Fig. S3** Schematic of *BPpro:KNOX* constructs for *bp* complementation experiments.
**Fig. S4** KNOX maximum‐likelihood phylogeny including *Klebsormidium* sequences.
**Fig. S5** Predicted secondary structure of the MEINOX domain.

**Methods S1** Supplementary details for methods.

**Notes S1** List of primers used.

**Notes S2** Verified *Marchantia polymorpha*,* Spirogyra pratensis* and *Anthoceros agrestis* KNOX plus *Selaginella kraussiana* BELL sequences.

**Notes S3** Source and accessions of KNOX sequences used for phylogenetic analysis.

**Notes S4** Source and accessions of BELL sequences used for phylogenetic analysis.

**Notes S5** Edited KNOX alignment.

**Notes S6** Edited BELL alignment.

**Notes S7** Silique angle measurements and results of Tukey's HSD tests.
