# Supplementary material for: Nonreciprocal complementation of KNOX gene function in land plants
Source: New Phytol. 2016 Nov 25;216(2):591–604. doi: 10.1111/nph.14318 (PMC5637896; doi:10.1111/nph.14318)

NON-RECIPROCAL COMPLEMENTATION OF KNOX GENE FUNCTION IN LAND PLANTS

Eftychios Frangedakis, Denis Saint-Marcoux, Laura A. Moody, Ester Rabbinowitsch and Jane A. Langdale

Article acceptance date: 30 September 2016

Supporting Figure S1.
Graphical representation of KNOX alignment showing conserved domains and partitioning used for inferring phylogeny.

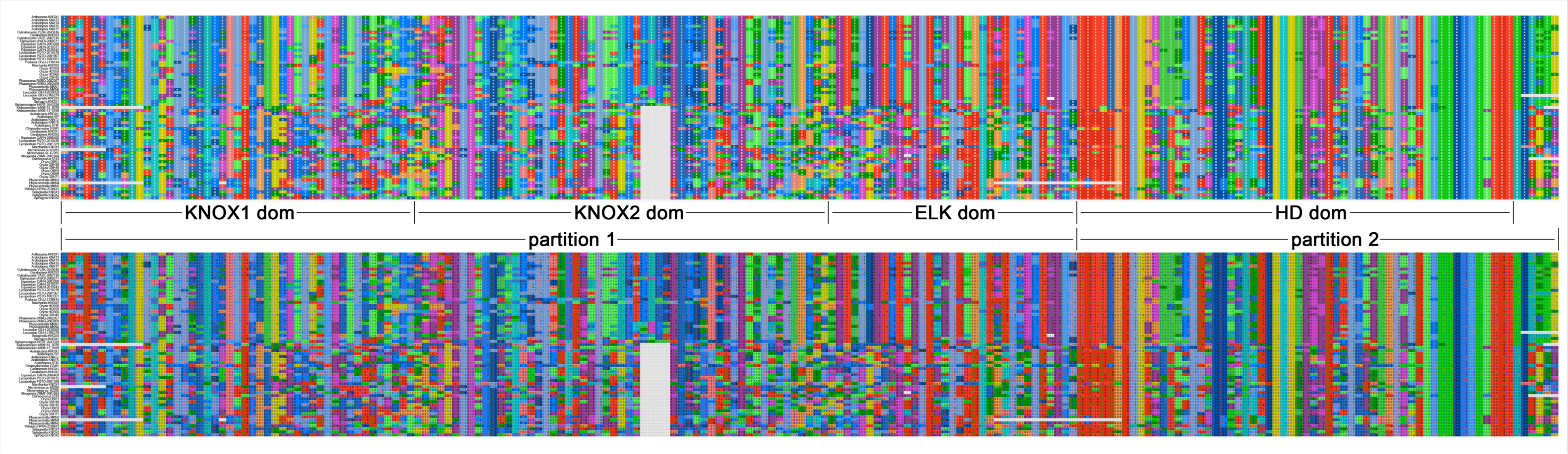

Supporting Figure S2. Construct design for homologous recombination of KNOX genes into the *P. patens MKN2* locus. a-g) Schematic representation of *MKN2pro:MKN2* (a), *MKN2pro:BP* (b), *MKN2pro:STM* (c), *MKN2pro:CrKNOX1* (d), *MKN2pro:CrKNOX2* (e), *MKN2pro:SkKNOX1* (f), *MKN2pro:SkKNOX2* (g) constructs. *MKN2 5'* (blue boxes) and *3'* (dark purple boxes) flanking regions, the different KNOX gene coding sequences (coloured arrows), the *nos* terminator (black boxes) and the blasticidin resistance cassette (dark blue arrow) are indicated. The re-introduced 208 bp fragment of 5' *MKN2* flanking sequence that was replaced by the G418 resistance cassette in the *mkn2* knockout mutant is indicated by a red box. *Nde*I restriction sites are represented with the capital letter "N" and the size of expected fragments after genomic insertion indicated. The probe used for DNA blot hybridization in Figure 2a is represented with a blue box above the blasticidin cassette.

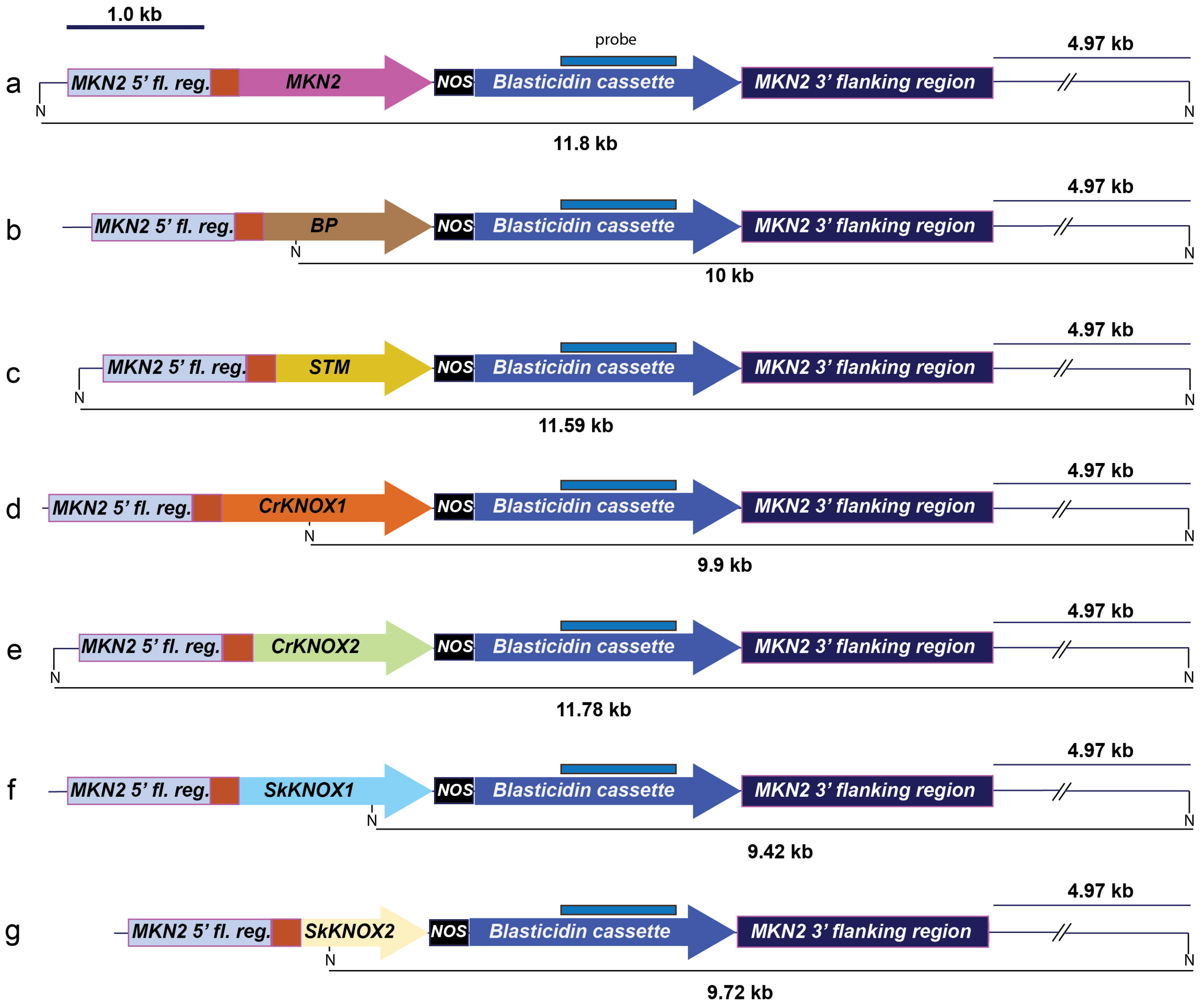

Supporting Figure S3. Schematic of *BPpro:KNOX* constructs for *bp* complementation experiments.

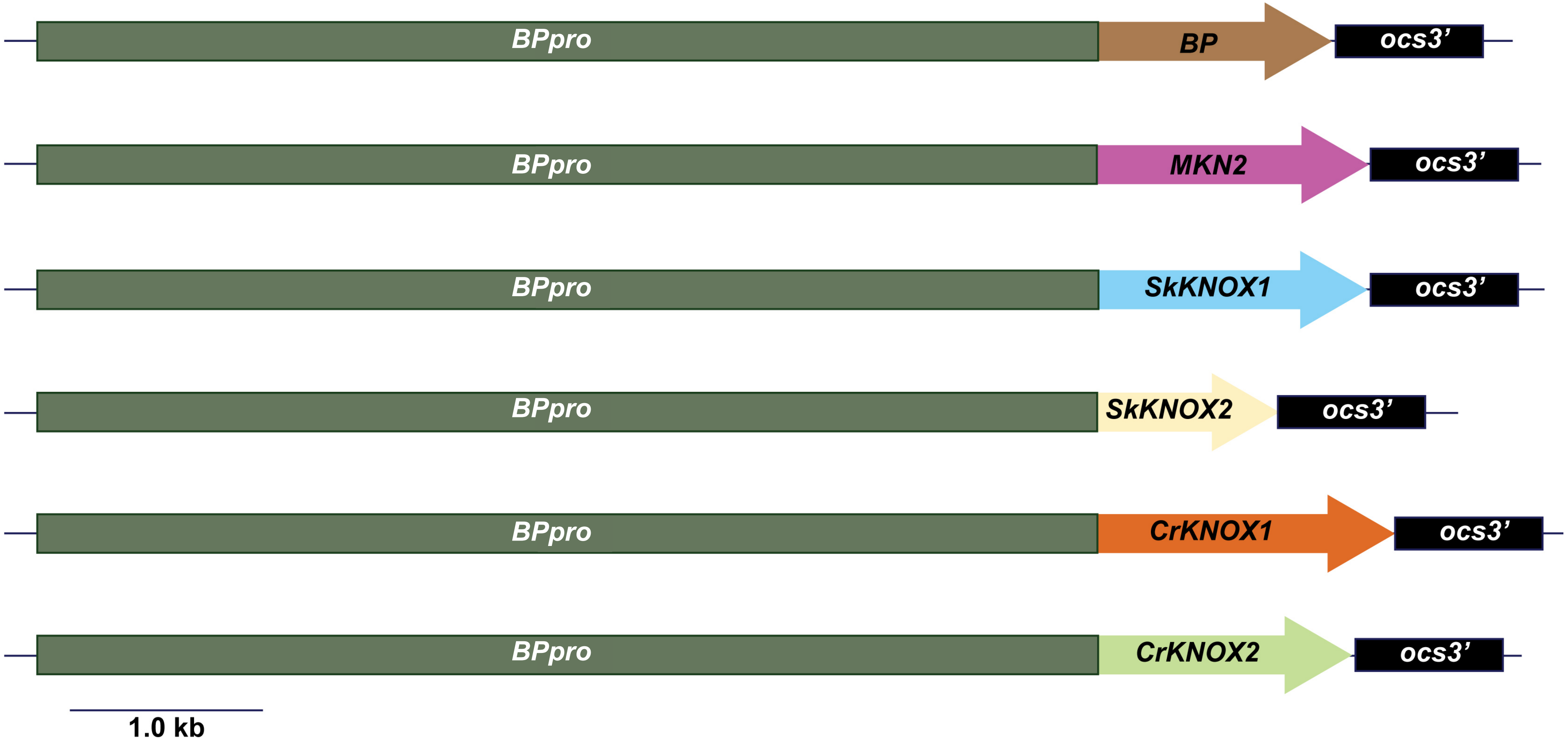

Supporting Figure S4.
KNOX maximum-likelihood phylogeny including *Klebsormidium* sequences.

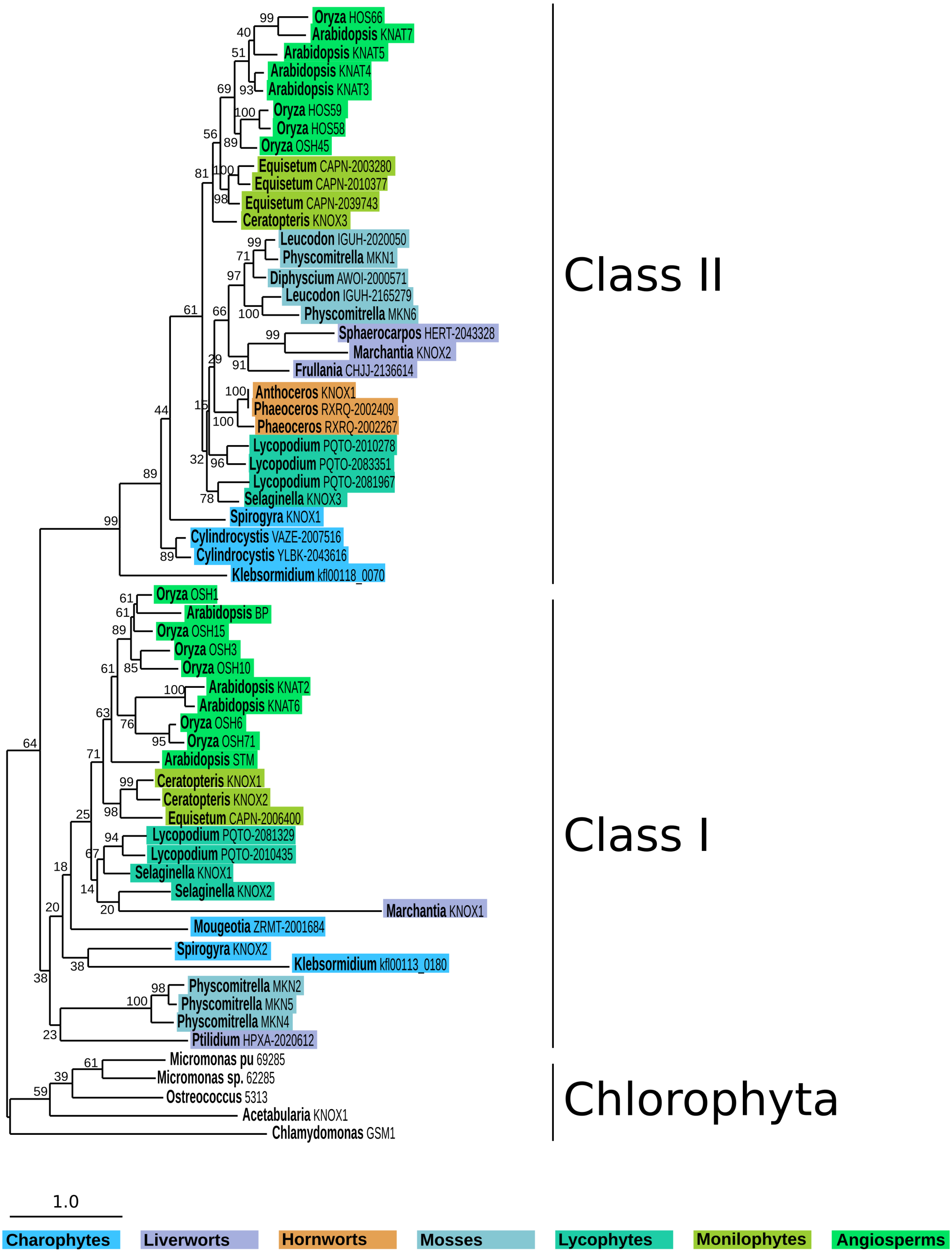

Maximum-likelihood phylogeny of KNOX homologs inferred with RAxML using a partitioned alignment as described in Materials and Methods section and the GTR+G+I evolutionary model. Bootstrap values obtained over 100 bootstrap replicates are shown next to the corresponding branch. Colour coded boxes indicate species' phyla. The tree was rooted using the Chlorophyta clade.

Supporting Figure S5. Predicted secondary structure of the MEINOX domain.

a) BP **b)** CrKNOX1 **c)** CrKNOX2 **d)** SkKNOX1 **e)** SkKNOX2 **f)** MKN2. Main panel shows confidence level in components of the model (red is highest confidence, blue is no confidence). Inset shows N to C terminus of the same region on a rainbow scale with blue as N terminus and red as C terminus. Predictions inferred using Phyre2 (http://www.sbg.bio.ic.ac.uk/phyre2/html/page.cgi?id=index) using the 'intensive' setting and inputting peptide sequence from the start of the KNOX1 component of the MEINOX domain to the start of the ELK domain in each case.

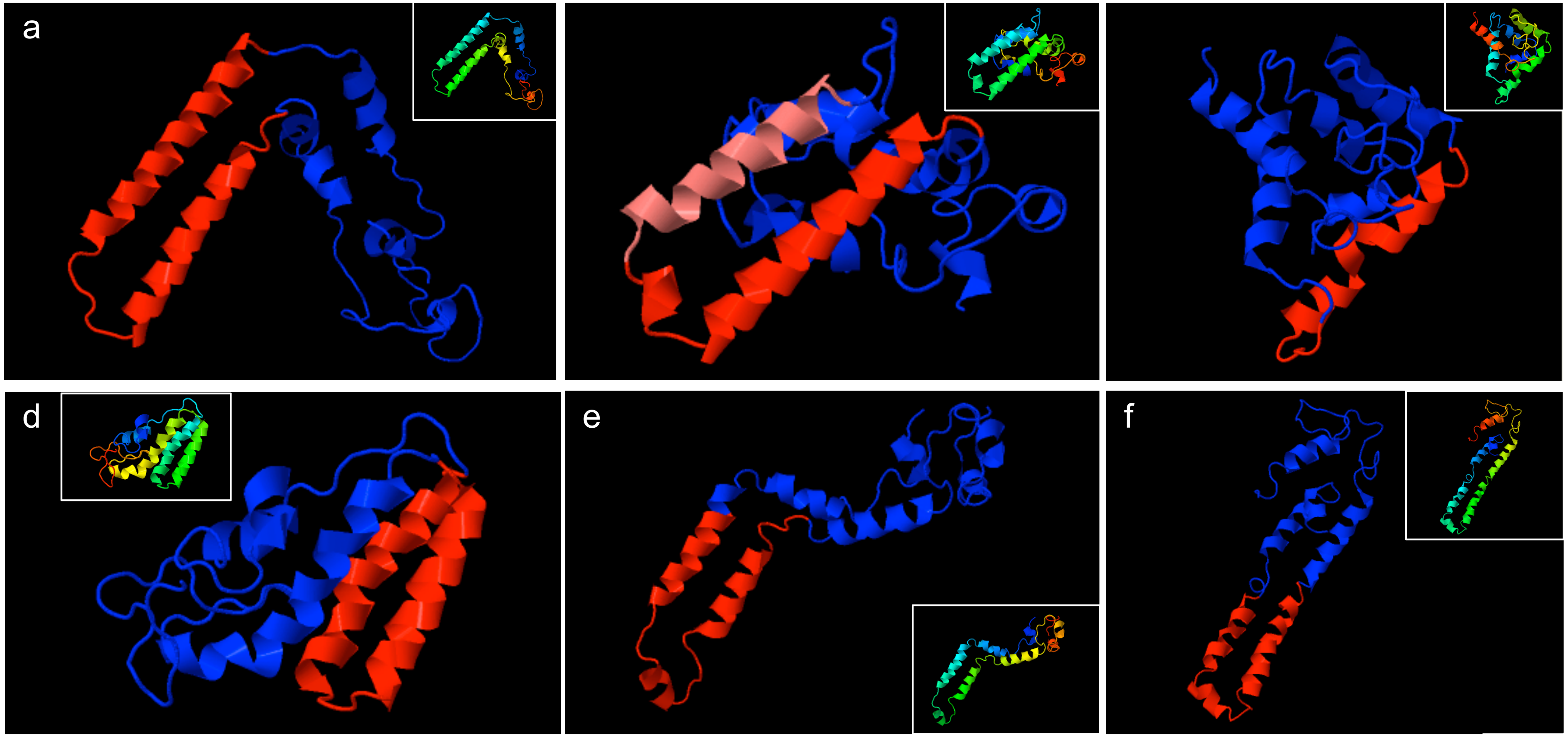

Supplement: Supplementary file 1 — Fig. S1 Graphical representation of KNOX alignment showing KNOX conserved domains and partitioning used for inferring phylogeny. Fig. S2 Construct design for homologous recombination of KNOX genes in the Physcomitrella patens MKN2 locus. Fig. S3 Schematic of BPpro:KNOX constructs for bp complementation experiments. Fig. S4 KNOX maximum‐likelihood phylogeny including Klebsormidium sequences. Fig. S5 Predicted secondary structure of the MEINOX domain. [file NPH-216-591-s001.pdf]
