# Supplementary material for: Nonreciprocal complementation of KNOX gene function in land plants
Source: New Phytol. 2016 Nov 25;216(2):591–604. doi: 10.1111/nph.14318 (PMC5637896; doi:10.1111/nph.14318)
Supplement: Supplementary file 2 — Methods S1 Supplementary details for methods. [file NPH-216-591-s002.pdf]

Supporting Information Methods S1 Supplementary details for methods.

Plant strains and growth conditions

Marchantia polymorpha male (*Takaragaike-1*) and female (*Takaragaike-2*) plants (Ishizaki *et al*., 2008) were grown on soil-based compost (Levingtons M2) as in Saint-Marcoux *et al.* (2015a) *Spirogyra pratensis* (UTEX strain LB928) was grown in 1X or 0.1X Bold's medium (Sigma #B5282) adjusted to pH 7.0 and supplemented with 1X Kao and Michayluk vitamin solution (Sigma #K3129) under a 16 h light and 8 h dark photoperiod at 22°C and a light intensity of 20-30 $\mu Em^{-2} s^{-1}$. The Oxford strain of *Anthoceros agrestis* was grown and maintained as in Szövényi *et al.* (2015). *Selaginella kraussiana* (Kunze) A. Braun was obtained from the Royal Botanic Gardens at Kew, UK and was grown on peat-based soil at 23°C under continuous light. *Ceratopteris richardii* was grown and maintained as in Plackett *et al*. (2014).

Isolation of KNOX gene sequences

DNA was extracted from *Physcomitrella patens* and Arabidopsis according to Yasumura *et al*. (2005).

RNA was extracted from *M. polymorpha* and *S. pratensis* using the Arcturus PicoPure RNA extraction kit (Life Technologies #KIT0204) from tissues that had been harvested by laser capture microdissection (LCM). Prior to LCM, *M. polymorpha* tissues were fixed in 100% acetone, paraffin embedded (Paramat, VWR #361148C) and sectioned to 10 µm slices; *S. pratensis* filaments were fixed in 25% ethanol / 10% acetic acid / 1X RNase inhibitor (Sigma #R7397). LCM was performed essentially as in Saint-Marcoux *et al.* (2015b) on a Carl Zeiss PALM MicroBeam. Amplified cDNA was obtained from extracted RNA using the Ovation RNA-Seq system v2 amplification kit (NuGEN #7102) as described in Saint-Marcoux *et al*. (2015b)

Total RNA was extracted from *P. patens, S. kraussiana, C. richardii* and Arabidopsis according to Yasumura *et al.* (2005) and from *A. agrestis* according to Szövényi *et al*. (2015). cDNA was synthesized using Superscript III reverse transcriptase (Invitrogen, Life Technologies, USA) and RT-PCR was carried out using primers listed in Notes S1.

MKN2 5' flanking region and *MKN2* 3' flanking region were amplified from *P. patens* genomic DNA; *AagKNOX1* was amplified from sporophyte cDNA; *SkKNOX1* and *SkKNOX2* gene coding sequences were amplified from *S. kraussiana* sporophyte cDNA; *CrKNOX1* and *CrKNOX2* gene coding sequences were amplified from *C. richardii* sporophyte cDNA; *BP* and *STM* coding sequences were amplified from Arabidopsis 2-wk-old seedling cDNA; the promoter of *BP* was amplified from Arabidopsis (*Ler*) genomic DNA; and the *nopaline synthase* (*nos*) terminator was amplified from pBRACT207 plasmid (BRACT, John Innes Centre Norwich Research Park Norwich, UK).

Isolation of *SkBEL* gene sequences

DNA was extracted from *S. kraussiana* using hexadecyltrimethylammonium bromide (CTAB). 350 mg of ground frozen *S. kraussiana* tissue was added to 3 ml of CTAB extraction buffer (100 mM Tris-HCl pH 8, 1.4 M NaCl, 20 mM EDTA pH 8, 2% (w/v) CTAB, 0.3% (v/v) β- mercaptoethanol, 1 mg ml^{-1} RNaseA) and then incubated at 65°C for 30 min before chloroform extraction and isopropanol precipitation. *SkBEL1* and *SkBEL2* sequences were obtained first by amplifying the TALE-HD from genomic DNA using degenerate primers (Notes S1). Full-length *SkBEL1* was then obtained by inverse PCR using an XbaI or EcoRV restriction digest and primers complementary to the TALE-HD region. *SkBEL2* was obtained following three consecutive rounds of inverse PCR firstly using BamHI, and subsequently XhoI and KpnI. To prepare template for inverse PCR, 1 µg genomic DNA was first digested with specific restriction enzymes (*SkBEL1* – XbaI or EcoRV; *SkBEL2* – BamHI, XhoI or KpnI; NEB). After ethanol precipitation, 500 ng digested DNA was self-ligated using T4 DNA ligase according to the manufacturer's instructions (Promega) and then re-precipitated with ethanol. One hundred to 150 ng of genomic DNA or 5–10 ng of plasmid DNA was used as a template in PCR reactions using Phusion proof reading polymerase enzyme mix (Thermo Fisher, UK) and primers as listed in Notes S1.

SkBEL1 coding sequence plus the stop codon and 3'UTR region of *SkBEL2* were amplified from sporophyte cDNA by 3'RACE PCR. Total RNA was extracted from *S. kraussiana* according to Yasumura *et al.* (2005). 2 µg total RNA and 0.5 µl oligodT anchor primer (10 µm) were heated at 70°C for 10 min, placed onto ice and then added to cDNA synthesis mix (5 µl 5 × RT buffer, 2 µl 0.1 M DTT, 1 µl 10 mM dNTPs, 0.25 µl RNasin (Promega). 1 µl Superscript III reverse transcriptase was then added and incubated under the following conditions: 18°C for 5 min, 50°C for 90 min, 70°C for 15 min. The resulting cDNA template was then used in a PCR reaction using the 3'RACE anchor primer and *SkBEL1*/*SkBEL2* gene-specific primers (Notes S1). The *SkBEL2* coding sequence was predicted by aligning the *SkBEL2* genomic DNA sequence with the *SkBEL1* gene coding sequence.

Phylogenetic analysis

Evolutionary models were tested using jModelTest2 v2.1.7 (Guindon & Gascuel, 2003; Darriba *et al.*, 2012) for the nucleotide alignment and ProtTest3 v3.4 (Guindon and Gascuel, 2003; Darriba *et al.*, 2011) for the protein alignment, and selected upon the Akaike information criterion (AIC), corrected AIC, Bayesian information criterion and the decision theory method. Consequently, KNOX and BELL sequences were allowed to evolve under GTR + Γ + I and JTT + Γ + I models, respectively. The KNOX nucleotide alignment was partitioned by grouping the MEINOX and ELK domains in one part (alignment site 1–405) and the HD-TALE domain in a second part (alignment site 406–597) (Fig. S1). This partitioned dataset was found to improve support values and overall tree topology during

exploratory work. Maximum-likelihood trees were obtained with RAxML v8.2.3 (Stamatakis, 2014) using the rapid hill-climbing algorithm and 1000 bootstrap analysis in one run. Bayesian trees were obtained using MrBayes v3.2.5 (Ronquist & Huelsenbeck, 2003) and two parallel runs of three hot chains and one cold chain. The nucleotide tree was run for 3000000 generations at which point the average standard deviation of split frequencies was below 0.015. Convergence of the two runs was also assessed by examining the potential scale reduction factor (PSRF) statistics reported by MrBayes: all were very close to 1 for every parameter at the end of the run, indicating convergence of the two trees. Similarly, the protein tree was run for 300000 generations, at which point the average SD of split frequencies was below 0.008 and all PSRF parameters were close to 1. Placement of sequences after phylogenetic reconstruction was achieved on maximum-likelihood trees using the evolutionary placement algorithm of RAxML (Berger *et al*., 2011).

During exploratory work for both phylogenies, a KNOX and a BELL *Phaeoceros carolinianus* sequence that was retrieved from the OneKP dataset was placed in the angiosperm group with high support values. Because OneKP samples are prone to contamination, as stated by the project website (see also Notes S4), both sequences were excluded from the final datasets.

Statistical analysis of silique angle measurements

Measurements were performed on 7-wk-old plants. Silique angles on the main inflorescence were measured for 10 plants per line using a protractor. Silique number per inflorescence ranged from seven to 36, with a total of 4849 measurements recorded (Notes S7). The dataset was transformed into a normal distribution by calculating reciprocal values for each measured angle. One way ANOVA and Tukey's Honest Significant Distribution (HSD) tests were carried out using R (Team, 2015). Silique angles in all transgenic lines were significantly different ($P < 0.05$) from nontransformed mutant *bp* lines (Notes S7). With the exception of two *BPpro:SkKNOX1* lines (lines 1 & 3), four *BPpro:CrKNOX1* lines (lines 1, 2, 3, & 5) and all five *BPpro:CrKNOX2* lines, transgenic lines were not significantly different ($P < 0.05$) from wild-type plants or from fully complemented transgenic *BPpro:BP* lines. To check whether silique number per inflorescence and/or position on the inflorescence influenced the observed variance, the dataset was trimmed to include only inflorescences that had at least 10 siliques, and only the first 10 siliques from the bottom of each inflorescence. The resultant 2912 measurements were transformed as for the complete dataset but in this case, *BPpro:BP* line 5 and *BPpro:SkKNOX2* line 3 datasets remained non-normal. One way ANOVA and Tukey's HSD tests revealed almost identical results except that only three *BPpro:CrKNOX1* lines (1, 3, & 5) were significantly different from wild-type instead of four (Notes S7).

References

Berger, S.A., Krompass, D., and Stamatakis, A. (2011). Performance, accuracy, and web server for

evolutionary placement of short sequence reads under Maximum Likelihood. Syst. Biol. **60**: 291–302.

Darriba, D., Taboada, G.L., Doallo, R., and Posada, D. (2012). jModelTest 2: more models, new heuristics and parallel computing. Nat. Methods **9**: 772.

Darriba, D., Taboada, G.L., Doallo, R., and Posada, D. (2011). ProtTest 3: fast selection of best-fit models of protein evolution. Bioinformatics **27**: 1164–1165.

Guindon, S. and Gascuel, O. (2003). A simple, fast, and accurate algorithm to estimate large phylogenies by Maximum Likelihood. Syst. Biol. **52**: 696–704.

Ishizaki, K., Chiyoda, S., Yamato, K.T., and Kohchi, T. (2008). Agrobacterium-mediated transformation of the haploid liverwort *Marchantia polymorpha L*., an emerging model for plant biology. Plant Cell Physiol. **49**: 1084–1091.

Plackett, A.R., Huang, L., Sanders, H.L., and Langdale, J.A. (2014). High-efficiency stable transformation of the model fern species *Ceratopteris richardii* via microparticle bombardment. Plant Physiol. **165**: 3–14.

Ronquist, F. and Huelsenbeck, J.P. (2003). MrBayes 3: Bayesian phylogenetic inference under mixed models. Bioinformatics **19**: 1572–1574.

Saint-Marcoux, D., Proust, H., Dolan, L., and Langdale, J.A. (2015a). Identification of reference genes for real-time quantitative PCR experiments in the liverwort *Marchantia polymorpha*. PLoS One **10**: e0118678.

Saint-Marcoux, D., Billoud, B., Langdale, J.A., and Charrier, B. (2015b). Laser capture microdissection in *Ectocarpus siliculosus*: the pathway to cell-specific transcriptomics in brown algae. Front. Plant Sci. **6**: 54.

Stamatakis, A. (2014). RAxML Version 8: A tool for phylogenetic analysis and post-analysis of large phylogenies. Bioinformatics **30**: 1312–1313.

Szövényi, P., Frangedakis, E., Ricca, M., Quandt, D., Wicke, S., and Langdale, J.A. (2015). Establishment of *Anthoceros agrestis* as a model species for studying the biology of hornworts. BMC Plant Biol. **15**: 98.

Team, R. (2015). R Development Core Team. R A Lang. Environ. Stat. Comput. **55**: 275–286.

Yasumura, Y., Moylan, E.C., and Langdale, J.A. (2005). A conserved transcription factor mediates nuclear control of organelle biogenesis in anciently diverged land plants. Plant Cell **17**: 1894–1907.
