# Supplementary material for: Nonreciprocal complementation of KNOX gene function in land plants
Source: New Phytol. 2016 Nov 25;216(2):591–604. doi: 10.1111/nph.14318 (PMC5637896; doi:10.1111/nph.14318)
Supplement: Supplementary file 4 — Notes S2 Verified Marchantia polymorpha, Spirogyra pratensis and Anthoceros agrestis KNOX plus Selaginella kraussiana BELL sequences. [file NPH-216-591-s004.pdf]

New Phytologist

Supporting Information Notes S2 Verified *M. polymorpha*, *S. pratensis* and *A. agrestis* KNOX plus *S. kraussiana* BELL sequences. Coding sequences are shown in bold typeface; 3'UTR sequences where known are shown in blue and homeodomain sequences in red; start/stop codons where known are underlined; sequences confirmed by PCR and sequencing are highlighted in yellow.

M. polymorpha KNOX

>AXG93_606s1230|MpKNOX1 cDNA
GTACGATGAATTAAAATTCCACTTTTCTGGACTGTAGCAGTGGTCAGCATTCAGGCGCT
CGTCCTGGTTGCGAAACTTCAGTATTGCATCTCGTTATAGAATCGAGAGTCCGGGATTG
GAATTGAAACCTACGCATTTGTCACGGTTTCCTGGAAA**ATGCTAGGCAGCAGTTCCTTC**
TTTGACTTCAATTTTGATCAGGAAGTTGCCAACCTACATCTCTCCGACTATTGTCAAAC
TGAACCACAAACGCCACTGATAAACACACATACCACGCTATTCACACAAGGAAGTAG
TCCTAACTATCAGAGTCAGGTCTCCGTTGATCTTCAAACGGAATCATTCGAGCTCTCT
CCTCGAGGTCTCTCCACGATGACTATATCAGAAGACACGGGTTCAGAATACCACGCA
GCCACTACAGAGCCTTTTCTCTACCTGTCAGACTTGGAGACACCACTTCAGAACACAG
CTGATGGTAAACAGGATGTGACCCTCTCGGTTAACCTCACGGCATCTGATACGACAG
TTCGTGCCGAAATTCAGGAAAGTGGAGAAAAACCACCATGTAATTCTGGTCATTCTTT
GTTGGACTCAATCTCTTACAATTCTTCGAGAGAAGCTGAAAACGATATAGAGTTTTGG
CTACGTGCATATACAGCTCCAAGCAAAAATCTGCCTTTCGGCTGTGGGGAACCTAAG
AAAACATACCACAAAATAGCAGACGGTAATTGCAACGTAGTCGATCAGAAGCCATGG
GGCCCGCAACTGCTGCAATCATCTGAAGCTCCAAATCACGTCCTATCTCTCGAGACA
AGAAAAGATGGAAATTCGAGTCAAGTAATTCTGGAAGGCCAATCTAACCTTGTGGAA
GTGCAAACTGCGACTCGACGCTCGCCACGTTCACTTGAAGCTCTAAAAGATCTTGGG
ATTTTTATGTTTGGAGAAACTGAAGAGCCATGTCCCACCGTTGCAGAGGGTAAACTCA
ACATTGAAAATACAGATACACTTTGGCAGCCTCAGTCTGAACCACACGATGACACTCT
CTTTGGAGAATTGTTTAACTTCGACGATCGCGCAGAGGATGAAGATCACCGTCAATAC
GTTTTGGGATCCATGTTGGAGGAGCTCGAGAAGTTCGTTGACAAATCCGATAATGAAC
TTTGTAGAAGTTCTGAGACAACAAATGAATTAGACCTTCAGTGTCTCATCGTGCATCA
TCCTGACCTCCCTAAGATAATTGTGGCAAGCCTTGCCTGCTATAAGATAAGGGCTGAT
GAAGAGGAGAAAAAGCAGCTGGACGAGGTGGCCAACTTATCTCTTTCCAAGCTCCCA
GCACAAGACCAGCTTTTGGGGCGTGGATACTCCAGCAAGGGTAAAGACCCACGTCTC
GACTTATGCTTGGATAAGTATCTGAAAGCCCTCGATGAGTTGGAGAAACACATTTCGA
AGCTGGTCACGTCAGCCGACACTCACTGTGTTCAGATGAAACAGAACATTCTTATACA
GTGTCCAGAACTTGGTGATGTTTCAAAGCAGAGCGAAAGTGATTCGGTCAGTGGTGG
GCAAAGTGAGCTTTTCACTGAAGAAATGGAGAAGATAATTCAAGACCATGAAAAAGC
AAAAGACAGGCCCCAGCGATCTGCCCTCCCCATGTTGACAGATCAAGAATGGAGAG
GAGCAATCTTAGCTGCATATAATCCAAGTCTTCTCAGGACCTTGAAAACGAGGCATCT
GAAAGAAGGCCGCAGAAACCTGAAGCTGCCCGATCCGGCTCGACGTATTTTGCTCAA
TTGGTGGACCGAACATCAGGGGAAGCCTTACCCGTCGGAGGAGAAACAAATTCTGGC
CAAGTGCTCGGGCCTTGAGATTGATCAGATCAATAACTGGTTCATCAATCAGAGGAA
AAGACACTGCTGCTCTGCGTTCTCTGAAAATCACAAGAACCATGCGGCCGACTGTTA
CAAGATGAATGGCCCAAGACGATGATCTCCAGCCCCTTTTCGTTCGCAGCTAGATTGA
CATGCCTTCAGGAGAATCTATGATGTCGGACTCGATGACTCAAATCACATTTCTGCCAT
GGACGTTTCTCAGAAGACGAGATTTGGAAGCTGTGAGCTGTACGTTGTGAGAACCAAAT
GGAATCTTAACCATGATGTCATATAGAAGTCCAATAACTTTGAGGCCAGGTAGCTCATTC
TGAGTTTGTCTGCCCAGATACATCCTGCAGATATTATGTCCCTTAGACTACGATTCTGAA
A

New Phytologist

>AXG93_3769s1000|MpKNOX2_cDNA_long_version
ATGGATGTGTCTGTGCACAGCGAAATGAGGAGAAGTAGCGGATCTCAATCGCTGAATC
TGTTGCCACCCTCACTGTCAGAAGGTTGTGATCGAGACATCGAGGTCGTGTCGTCTCT
GCGCCCATGTTTCGACAGTAACGCAGACGCCAAGCCGTACCATATCGAGGGAGATCAC
GATCCGCTGCTGGAATCTCAGCCGCGCAATGAAGTCGAGGTAGAACGGTATTCGGGCG
AGGAAGATCACAATCCGCTGCTGGATTTTCATCAGCGGAACCACGAGATTGGGAGTCT
CCTGCTCCATGAGTGCAGAGAGCAACAGACCGAGGAGCATGGGAGTCCGATGCTGCT
CTGTGCGGAGGGGAGTGAGGTCCAACCATTTCCTCAGGGATTTGAAGAAATGCAAATC
GACGACAAGGAAGAGGAGCGGGAGCAGACTAGGTTACAGCATACGCAGCTCGATGGA
GATGGTGATCTGAGAGAACTGTCAGCTCCGCATGATTTCGAGGGGACGGCGAGCCGG
ATGCAGCTGGAGACCTTCCACCAGCCTTCCTATGGTAGTGGGGATGGATCCCACGGAG
AGGAGGCCCTTCAGCTTGTGTATTATGAGAATGGACACGAGTCTCAGAGTGTCATGGAA
TATGCTCACCTGCAGGAGCAAGAATTGGCATTGCACCAATATGCCATCGCGAACCAAG
GTCGAGCTAATCATTCTACGTGCGAGATTGAGAATGAAGATGACACACTACATGAACAT
CTTCCAGAACACTGGACTGGATCTAGTTTGCACTCGACTGTGGCTGAGACGAAGGGAG
CAGAGTACGGTTCTGAAAATGGGATGTTGTTTAATCACCTGCATGATCAACAGATGGCA
CTGCAGCAATATGCCATCCAGAATCCAAACGATGTGATTCTTCAGACTCGTGAGGATGA
GAATGAAGGTCACAGATTACATGGAAGTATCCGCGAACACTTTACTGGATCGAGAATGC
ATTCAGAATTCAATGTGCCGGCGGAGAGGAAAGGAAATTTCAGTGAGCGTCAGGGACT
TTCCACTCAGGTGATGCAAATTGATGATCAAAGGGAATCAGATCTCACTGGCGGGGATA
CCCAAGGCCTGCAGGATATGGGAGGATGGATGGATACGAAGGCCTATCCCTGTGATGA
GCAGTACCATGTAAGCATAGGAACAGGTTTCGTGGGAGATGTGCATGGCCAGGCTGAG
ACTAGCTCTCTTTCAGCGATCGAACGAGAGGTTTCTTTTTCTGCACAATTGGCGGAAGA
TGTTGTCATGAGCTCAGAAGAGGCACAGGCACATCAAGGAGTTTGGAAGTCTCTTCCTC
CCAGATTTAATCAATTCGGTTCTTGGATGCCAGAGAGGGGAATCTCCAGACAATTTACT
ACAGCACCACAGTCAAATGCCTCATCCTACACTGGTGGATCGTCTCCAGTAACAGGGCT
TGTGACACCAAGATCAATTGCAAGTCCCACAAGGATTCCGCAAATGGACGTCAATCAAG
CTTCCTCACAACTGATAATGGGATCTACAATGGCGTCTCAATGGATGGGTGTTTCTTCTT
ATACTCCTAACGACTTTGACTATGGGGTGTCTCAGATGAAGCTTACCCAGTCTTCAATC
GAACATGCGGAATGTGGAATGAGTACTCTAAGGAGCCCTTCAGAACTTCCAGTCAGTGT
AAATTCAGCTGTAGATTTCAGCAACCAATCTCAAAGTACCCAGATGCCAATTGACTTGAA
AGCAATGCAAACATTTTGCGCAGGCAACAGATTCGAAGATTTTGTACCCATACAGGAAG
GTGCGAAGGTGGGTAATATGTATCTACCATCATTTCATTCACCAGCCATAGTTCCTCATC
TGAATGTCCAGTCACCGTCAATTGCAGAGTTAGCTCCAGTACCTTTTACCCATGCTCAC
TTACTATCTTCTGCACCCATGAATGATGTGCCTCGGTATTCCAACTTAGAACATCCAAAC
CAATTCCAGGACATAGGTCAGCTTGCAGATAACCATTCCTTGCAGGATTCGAATTCTGT
TCCAACCTTATTGCAAAATATCAGTGCAGAAACAATTCAAGCAGCAGCCTTGGGCTTAA
ATTGGCCTAGAGGGTCTCCAGTGATTCAGAATGCAAGCGATCAATTCAACTCTTATTCC
CTGCTTCAAGGCTCCGAAACTGTTGGACCTGTTTGGAATTCTTTACCAGTGATGTCAATA
GATAATTCCACGGTTTCTCCAGCTGAACCAACTTGGAACGCGACATATCCCCATGGAGC
TCATGTGAATCAGGATCAGGTGCAGTTTCCGCAAACTTTGTCAGTGGATCAGGCAGAAA
GTTATGCTAGATCTGCTCTTGCTTATGGTCAAGAAGCAATCGGAATTAGGGATCTAGGT
AGCCATGATCATAAAAGCACAAACAAAGAGCTACCAAGAGCAGCGACTGGAAATATTCA
AAGCGTTGCGGAACAGCTTGGAGAGCAAGAGTCATTCCAACACACTGGA**ATGGGGAGT**
GTATCCGCCAACGACAGCCATATGGTGCAGGGAGACCATGTGCAGCAAGTGGAGGC
AGGAGCATGGGGAATCATGCCCAGTGAGAATGATGGTGTGGTCAGAAGTGAGGAGG
TCAATCAGCTTGGCAGCGGTGTATATGTTAACCAAGAGGAAATGTGTTTGGATGGATA
CCAAAACCATTGTGGGGATTTGCAGATACAGGAATTGACTGGCCTGTCAGCAGATGA
AATGAATTATCTGGGGTACTGTGAGAGTCAGATTGAGAGCGTAAGAAACCAGGCAGG
AGACATGCTCATGACTAATCCGGTGGATGGAAAGCAGCCTGAATTATCACAATCCGG
GGAAGGACAGCCAGGAAAAGGGGCTCCAGAGCCTCAGGTCTTATCTCAGGAGATGG
AAAGGTTGTTATCTGACAGAGATCGATTGTCAACGAAGCTCCTTTTCTGGAAGAGGGC

CAGATTCAGGGCTAGACTCATTACCCATCCATTATTTCCTCAACTCCTCAAGGCACAT
GTAGACTGCATCCGTGTGATGACTCCAGCATCCAAGATACGTGACATCAATCTGCAG
CTGGTTCAGGCTCAAAAGTTTTTCGAATTTTTCCTCCCGTTTGCGCCCAAATGCCCCCA
GCCTGATGATTTTGAATTCGACTACTTCCTGGAAAATTTCCTGGTTCGGATGAAACATT
TCCAATCGAAATTGGAGATGCAGCTCAAGGTTCACACCACTTTGGCTTACAGCAAATT
ATGGCGCATCGAGCGGGTACTCGTCGACATGACAGGTCAGCGCATTCCCAGTCGGTT
GGATGATGGGCCTCTTATGTCTGACAACCCAGACAATAAAATTGGACTTCGGAAGGC
TGTTGAAAGAGATGTTGCAAGTGCTGTAGAAGAACGCTTGCGCGAGGATAATGGCAG
TTCTAGCACTGACGATGACGATCTCAGTGACTCAGACCTCGATACTGATATCCAGAAC
GCCGATGACCCTGATCTCCCATGTGCAGACAATCCAAGTGAACAGTATAGCGATGAT
GCTAATGTCGAGTTTATGGATGATCCTAGTCTCCAGAATGCCGGTGGTGCTAATGTCC
AGTATGAGGATGATCCTCGCCTCCAAAACGCCGGTGgtgctattttccagtatgggaatgatcctag
tttccaaagtgccggtgttcctgatggccagtatgagcatgatcctagtctccagaatgcaggtggtcctgatgtccagc
atggggatgatcctagtctccagaatgccggtggttctgatgtccagtatgaccatgatcctagtctccagaatgccggt
ggtggtattatccagtacgggggtgatcctagtctccagaatgccggtggtgctgatgtccagtatgaccatgatcatag
tctacaaaatgccagtggtgctattgtccagtatgggggtgatcctagtctccagaatgctggtggtcctgatgtccagta
tgagcatgatcctagtctccagaattccggtggtgctattatccagtatggggatcgtactagtctcgagaatactggtgg
tgctaatgtccaatatgtggatttttccagtctccagtgtgctgctggtgccagtttccagtatggggattatcctagtctcc
agaatgccggtggtgctaattttcaatgcgtggatgatcctagtctccagtatgctggtggtgctaacgtccagtatactg
ataatccgaatatccagtataccggtgatccgaatgtgcagtttgttgatgatgcgaatctccaatatggcggtgattcta
gcgtccagtacactggaaatccgaatttccagtataccggtgatccgaatgtacagtttgtagacgatcatactttccagt
ttggagGTGATTTTAATCTCCAGTTCGCTGATGATCAGAATCTCCAGTATACAGGTGATC
CGAATCTACCGTTTGCTGATGACCCGAACGAACAGTATGGGGATGATCCTAATGTCC
AGTATGTCGATGATCCTGATGTCCAGTCTGCTGATGACCCAAATTCTCAGTGTGCTGA
TGATGCTGATGATGTTGAGCATGCTGGTGATCCGGATGTCCAGAGCGCAATGGACTC
GGACTATTTTGGAACTACTGTTCCAGATCGTGCTGTCATGGAGAGCATCAGGCGAGA
GTTGATTGCATCACTTGGTTTTGAAAAGTACAAGAAGAAGATCAAAGAAGTGAGAGAT
GAGATTTTGAGAAAACGAAGAGCGGGCAAATTGCCAGACGGAACCACACAAATACTC
AAGGCGTGGTGGGATGCTCATTCAAAGTGGCCTTATCCAACGGAAGATGAGAAAGAA
GTTCTTCTCAAAACGACGGGATTGGAACTGAAACAAATCAACAACTGGTTCATAAATC
AAAGGAAAAGAAATTGGCACAACAATCCACTGTCATCGAGCAATAAGGAGAAGCGG
AAAGTAACTGTGACAGATGTGATGCAAGAGTAGATAACCATCCAGTCACACTAACAGT
TA

>AXG93_3769s1000|MpKNOX2_cDNA_long_version_translation
MGSVSANDSHMVQGDHVQQVEAGAWGIMPSENDGVVRSEEVNQLGSGVYVNQEEMCLD
GYQNHCGDLQIQELTGLSADEMNYLGYCESQIESVRNQAGDMLMTNPVDGKQPELSQSG
EGQPGKGAPEPQVLSQEMERLLSDRDRLSTKLLFWKRARFRARLITHPLFPQLLKAHVDCI
RVMTPASKIRDINLQLVQAQKFFEFFLPFAPKCPQPDDFEFDYFLENFLVRMKHFQSKLEMQ
LKVHTTLAYSKLWRIERVLVDMTGQRIPSRLDDGPLMSDNPDNKIGLRKAVERDVASAVEE
RLREDNGSSSTDDDDLSDSDLDTDIQNADDPDLPCADNPSEQYSDDANVEFMDDPSLQNA
GGANVQYEDDPRLQNAGGaifqygndpsfqsagvpdgqyehdpslqnaggpdvqhgddpslqnaggsdvqyd
hdpslqnagggiiqyggdpslqnaggadvqydhdhslqnasgaivqyggdpslqnaggpdvqyehdpslqnsggaiiqygd
rtslentgganvqyvdfsslqcaagasfqygdypslqnagganfqcvddpslqyagganvqytdnpniqytgdpnvqfvddan
lqyggdssvqytgnpnfqytgdpnvqfvddhtfqfgGDFNLQFADDQNLQYTGDPNLPFADDPNEQYGDD
PNVQYVDDPDVQSADDPNSQCADDADDVEHAGDPDVQSAMDSDYFGTTVPDRAVMESIR
RELIASLGFEKYKKKIKEVRDEILRKRRAGKLPDGTTQILKAWWDAHSKWPYPTEDEKEVLL
KTTGLELKQINNWFINQRKRNWHNNPLSSSNKEKRKVTVTDVMQE

***S. pratensis* KNOX**

>SpKNOX1|cDNA
**ATAGGAAGCCTGGATAATATTAGTCGAGTTTCTAATCCAAATAATATCTTGCTGAATTT
AACAGAGGATAAATTCAATGATGCTCTTTTGAGTAATGGCATACCTTCTAATCTTCCGC
AAACCATAGATAACTTGAGCCATGACTATAAATTTCATGAACTCAACAACTTTTCTTCA
ATCCAACACTTCTGTTCGATGAATGAGCATACCGCTCAAGACTCTCGGCTTGAATTTT
CATGTCCGACCAGCTTAATGAATGACGAATTGCAAAACGTCGAGCGTGCTCTTTCTTG
TAGAAATGATTCTACGTGTTCGTCAAGTTTGCAGAATTTAGAGACGGGTTATCTTCCC
AAACTAAGAACGGGTACTTACGCAGCTCAAGAAGAAACCACATTGACTGCTTTCGAA
AATGGCTTAAATAATGAACCTAATGGTTTGGTTTATAATTTCGATTACGGAGCTAACCA
AAACATTTATCAAGGTTCGGCGGAGGTAGAAAACACCGAGGCTTCACATTTAGCAAA
CGCTTACCCAACTGATTTCCACGTTTTTCAAAACGTAAACCAGACCTCACGTTCGGGT
GATGGCAATAATGGTTTTGAAAGACCCGAGCCTTTGGCAGCGCAGAAAGTTGATGAA
GATGAAAAGATCAATGCTATCAAAGCTGAAGTTGTTTCTCATCCTCTGTTTAATCAGCT
ACTCGAGGCTCATGTGGCGTGTCTTAAGGTTGCAACGCCTCTCGATCAAATCCCCTTG
CTAGACCTCCAATTAGAAAAGAAAAATCTCGTGATTGAAAAATATGAAAAATTTCTCA
AGGACCCGAAGTATGGAAATTTTAAAAACAAAATCGAAGAAACAACCGAGCTTAATT
TTTTTATGACGCATTTCGTGTGTTTACTTCAATCTTTCAAAAGCCAACTAGAGCAACAT
GTATTGATTCAAGCCAGAGAAGCAGTAATGTCATGTTGGGAATTAGAACAATCCCTCA
TTTCGTTAACAGGTATGGAGCCTCGACCTGGTACAGGATTAACCATGTCTGACGATGA
TGATGACGACTTGGACGACGAGGATACTATTGATTATGATAAGCATGCTTCAGAAACT
CTAACAGACGGAAATCAAGATCCTTATGGACCACTTACACCATCTGAAAACGAACGC
AGCATTATGGAACGTATGAGGACGGAACTTAAAAAACAGTTATCAGATCAAGGCTAT
CGTACCAAAATAGCAGACGTGCGTGAGGAAATTATGAGAAAAAGAAGGGCAGGAAA
GTTGCCTAGCGATACGACCCAAATTTTAAAGAGTTGGTGGAATGCCCACTCAAAATG
GCCGTATCCTACAGAAGACGAAAAGACTACTCTGGTTCGGGAAACCGGTTTGGAACT
GAAGCAGATAAACAACTGGTTTATAAACCAGAGGAAAAGAAACTGGCACAACAATAG
CTCGCAGAGCAGGGGTTCAAAACGAAAACTAAAGGATGATAATGCCGGAGGA**

>SpKNOX1|translation
IGSLDNISRVSNPNNILLNLTEDKFNDALLSNGIPSNLPQTIDNLSHDYKFHELNNFSSIQHFC
SMNEHTAQDSRLEFSCPTSLMNDELQNVERALSCRNDSTCSSSLQNLETGYLPKLRTGTY
AAQEETTLTAFENGLNNEPNGLVYNFDYGANQNIYQGSAEVENTEASHLANAYPTDFHVFQ
NVNQTSRSGDGNNGFERPEPLAAQKVDEDEKINAIKAEVVSHPLFNQLLEAHVACLKVATP
LDQIPLLDLQLEKKNLVIEKYEKFLKDPKYGNFKNKIEETTELNFFMTHFVCLLQSFKSQLEQ
HVLIQAREAVMSCWELEQSLISLTGMEPRPGTGLTMSDDDDDDLDDEDTIDYDKHASETLT
DGNQDPYGPLTPSENERSIMERMRTELKKQLSDQGYRTKIADVREEIMRKRRAGKLPSDTT
QILKSWWNAHSKWPYPTEDEKTTLVRETGLELKQINNWFINQRKRNWHNNSSQSRGSKRK
LKDDNAGG

>SpKNOX2|cDNA
**TTTTCTTCAAAGCCTAAGCATAATTCATTAAATTCGTTAAGATTAATTGCGCCCTTTCA
ATCAAGACAGGACATGGATGAAGTCATTCAGCTTGACTGCTTAGAGAGAACAAATAA
TCTTAACGAATTTAATGAATTATGCTTAGGCTTTGAAGAAAACTATTCTTTTGCTTCAC
CATTTGATTGTAATTTTTTTGAAAATCCATTTTCTCTTTCTACCGATCTAAGTGGAAGTC
CTATGGCCGATGATCTTCTGCTATTAGATTACAACTTTTTTCAATCATCTGAGATTCAA
GGAATAAACATGGAAGAAACTTATGAACAATTTTCTTTGATGGAAGAGGATCAAAATA
ACGCAAACCAAAATAATAATCCAAAATTTTGTGGGTATAAAGAGCAAATTATTTCACA
CCCATTGTTTCCTGACCTCGTTTCAAAATTCATCGAATTACGCAAAGTAGGGGCACCT
GAAGAAAAAATGAAAGAACTAGAAAATGAACGTGAAAGTCTTTTAAATTTAATGAATG
GGAAAATGATGATGAATGATCATGATGAAAATTTGGATTCTTTTATGAATGGTTTTTG**

GTTCAATTGTCTGAATCGAAAGTTGAAATTCAAAAAATCTTTGACGAGGCTACTAAATT
TTGCTCAGATTTTGAGAAGAATATTTGCCAAAATTTGATGTCAAATAAAGAAAAAGAA
AATGAAGAAGAGGATTTTCAAGACGTAGATCCTACCGCTGAAGACAGGGAATTGAAG
GAGCAGCTGAAAAGAAAGTATAGAGCTTATTTGACGGGTTTAAAACAAGAATTCTTAA
AGAAGAGAAAGAAGGGGAAGCTTCCAAAAGAAGCCACCATGATTTTGCATAACTGGT
GGTTTTCTCACTTAAATTGGCCTTATCCGTCGGACGCGGAGAAAACACAGCTAGCGG
AAATGACCCGTCTTGACCCGAAGCAAATTAATAACTGGTTTATAAATCAACGGAAGCG
ACATTGGAACAACGCACCTAACCCGAACCAACTTACGTTA

>SpKNOX2|translation
FSSKPKHNSLNSLRLIAPFQSRQDMDEVIQLDCLERTNNLNEFNELCLGFEENYSFASPFDC
NFFENPFSLSTDLSGSPMADDLLLLDYNFFQSSEIQGINMEETYEQFSLMEEDQNNANQNN
NPKFCGYKEQIISHPLFPDLVSKFIELRKVGAPEEKMKELENERESLLNLMNGKMMMNDHD
ENLDSFMNGFLVQLSESKVEIQKIFDEATKFCSDFEKNICQNLMSNKEKENEEEDFQDVDPT
AEDRELKEQLKRKYRAYLTGLKQEFLKKRKKGKLPKEATMILHNWWFSHLNWPYPSDAEK
TQLAEMTRLDPKQINNWFINQRKRHWNNAPNPNQLTL

A. agrestis KNOX

>AagKNOX1|cDNA
TCGAGGGCAGTCCTCTGGCAGACCGTGCTGCTGAGAGCAACAGGAGCCTGCTTCAGG
CTGCTTTCCAGCCCGACCCTGCTCAA**ATGATCAAGCGTCCAACCTGGCTCGCCAATGC**
CGTTCTGCGACTGCAGGCACAGCAAGCTTCAGCAGCAGGGAATGGGACAGGAGAAT
CAAATATGGGCTCAAATGGAAGCCAGGAAAATGGAAGCTACGGGGGGCACTGGCTT
TCGATGCCTGTTGCACACTACGAGAATGGGAGCAACGGCAGTGCATCGCCTCCTCAG
GGGGGAGATCTGGTGCAGCAGGGGTTCAAGAACAACTCTATGTACAATGGATCAAGC
TTGAGGACTCAGTATGACAGTGCTGAGAATGGTTATAGGCAAGGACACGGAGGAGAC
GACAATGGTGATGGAGACAACAGCAGTGAGCCCAACCACGACACAGAGGACCAGGA
GAACTGGGTGTTGGCAAAGAGAAAAGCGGATATTGTAGCGCATCCTCTGTTTCCCGA
GCTTCTGTCTGCTCATGTCGCTTGCTTGCGCATCGCGACACCTGTGGACCAGCTGGGA
ACCATCGATGCACAGCTGTCGCAGTCACACCTTGTAATCAGCAAGTACCAGGCATTG
AGCCTGGAATCACGACCAGAAGACAATCCAGACAAGGAGGAGCTGGAGCAATATAT
GGCAGACAACGTCATGCTGCTCCGTTCTTTCAAGGACCAGCTGCAGCAACACGTTCG
AGTACATGCCATGGAGGCTGTAATGTCTTGCTACGACCTGGAGAAGCAACTTGAAGA
GCTGACTGGCGTTCGGGCAGGTGAATCTGGACAAACCTCTGGTTACCTGATGTCAGA
TGACGAGGATGACGCACATGGAGATAGTGATTTATCTGCTTATGATCCCAGCCTGGAT
GGGGCCGAGAATGGGGGCTTCGGGCCATTGATACCTACTGAAACAGAGCGCACTCT
CATGGAACGCGTGCGGCAGGAGCTTAAGGCAGAGCTCAAACAGGGATACAAAGCCA
AGATCATAGATGTGAGGGAGGAGATACTGCGAAAGCGTCGAGCCGGCAAGCTGCCA
GGTGATACAACTACGGTGCTGAAAACATGGTGGCATGCGCATGCTAAGTGGCCCTAC
CCTACGGAAGACGAGAAGGCACGCCTGGTGCAGGAGACTGGTCTGGAGCTGAAGCA
AATCAACAACTGGTTCATCAACCAGCGCAAACGCAACTGGCATGCTAATCCTTCTGCT
GCAGCCGCAGCCAAGACTAAACGCAAGAGCTAGGTTTGTGATCTTGCTGAAAAGGAT
GCTAGTACTTGTGTTGCAGGTGACAACCTCCTGTACTTCTAGCAACTCATCCAACGACG
GAGCTTGCAGCACTTGAATG

S. kraussiana BELL

>SkBEL1.genomicDNA
TAATAGAATTTGGAAATTACTTACCGGCAAGAATCTTATAGAAAGTATTTACTATAATAAC
TCTTATCATGCTCCAGTATGGATCTACCCCAGGAAGGTAGAACCTGGACGTTAAATTTA

CATCAATCCATCCATCTAATAAAATATTAAAAATATGTCTAGATTGGGGGGAGAAAGAAC
ATGTTCAATGTAATGACAAATGGGAAATGTGGAAGCTGAAAGATATGTTCCAACGAGAG
GAGCAGACAGAAGCAGACATGGATAATGTGAAGCTCATCGAACCCGTAAGAGAGAGAG
AGAGATTTCTTTTATCGTCCACGGGAACCCAACAGGGTTACGGACACTCATATAATAC
AAAGTATGGGATATGGACATTGGCTTTTGAAATCTGTACTGGCGGTTCACAGCTGGATT
AAAAAACCCCCACCCCCCGTAGAGTTAATAGTGAGGGAAAAACCGTGCTTGAAACCTTG
TTGAACCAGACAGGATATAAGTGCTTGTTGTGTACCTTGATTGCCATCATGATGTAAGC
CTAGTACCATATCCCTGTCAGTCAACATTCAACCTCTTCTAGAAGACTGAAGTTCTTGCA
TCTCTCAGATATCGGACTCCGGCTGCTGGAGCTCAAGGTTACGGAAGCTGGAGAAACG
GGGACGAGCTGATGTTNANGNGGGGAAGCGAGCGGGCCAAGAGCTTCGGCAACAATC
AGGAGAGCAGCCcctgagtcgtATAAATGAGNTTGGGNATACGGTATACGCGAAACACCGT
CTTTGGGAGGACAGCAACACCAGCTTCTGCCGGTGGAAAACTCGCAGGCGGGTGGAA
CTCCGGGGTACCAATCTCTCTCGCTTACGCTTTCTTCACAGCAGCCTCAAGCCCTACCG
GGTTACGACGTTTTGAGCCAGACGGCCGGGCTCGAAAGTCCGGCG**ATGTCTCAGGAT**
GGAAAGAGGCAGATTAGTGGGAAGCTTAGTGGTGATTTAAGCCACTTTCGACGCGAT
TTTGGGTCCAGCAAGTCGGTTCAAATCGACTCCGGAACGTCGGCTATGTCGGCGAGC
TTCAGCATCATCTCCAGCAGTCGGTATCTCAAGGCATGCCAGCAGCTCCTTGAGGAA
GTCTGTAGTGTTGGACGAGGGACGAAGAATGGGAAGCCGAGGACGGCTGCTGGGCC
TAGTCCGTCCTTGACGAGTCCGACTTCGGGTGGCCGAGATGAGAACTCGTCCGGAGC
CAAAGACAATAGTACAGGCGGTGCTACTACCGCGACCAATGCCGCGACTGCAGCTG
CTACAACCACGAGCGCAGGCCCCGCAGTTTCTTCTCCGAAAACCAAAGGTCCGACTG
TTACTGCACCGGCGACGTCGACCTCGGAGCCGGGGGAGTCCGCGAGTACGCTCACC
ACCGAGGAGAGGCAGGAGTTCGAGCTCAAGAAGTCGAAACTCATTGCGATGCTTCAA
GAGGTACGTTTACCATTGCAANGTACATATACGTAGTATACTTAACCTTTTGAATACGT
ATTTTCAGGTTGATAGACGATACAGACAATACAACGATCAGATGCAAGTTGTGATCAG
CTCGTTCGACGCCGTAGCCGGCACGGGCTCAGCAACTCCGTATACGGCACTGGCTCT
ACAAGCCATGTCTCGCTACTTCAGGTGCCTCAAGGATGCGATCACAGGGCAGATCCA
ACTCGTTTGCAAAGCTCTCGGCGAGGAGGACATCTCGAAGCAGCTCACCACCAAAAG
TCCGACTTCGAGACTGCGGTTCATCGATCAGCAGATTCGACAGCAGCGAGCTTTCCA
GCAGCTGGGGATGCTCAACCAGCACGCTTGGAGGCCCCAACGAGGACTTCCCGAAC
GCTCCGTCTCCATCTTGCGAGCTTGGCTCTTCGAACATTTTCTTCACCCGTGAGTATNT
ATTTATCTTTTAAGTAGTATTTAGAGTAGTATTTATTTTTATAGTGGTTTTTTCGCAGGC
GTTACGTTTCCCTTAGATTTTGCAAGCCTGTCATTCTTGCATAATTTGTCTTTACATTGT
TGGCTGCCTAGATTTCCTCGCATGATTTTTCGAGCTCAGTTTTCATTGATTTTTCAGGT
ATCCAAAAGACGCAGACAAGATGATGCTTGCCAGGCAAACTGGTCTCACGAGAAGCC
AGGTAATGATACGTAACGGCTGTATTTTATACGTATTTCGTATCTGACGGTGTATTATT
GTACCCCAGGTTTCTAACTGGTTCATCAACGCGAGGGTGAGGCTGTGGAAGCCAATG
GTGGAGGAAATGTACCAGGAGGAGACGAGAGATCACGAACTCGATGCAGCGGTTTC
GAGGATGCATGCCGAGAACGAAGAAGCGGCGGCCGCAGCTGCAGCCGGTTACCATC
ATCGTGTCGGAGATGCAGCAACCGGTGGTGACAAGTTGGATCAGCAGCAGCAATCC
GGTCTCGGAGACAAGTCCAAAGATCTCGACTACGTCGAGAACTCGGACTATCTGAGG
TCCAAGCAGAGCTTGAACTCCATGCATCAGCATCCGGGGGACTCTCAAATGTACGAG
ACGCAGCAGCAGCAGATCAAGAGGTCGAGGTTTGGAGCTCACGAGAATAATGAAAT
GTTGCAGCTTCCATCTTCGAACTCGGCGAGCATGGACGTTGAGATGAAGCCGGACGT
GCTACCGGAAGCTCACCACCATTTGCACCACCACCACTTGCTGCCTCAGGCCCAACA
GCAGCAACAGCACGGGTTTGGGTACGACGGTCTTCGTTTCAACGCCGGTGCCGGATT
CGGCTACAGCCATCCGGCAGCAACAAGCGGGGGTGTTTCGCTCACTCTTGGTTTGCA
GCACAGTGAGAACAACAGTGGATTATCTACCGGAGCTTATAGACAGGCTTTTCTCCAA
CACCAACAGCAGCAGCAGCAGCAGCACGACTACGCTAGCTTTGAGAATGCAAGCCT
GATTTCATGAATCACTTACACATTTAATAGAAAACCTCCTTGTAGTCGTCTATTGATTGAT
TTGTAGATCTGTGGAAGATCGACAAAATGGGATTTCAAGGTCCTATATAGGTGGATTCC
GAGAGGTTATGCCAATACGAGGGCGGTTTCATAATAGGCTTTCATAATTGGAACATGGT

GATGGAGGTACACAAAGAATGACAGGCTTGCAAATCACAATGTAGTAGTTTCCATTAGA
GTAACACATTGTACACATGGTAAAAAAAAAAAAAAAACACATTGTACACATGGNGGTTAT
GTTTTAATTAATATACATTTTCTAATAGAATTTGGAAATCACTTACATTAAGTATTTACTAT
AATAACTCTGGTATTATCATAATGCTCATTATGATAATACCATTACCCTGTTGAAGGTATG
ACCTCGACATAAAAATAAACAAATCTATCTATTAGATTAGATATTTTTAATAAAATATTTAA
AATACTGCATTATATGTCTAAATTGAAGAGTCTAATAAAACACTGACATTTTTTTCTTGAC
CCAACTAAAACCTTCCTTTTTCACTTATATAATTTAATTTATATAATTATATAATAAATTGA
ATTATCTTTTAAACTTATACAGGTTATATTTAGAGCCCGNTNCTCAAGCATACGCAAAGA
ATGAAATCACAAAGTACACATAGTGGTTATGTATTAATTAATATACACACTTACTAATAGA
ATTTGGAAATTACTTACCGGCAAGAATCTTATAGAAAGTATTTACTATAATAACTCTTATC
ATGCTCCAGTATGGATCTACCCCAGGAAGGTAGAACCTGGACGTTAAATTTACATCAAT
CCATCCATCTAATAAAATATTAAAAATATGTCTAGATTGGGGGGAGAAAGAACATGTTCA
ATGTAATGACAAATGGGAAATGTGGAAGCTGAAAGATATGTTCCAACGAGAGGAGCAGA
CAGAAGCAGACATGGATAATGTGAAGCTCATCGAACCCGTAAGAGAGAGAGAGAGATT
TCTTTTATCGTCCACGGGAACCCAACAGGGTTACGGGACACTCATATAATACAAAGTAT
GGGATATGGACATTGGCTTTTGAAATCTGTACTGGCGGTTCACAGCTGGATTAAAAAAC
CCCCACCCCCCGNAGAGTTAATAGNGAGGGAAAAACCGNGCTTGAAACCTTGTTGAA

>SkBEL1.cDNA
ATGTCTCAGGATGGAAAGAGGCAGATTAGTGGGAAGCTTAGTGGTGATTTAAGCCAC
TTTCGACGCGATTTTGGGTCCAGCAAGTCGGTTCAAATCGACTCCGGAACGTCGGCT
ATGTCGGCGAGCTTCAGCATCATCTCCAGCAGTCGGTATCTCAAGGCATGCCAGCAG
CTCCTTGAGGAAGTCTGTAGTGTTGGACGAGGGACGAAGAATGGGAAGCCGAGGAC
GGCTGCTGGGCCTAGTCCGTCCTTGACGAGTCCGACTTCGGGTGGCCGAGATGAGAA
CTCGTCCGGAGCCAAAGACAATAGTACAGGCGGTGCTACTACCGCGACCAATGCCG
CGACTGCAGCTGCTACAACCACGAGCGCAGGCCCCGCAGTTTCTTCTCCGAAAACCA
AAGGTCCGACTGTTACTGCACCGGCGACGTCGACCTCGGAGCCGGGGGAGTCCGCG
AGTACGCTCACCACCGAGGAGAGGCAGGAGTTCGAGCTCAAGAAGTCGAAACTCATT
GCGATGCTTCAAGAGGTACGTTTACCATTGCAAGTACATATACTTAGTATACTTAAGC
TTTTGAATACGTATTTTCAGGTTGATAGACGATACAGACAATACAACGATCAGATGCA
AGTTGTGATCAGCTCGTTCGACGCCGTAGCCGGCACGGGCTCAGCAACTCCGTATAC
GGCACTGGCTCTACAAGCCATGTCTCGCTACTTCAGGTGCCTCAAGGATGCGATCAC
AGGGCAGATCCAACTCGTTTGCAAAGCTCTCGGCGAGGAGGACATCTCGAAGCAGCT
CACCACCAAAAGTCCGACTTCGAGACTGCGGTTCATCGATCAGCAGATTCGACAGCA
GCGAGCTTTCCAGCAGCTGGGGATGCTCAACCAGCACGCTTGGAGGCCCCAACGAG
GACTTCCCGAACGCTCCGTCTCCATCTTGCGAGCTTGGCTCTTCGAACATTTTCTTCAC
CCGTATCCAAAAGACGCAGACAAGATGATGCTTGCCAGGCAAACTGGTCTCACGAGA
AGCCAGGTTTCTAACTGGTTCATCAACGCGAGGGTGAGGCTGTGGAAGCCAATGGTG
GAGGAAATGTACCAGGAGGAGACGAGAGATCACGAACTCGATGCAGCGGTTTCGAG
GATGCATGCCGAGAACGAAGAAGCGGCGGCCGCAGCTGCAGCCGGTTACCATCATC
GTGTCGGAGATGCAGCAACCGGTGGTGACAAGTTGGATCAGCAGCAGCAATCCGGT
CTCGGAGACAAGTCCAAAGATCTCGACTACGTCGAGAACTCGGACTATCTGAGGTCC
AAGCAGAGCTTGAACTCCATGCATCAGCATCCGGGGGACTCTCAAATGTACGAGACG
CAGCAGCAGCAGATCAAGAGGTCGAGGTTTGGAGCTCACGAGAATAATGAAATGTTG
CAGCTTCCATCTTCGAACTCGGCGAGCATGGACGTTGAGATGAAGCCGGACGTGCTA
CCGGAAGCTCACCACCATTTGCACCACCACCACTTGCTGCCTCAGGCCCAACAGCAG
CAACAGCACGGGTTTGGGTACGACGGTCTTCGTTTCAACGCCGGTGCCGGATTCGGC
TACAGCCATCCGGCAGCAACAAGCGGGGGTGTTTCGCTCACTCTTGGTTTGCAGCAC
AGTGAGAACAACAGTGGATTATCTACCGGAGCTTATAGACAGGCTTTTCTCCAACACC
AACAGCAGCAGCAGCAGCAGCACGACTACGCTAGCTTTGAGAATGCAAGCCTGATTT
CATGAATCACTTACACATTTAATAGAAAAAAAAAAAAAAAAAGTCGACATCGATACGCGT
GGTCATCTGC

>SkBEL1.protein
MSQDGKRQISGKLSGDLSHFRRDFGSSKSVQIDSGTSAMSASFSIISSSRYLKACQQLLEEV
CSVGRGTKNGKPRTAAGPSPSLTSPTSGGRDENSSGAKDNSTGGATTATNAATAAATTTS
AGPAVSSPKTKGPTVTAPATSTSEPGESASTLTTEERQEFELKKSKLIAMLQEVRLPLQVHIL
SILKLLNTYFQVDRRYRQYNDQMQVVISSFDAVAGTGSATPYTALALQAMSRYFRCLKDAIT
GQIQLVCKALGEEDISKQLTTKSPTSRLRFIDQQIRQQRAFQQLGMLNQHAWRPQRGLPER
SVSILRAWLFEHFLHPYPKDADKMMLARQTGLTRSQVSNWFINARVRLWKPMVEEMYQEE
TRDHELDAAVSRMHAENEEAAAAAAAGYHHRVGDAATGGDKLDQQQQSGLGDKSKDLDY
VENSDYLRSKQSLNSMHQHPGDSQMYETQQQQIKRSRFGAHENNEMLQLPSSNSASMDV
EMKPDVLPEAHHHLHHHHLLPQAQQQQQHGFGYDGLRFNAGAGFGYSHPAATSGGVSLT
LGLQHSENNSGLSTGAYRQAFLQHQQQQQQQHDYASFENASLIS*

>SkBEL2.genomicDNA
TNNCAANCNAATGTCACNTCAATANAGTCTCNCTCCGNTCNGATCNGTTCGTTATGTNA
NCCNTCGNTGACCAGTGGNANTCGAGGCTAGACCGACCGTCTTTTGGAGTANGTGCCC
GAGACAGCCCCCAGACACTGACTGCCGAAAGNNTCAAGCNCNTCATGCCACCCCAGAC
ATGNCNGTGTGTCGAGCCCACCACCACCACCAGGTCACCACCACCATCACCAGATCAC
CAGAGNTACNGTGACTACNGTAACATGATGAAGAAAACCCGGTTGGGGGGGATCCACG
ACTCTCTGGGAATGCTGGACGCTGGTGGTAGTACCATGGATGTCATGGAAGTCAAGGA
TGGTGATGGTAGTATGGATGGCAGTAAGTATGGGGCGTATATACAACATGATCAGCAG
GCTCAGAACTACGGAGTTCATCAGACTACGGTGGATAGCTACGGTAGATACGGCGGTG
CACCGTCGGGTGGTGGAGGGTACGCTACGCAATACCCCGGGGGTAGTGGGGTGTCCC
TCACACTCGGACTACGGCATTGCGATAATCTTCATCTCGCAGCTGCTGCTGGGCCTCAT
CAGTTCTTGCAGCAAGGAGGACAGAACTTTCTGGCCGCTGCCGGACGTGGTGCGATCT
CGGCGTCGGATGGAAGTGAGCTTTGTAACATCTACGGAGCGGCGGCGGCTGCTGCTG
GTGGCGGTGCTCACATTCACATCGGAGAGCACGATGGTATGGACTACCAAGGCAGGAA
GCAGCAGCATATGGAAGGCCACCTCTTGCATGACTTTGTGGCATGAGAAAAACATGCAT
TTTTCTGCAGCTAGCTTCGTCCATTTTCAGGAGGATGTCACAGCAGAGCTGCAAAACAT
TGGTGGTGGCCTTGATTGAGCTCGCGAAAATTACTTGTATTACTTCAGCAGCAACATTG
GCTGTAAAATCCATACGACTATACCACGACGGCGTAGTATGCTTGTATGTACACACACC
ACATTGAGAAATGTCGGAAGTTGTTCATCTGCAAGATGGGAGGAATGCGCCACCGCAG
AAGAACGCTGTTGCAATGTAGCAGATGATGATGACTGTTATGATTAGAAGTATCCTGGA
AATTGTTGCCGTATGTGAGGGGATATATACGATGGTTTTTCTACGATTTACTTACACTAG
CTTGCACGTATTCATGCCTTGTGTCCGTCGAATCTTCCGAGCTTGTTTGCGGAATCTTAA
GGAATTTTCTTGTATGTGTG<u>**ATG**</u>**TCTTGTCCACCAGGAGCTCTAGCCTATCACCACGTT**
CCAAGATCTTGTCGATGTTTTGAACCATCACCGCTCGAACCTGACATGTGTAAAAGCT
ATAGTAAAACCATTGAAAAAACTGGAGCCTTTCTCAACTAAACAATATTGAAAACATG
TAAAATTATGATCCAGGTATGAACTAAANCCATTGAAAGAATGGGAGCCTTTTAACCA
TTCACTATTGAAAACACATAAAATTCNTCTTGAAAATATGGTAGCTCTGAAAACTATGA
CATTGTCAAGTTCTCTTGCATTCTCAAGTGCTCGAATAAAACTTTTACTCTTGAAAACA
TGAGCTATGGAAAAAACACCTAAAAACGAGAAGCACTCTTGAAGTGCTCTAAAAACT
TAGCTTTTGGAAAGCTCAACTGTTCGAAAACTAGAGACAATCATGAAAACATGAGTTT
TTTGGAAGCCTCAAGTGCTTGAAAACCACCTAAAATCTAACAACACTTACATCCGTGA
TTTCTCCTTTGACACGATTAATGATGTCTGCCTGAGGATCGTTCGAGAAGTGATCCAT
CTTCTGGTGCAAAACTCTCGAGTACCTTCAGCTTTGTGTCCGGCAGCCGATACCTTAG
GGCGGCGCAGCAGCTCCTCGACGAGGTCTGCAGTGTCGGACGAGGCCTGAAGGGGT
CCAAGGCTGGTAGGGGCTCGCAAGGTACCGGTGGTAGCCCAACTGCCATTACCAGC
GGTTCTGCACGAAAGGAGGTGACTACTGGTGTAACCACCGAGAACAACTCGGTTAAA
AGCGAAGTTACCATGGGATCATCGGTGGTGCCTAGTCCCTCCGGACCGTCAGCTGCA
GCAGCTACGTCGAGCGTGGTTGCACCGTCGAATACCACCGCCGAGAGCAGGGACAA
CGTCGCGGTATTAACTCCCGAGGAGAAGCAAGACCACGAAGGGAAGAAAACCAAGC

TTTTGGCTATGCTTCAAGAGGTATGGATTCGTAGTTGAGCGCTGATGACGACGGGCCA
TTGAGTTTTGTAGTATTTCTCAGGTCGATCGACGGTATAGGCAATACTACGATCAAAT
GCAGGTGGTCATAACTTCGTTCGATACGGTAGCTGGTGTTGGAGCTGCTACACCGTAT
ACCGCACTTGCTCTACAAGCAATGTCCCGGTATTTCAGGTGAGCTTTCATCGTACAAA
TACATGCGAATTTCGTAGTTTCATACTCTCGGTATATTGATTTTTCTACGGTTTTTTCAG
GTGTTTGCGAGATGCCATTACCGGGCAAATACAAATAACTTGCAAAGCTCTCGGTGA
GGAGGACGTAACAAAGAGCATCACCAGCCGGCCGCTAACTTCGAGACTACGGTTCAT
CGATCAGCAAATACGGCAGCAACGAGCATACCAGCAATACGGGATGCTTCAACAACA
TGCCTGGCGGCCGCAACGAGGATTACCCGAGCGTTCCGTCTCTATACTGCGAGCCTG
GCTTTTCGAGCACTTCCTTCATCCGTAAGCATATTCGTATATATTCGTATGTATTCATGT
AATATACGGTTGGTTGGTGTTGGTCAGGTACCCGAAAGATGCCGACAAAATGATGCT
GGCTCGGCAAACCGGTTTGACACGGAAGTCAAGTAAGTGATTCCATTCCAGCACGTT
ACCACGTTGCTTACCATGGTAACTACCACGGCTCTGCAGGTTTCTAATTGGTTTATAA
ATGCCAGAGTCCGGCTTTGGAAGCCAATGGTAGAGGAGATGTATCAGGAAGAGACTA
AAGAACACGAACTCGGTACTTCGTCTGTGAGGCTGAGCGTTGACACCGAGGACAAGC
ACCACCAGGACAACATGGACTTGCACGAAAAGCTATCAAAGCACCATCATGACCACC
GCCAGGACATGGCCGGTGTGGTCGAGCACCACCACCACCAGGGTCACCACCAC
CATCACCAAGATCACCAGAGTTACGGTGACTACGGTAACATGATGAAGAAAACCCGG
TTGGGGGGGATCCGAAGAGGTCTGTGCAAAAGCTTTCCAGCGATCTCAGTCACTTTC
GGACCTCGCCCAGGGACACCCTCGCAGGTGAGGGTTTGATGGTGTTTTTAAGTGGTTA
CGTTGGCTCCCAGTGTGGTTTTTACTTGTTTCTGTTAGTTTTTGTGTTGTGTTATTGATTT
TTACC

>SkBEL2.cDNA(predicted)
ATGTCTTGTCCACCAGGAGCTCTAGCCTATCACCACGTTCCAAGATCTTGTCGATGTT
TTGAACCATCACCGCTCGAACCTGACATTGATCCATCTTCTGGTGCAAAACTCTCGAG
TACCTTCAGCTTTGTGTCCGGCAGCCGATACCTTAGGGCGGCGCAGCAGCTCCTCGA
CGAGGTCTGCAGTGTCGGACGAGGCCTGAAGGGGTCCAAGGCTGGTAGGGGCTCGC
AAGCAGCTACGTCGAGCGTGGTTGCACCGTCGAATACCACCGCCGAGAGCAGGGAC
AACGTCGCGGTATTAACTCCCGAGGAGAAGCAAGACCACGAAGGGAAGAAAACCAA
GCTTTTGGCTATGCTTCAAGAGGTCGATCGACGGTATAGGCAATACTACGATCAAATG
CAGGTGGTCATAACTTCGTTCGATACGGTAGCTGGTGTTGGAGCTGCTACACCGTATA
CCGCACTTGCTCTACAAGCAATGTCCCGGTATTTCAGGTGTTTGCGAGATGCCATTAC
CGGGCAAATACAAATAACTTGCAAAGCTCTCGGTGAGGAGGACGTAACAAAGAGCAT
CACCAGCCGGCCGCTAACTTCGAGACTACGGTTCATCGATCAGCAAATACGGCAGCA
ACGAGCATACCAGCAATACGGGATGCTTCAACAACATGCCTGGCGGCCGCAACGAG
GATTACCCGAGCGTTCCGTCTCTATACTGCGAGCCTGGCTTTTCGAGCACTTCCTTCA
TCCGTACCCGAAAGATGCCGACAAAATGATGCTGGCTCGGCAAACCGGTTTGACACG
GAAGCAGGTTTCTAATTGGTTTATAAATGCCAGAGTCCGGCTTTGGAAGCCAATGGTA
GAGGAGATGTATCAGGAAGAGACTAAAGAACACGAACTCGGTACTTCGTCTGTGAGG
CTGAGCGTTGACACCGAGGACAAGCACCACCAGGACAACATGGACTTGCACGAAAA
GCTATCAAAGCACCATCATGACCACCGCCAGGACATGGCCGGTGTGGTCGAGCACC
ACCACCACCACCAGGGTCACCACCACCATCACCAAGATCACCAGAGTTACGGTGACT
ACGGTAACATGATGAAGAAAACCCGGTTGGGGGGGATCCGAAGAGGTCTGTGCAAA
AGCTTTCCAGCGATCTCAGTCACTTTCGGACCTCGCCCAGGGACACCCTCGCAGGTG
AGGGTTTGATGGTGTTTTTAAGTGGTTACGTTGGCTCCCAGTGTGGTTTTTACTTGTTTC
TGTTAGTTTTTGTGTTGTGTTATTGATTTTTACCAAAAAAAAAAAAAAAAAA

>SkBEL2.protein(predicted from cDNA)
MSCPPGALAYHHVPRSCRCFEPSPLEPDIDPSSGAKLSSTFSFVSGSRYLRAAQQLLDEVC
SVGRGLKGSKAGRGSQAATSSVVAPSNTTAESRDNVAVLTPEEKQDHEGKKTKLLAMLQE
VDRRYRQYYDQMQVVITSFDTVAGVGAATPYTALALQAMSRYFRCLRDAITGQIQITCKALG

EEDVTKSITSRPLTSRLRFIDQQIRQQRAYQQYGMLQQHAWRPQRGLPERSVSILRAWLFE
HFLHPYPKDADKMMLARQTGLTRKQVSNWFINARVRLWKPMVEEMYQEETKEHELGTSS
VRLSVDTEDKHHQDNMDLHEKLSKHHHDHRQDMAGVVEHHHHHQGHHHHHQDHQSYG
DYGNMMKKTRLGGIRRGLCKSFPAISVTFGPRPGTPSQVRV
