# Supplementary material for: Nonreciprocal complementation of KNOX gene function in land plants
Source: New Phytol. 2016 Nov 25;216(2):591–604. doi: 10.1111/nph.14318 (PMC5637896; doi:10.1111/nph.14318)
Supplement: Supplementary file 7 — Notes S5 Edited KNOX alignment. [file NPH-216-591-s007.pdf]

New Phytologist

Supporting Information Notes S5 Edited KNOX alignment.

>kfl00118_0070
--------------------------------TCGTACCCAAGCTTATCGCAGCTCATGTAGCCTGCGTGCAGATGGACA
CCTCCTCGCCGCGGCTGTATCAGCTGGAGCTGGACCTGGCAGCCGCCCATGGAGCATCgGAGC
AGCCGCGCCGGAGGTGGATGCGTTCATGGCAGAGTGCTTGGCTCAACTGGGGCGGCTCAAGGC
GGCTCTCGAGAGCCACATCTCC------------GCAGCTCTGCGGTCCTGTGAGGTGTTGGAGCAAAGAC
TAGTGGACGCTTTGGGCCACCGTGCACCCGAACCCGCGGTGGCGGCCAGCATGGCCAAGGAGC
TGCGGCACAAGCTGAAGAAACAGTACAGCGAGACCCTGGCGGAGGTGCACAAGGAGATCTTGCG
CAAGCGGCAGGCGGGGCGGCTGCCCGGCGACCAGGGGCACCTGCTCAAGGACTGGTGGAGGC
GCCACGAGAGCTGGCCCTACCCCACGGATGACGAGAAGCAGGCCCTCGCAAGGCAGACGGGCC
TGTCAATGAAGCAAGTCAGCAACTGGTTCATCAATCAGCGaaAGCGGAACTGGAAACAC------
>kfl00113_0180
TACCCCGAGGCAGACGCGATTCGACGCCACGATAGGTATCCGGAGCTGGTCGGCCGGTTGATGG
ACTGTTTGAAAGTCGGCGCGTCGGAAAACGAGATCCGACGGCTAGACGCGCATCGGCGGTGCCT
GGTGGAGGAGTCCCGGGCGCGCGAGCCCGGGTTAGATAAGGTTATGGAGGTCACGTGCCAGCA
GCTCGTGGCGCTCAAGCAGCAGCTGCAGCAGCCGTTCGAC------------GACGCGAACGCGTTCTGC
AGTGACGTCGAGCGGCGCCTCGCGGAGATCGGGCTCTCCCGGGCGCTGGTGGGCGCGGCCGG
CCCGGCCGACATGGGCGCGCTCAAAGCGCGCATGATCGCGCTGCACGGGCCCGCGATCGCGCG
CATGGACATCGACACGTGGAAGAAGGAGCCGAAGGCGCGCCTGCCGGCCGCCGCGATCAAGGT
CATGCGCGGCTGGTGGGCCAAGCACCAGGACTCGCCGTATCCTGCGGAGAACGAGAAGCAGCG
GCTGGCGAAGCGCGCGGGCCTCGAGCTGCAGCAGGTCAACAACTGGTTCATCAACGAGCGGAAG
CGCCACTGGACCAAGGCCGAG
>AaKNOX1
agcgatatgggagagcaggtgataatgcatcctttgtacccggacctggtaaaggctataatggactgtcgaaaagttggtggaatggacgagt
ctagacaccacatccaaatccgcaccgaataagtgctggaagatttacacgcgctagaccccgaactcgaccaatttctacgacaatacatcca
agtactcgacgaactgcacgcagagctcctaaacattaaccga------------gaagcggacaacattttacacatgttcacgacctaaatagcg
gaggtcatcaacatgcccatggacatgacctggttcgaaattcgcaacgaacaagaataacgcgtgctcctcaaacaaaaatatcggtaggaa
ctcctagctttaaaagaagagttttcgaaacgtaaaaaacgtggcaaattgccgacgcattcgatcgaggttttaaaaagttggtggaaggaaca
catcgcgtggccatatcctaccgatagcgcaaaacgttcattggcctcttaaaccaatttaacgtctattcaaattaataattggttcattaatcaacg
caagcggcattggcacaaactttt
>AagrestisKNOX
GCAAAGAGAAAAGCGGATATTGTAGCGCATCCTCTGTTTCCCGAGCTTCTGTCTGCTCATGTCGCT
TGCTTGCGCATCGCGACACCTGTGGACCAGCTGGGAACCATCGATGCACAGCTGTCGCAGTCAC
ACCTTGTAATCAGCCCAGACAAGGAGGAGCTGGAGCAATATATGGCAGACAACGTCATGCTGCTC
CGTTCTTTCAAGGACCAGCTGCAGCAACACGTTCGAGTACATGCCATGGAGGCTGTAATGTCTTG
CTACGACCTGGAGAAGCAACTTGAAGAGCTGACTGGCGTTCGGGCAACAGAGCGCACTCTCATG
GAACGCGTGCGGCAGGAGCTTAAGGCAGAGCTCAAACAGGGATACAAAGCCAAGATCATAGATGT
GAGGGAGGAGATACTGCGAAAGCGTCGAGCCGGCAAGCTGCCAGGTGATACAACTACGGTGCTG
AAAACATGGTGGCATGCGCATGCTAAGTGGCCCTACCCTACGGAAGACGAGAAGGCACGCCTGG
TGCAGGAGACTGGTCTGGAGCTGAAGCAAATCAACAACTGGTTCATCAACCAGCGCAAACGCAAC
TGGCATGCTAATCCT
>AtKNAT1
GAAGCCATGAAGGCTAAAATCATTGCTCATCCTCACTACTCTACCCTCCTACAAGCTTACTTGGAC
TGCCAAAAGATTGGAGCTCCACCTGATGTGGTTGATAGAATTACGGCGGCACGGCAAGACTTTGA
GGCTCGACAACAGTCTAGAGACCCGGAGTTAGATCAATTCATGGAAGCATACTGTGACATGTTGG
TTAAATATCGTGAGGAGCTAACAAGGCCCATTCAG------------GAAGCAATGGAGTTTATACGTCGTA
TTGAATCTCAGCTTAGCATGTTGTGTCAGAGTCCCATTTTACCGGAAATAGACCCGAGGGCCGAAG
ATCGGGAACTCAAGAACCATTTGCTGAAGAAGTATAGTGGATACTTAAGCAGTTTGAAGCAAGAAC
TATCCAAGAAGAAAAAGAAAGGTAAACTTCCTAAAGAAGCACGGCAGAAGCTTCTCACGTGGTGG
GAGTTGCATTACAAGTGGCCATATCCTTCTGAGTCAGAGAAGGTAGCGTTGGCGGAATCAACGGG
GTTAGATCAGAAACAAATCAACAATTGGTTCATAAACCAAAGAAAGCGTCACTGGAAACCATCTGA
A
>AtKNAT2
AGTGTCATCAAATCCAAAATCGCTTCTCATCCTTTGTATCCTCGCTTACTCCAAACCTACATCGATT
GCCAAAAGGTGGGAGCGCCTATGGAAATAGCGTGTATATTGGAAGAGATTCAGCGAGAGAACCAT
GTGTACAAGAGAGGAGCTGATCCTGAGCTTGATGAATTCATGGAAACCTACTGTGATATATTGGTT

AAATACAAAACCGATCTTGCGAGGCCGTTCGAC------------GAGGCTACAACTTTCATAAACAAGATT
GAAATGCAGCTTCAGAACTTGTGCACTGGTCCAGCGGCGGATGACAGCCAACAAAGAAGCAATGA
CCGCGATCTGAAGGACCAGCTACTACGCAAATTTGGTAGCCATATCAGTTCATTGAAACTCGAGTT
CTCTAAAAAGAAGAAGAAAGGGAAGCTACCAAGAGAAGCAAGACAAGCGTTGCTCGATTGGTGGA
ATGTTCATAATAAATGGCCTTACCCTACTGAAGGCGACAAAATATCTCTGGCTGAAGAAACAGGTT
TGGATCAAAAACAAATCAACAATTGGTTTATAAACCAAAGGAAACGCCATTGGAAGCCTTCGGAG
>AtKNAT3
GCGAGACACAAGGCGGAGATCCTTTCGCATCCTCTTTACGAGCAGCTTTTGTCGGCGCACGTTGC
TTGTTTGAGAATCGCGACTCCGGTTGATCAGCTTCCGAGAATCGATGCTCAGCTTGCTCAGTCTCA
ACACGTCGTCGCTGGCGACGATAAAGAACTTGACCAGTTCATGACACATTATGTGTTGCTACTGTG
TTCATTTAAAGAGCAATTGCAACAACATGTGCGTGTTCATGCAATGGAAGCTGTGATGGCTTGTTG
GGAGATTGAGCAGTCTCTTCAAAGCTTAACAGGAGTGTCTCCTAGTGAGAGGTCGTTGATGGAAA
GAGTTAGACAAGAACTTAAACATGAACTCAAACAGGGTTACAAGGAGAAGATAGTAGACATAAGAG
AGGAGATATTAAGGAAGAGAAGAGCTGGGAAGTTACCAGGAGATACCACCTCTGTTCTCAAAGCT
TGGTGGCAATCTCATTCCAAATGGCCTTACCCTACTGAGGAAGATAAGGCGAGGTTGGTGCAAGA
GACAGGTTTGCAGCTAAAACAGATAAACAATTGGTTCATCAATCAGAGAAAGAGGAACTGGCATAG
CAATCCA
>AtKNAT4
GCGAGACACAAGGCGGAGATACTGTCTCATCCACTATACGAGCAACTTTTGTCGGCACACGTGGC
GTGCCTGAGGATCGCAACGCCGGTGGATCAGCTTCCGAGGATAGACGCACAGCTTGCTCAGTCT
CAAAACGTCGTGGCTGGCGATGACAAGGAGCTTGACCACTTCATGACGCATTATGTACTATTGCTT
TGCTCTTTCAAAGAACAACTGCAACAGCATGTTCGTGTTCATGCAATGGAAGCTGTTATGGCCTGT
TGGGAGATTGAACAGTCGCTTCAAAGTTTTACAGGAGTATCTCCTAGCGAGAGATCTTTGATGGAA
CGAGTCAGACAAGAACTCAAACATGAACTCAAGCAGGGTTACAAGGAGAAAATTGTGGACATAAG
AGAGGAGATACTGAGGAAGAGAAGAGCTGGAAAATTACCAGGAGACACCACCTCTGTTCTCAAAT
CATGGTGGCAATCTCATTCTAAGTGGCCTTACCCTACTGAGGAAGATAAGGCGAGGTTGGTGCAG
GAGACGGGTTTGCAGCTCAAACAGATAAACAATTGGTTCATCAATCAAAGAAAGAGGAATTGGCAT
AGCAATCCA
>AtKNAT5
GCTAGCTATAAGGCGGCGATTTTAAGACATCCGATGTACGAGCAGCTTCTTGCGGCTCACGTGGC
TTGCCTTAGGGTTGCGACTCCCGTTGACCAGATTCCGAGGATCGATGCTCAGCTCAGTCAGTTGC
ATACCGTCGCCGCGGTTGACAACAAGGAACTTGATCATTTCATGTCACATTATGTTGTCTTGTTATG
TTCATTTAAAGAACAACTCCAACACCACGTTTGTGTCCATGCAATGGAAGCCATTACGGCTTGTTG
GGAGATTGAACAATCACTGCAATCCCTAACTGGAGTTTCTCCAAGAGAGAGATCTTTGATGGAACG
TGTGAAGAAAGAACTGAAGCATGAGCTTAAACAGGGTTTCAAAGAGAAGATTGTGGACATAAGAGA
AGAGATAATGAGGAAGAGAAGGGCGGGAAAGTTGCCAGGAGATACGACTTCTGTACTCAAAGAAT
GGTGGCGAACTCACTCGAAATGGCCATACCCAACTGAGGAAGATAAGGCAAAACTGGTTCAAGAA
ACCGGTTTGCAGTTGAAACAGATCAACAATTGGTTCATCAACCAGAGGAAAAGAAACTGGAACAGC
AACTCT
>AtKNAT6
ACTGTCATCAAAGCTAAAATCGCTTGTCATCCTTCGTATCCTCGCTTACTTCAAGCTTACATCGATT
GCCAAAAGGTCGGAGCACCACCGGAGATAGCGTGTTTACTAGAGGAGATTCAACGGGAGAGTGA
TGTTTATAAGCAAGGAGCTGATCCTGAGCTTGATGAATTTATGGAAACGTACTGCGATATATTAGTG
AAATACAAATCGGATCTAGCAAGACCGTTTGAC------------GAGGCAACGTGTTTCTTGAACAAGATT
GAGATGCAGCTACGGAACCTATGTACTGGTGTCGAGGCAGAGGATGGGAGACAAAGATGTGAAG
ACCGGGACCTCAAAGATAGGTTGCTACGCAAATTTGGAAGCCGTATTAGTACTTTAAAGCTTGAGT
TCTCAAAGAAGAAGAAGAAAGGAAAGTTACCAAGAGAAGCAAGACAAGCTCTTCTTGATTGGTGGA
ATCTCCATTATAAGTGGCCTTACCCTACTGAAGGAGATAAGATAGCATTAGCTGATGCAACGGGGT
TAGACCAAAAACAAATCAACAATTGGTTTATAAACCAAAGGAAACGTCATTGGAAGCCATCAGAG
>AtKNAT7
AGACAGTTGAAAGGTGAGATAGCGACACATCCGATGTATGAGCAATTATTAGCCGCACACGTGGC
GTGTTTAAGAGTAGCTACTCCAATAGATCAACTTCCGATCATTGAAGCTCAGCTTTCTCAATCTCAT
CATCTTCTCCGTCATGATCGTCACGAGCTCGATAATTTCTTGGCACAATATGTAATGGTGTTGTGTA
GCTTCAAAGAACAGCTGCAACAGCACGTGAGGGTTCATGCCGTCGAAGCTGTTATGGCTTGCCGT
GAAATTGAGAACAACCTTCATTCTCTTACAGGAGCAACTTTATCGGAAAGATCCCTTATGGAAAGA
GTCAGACAAGAACTCAAACTAGAACTCAAACAGGGTTTTAAATCGAGAATCGAAGATGTAAGAGAA
GAGATAATGAGGAAGAGAAGGGCTGGGAAATTGCCAGGAGACACAACTACTGTCTTGAAAAATTG

GTGGCAACAACATTGCAAATGGCCTTACCCTACGGAAGATGACAAGGCAAAATTGGTGGAGGAGA
CAGGATTGCAGTTGAAGCAAATCAACAATTGGTTCATTAACCAACGCAAGAGGAATTGGCACAACA
ACTCT
>AtSTM
GCCTCTGTCAAGGCCAAGATCATGGCTCATCCTCACTACCACCGCCTCTTGGCCGCTTATGTCAAT
TGTCAGAAGGTTGGAGCACCACCGGAGGTTGTGGCGAGGCTGGAGGAGGCATGCTCGTCTGCCG
CAGCCGCAGCCGCAGGTGAAGATCCAGGGCTTGATCAATTCATGGAAGCTTACTGTGAAATGCTC
GTTAAGTATGAGCAAGAGCTCTCCAAACCTTTCAAG------------GAAGCTATGGTCTTCCTTCAACGT
GTCGAGTGTCAATTCAAATCCCTCTCTCTATCCTCACCTAATGAATTTGTAGATCCACAAGCTGAGG
ATAGAGAGCTTAAAGGACAGCTCTTGCGCAAGTACAGTGGTTACTTAGGGAGCCTCAAGCAAGAG
TTCATGAAGAAGAGGAAGAAAGGAAAGCTCCCTAAAGAAGCTCGTCAACAACTGCTTGATTGGTG
GAGCCGTCACTACAAATGGCCTTACCCTTCGGAGCAACAAAAGCTCGCCCTTGCGGAATCAACGG
GGCTGGACCAGAAACAGATAAACAATTGGTTCATAAACCAGAGGAAACGGCATTGGAAGCCGTCG
GAG
>CbrYLBK-2043616_streptophytes
AATCGTCTGAAGGCGAGAATAGTATCGCATCCTTTGTATGATCAACTGCTGGACGCGCATGTTTCT
TGCCTCAAGGTGGCAACGCCTCTGGACCAGCTGCCTCTACTGGAGGTCCAACTGGAGCGAGCTG
ACCTTGTTGCGGGGGAAGGGAGGTCGGAGCTGGATCGCTTCATGGCCCAATTTGTGGTCCTGTT
GCGCTCCTTCAAAGTCCAATTGGAGGACCACGTGAAGGTGCATGCAAGGGAGGCAGTGGTGGCG
TGCTGGGAGCTTGAGCAGTCATTGGTGTCAATCACAGGAATGGAGCCGAACGAGCGCTCGATTAT
GGAACGAATGAAAGAGGAACTCAAGATGGAACTCAAGAATGGTTACCGGGACAAGATCTGCGACG
TGCGCGACGAAATCATGCGCAAGCGACGGGCGGGGAAGCTTCCGGGAGATACCACGGCTGTTCT
GAAGACGTGGTGGAATGCACACTCTAAATGGCCATACCCGACGGAAGAGGAGAAAACAAACCTGG
TGCATCAGACAGGTCTGGAACTGAAGCAGATCAACAACTGGTTCATCAATCAGCGCAAGCGCAAT
TGGCACACGTCACCT
>CrGSM1
ccgcagcgcctgacggcggtacagcagcacgaggcgtacccggctgtggtccgcggcaccttggacgtgcgcaaggcgtgcctgccccgctt
cccgctggtggcccgagagcaggccgaggcggagctgcagtccgtcgccacctccggcaacccagggcttgatgaggccatggcgcctac
gtgcgggtcgcggcgctgtacggtgacgaggcggcggcggcggtggcc------------gagtgcgagagcctgatggcggactttgacaacaa
gttgcagctgggcaacctcgccaccaccgctgctgttgctgctgctgggggcgaggcgtcgcagctggctgcgagcattgcggccacgtacggc
agccagctggtgcaggtggctgccagcctggcgcagcggcccaaggtgggcaagctgccgcccgccgccacgcagctgctcaagggctggt
gggacgacaacttcgtgtggccctacccctcggaggaggacaagaagcagctgggtgaggcggcggcgctgaacaacacgcagattaaca
actggttcatcaaccagcgcaagaggcactggcacaagcatttc
>CrKNOX1
GATGTCATTAGATCGAAGATTATGTCTCATCCAACATATCCTCGGCTAGTAATGGCATATGTGAATT
GTCATAAGATTGGAGCTCCTCCGGAGGTCGCCACAAGCTTAGAGGAAATTAGCAAGAAGTACCAG
AGCTTTCGATCGGGAGCGGACCCTGAGCTGGACAATTTCATGGAAACATACTGTAACGTTCTACA
GAAGTACCACGATGAGCTGATGCAGCCTTATAAA------------GAGGCGATGACCTTCTTCAGGAAGAT
AGAACTGCAGCTCAACGCGCTGAGCAAGGGCACCGTGGAGGAGATGATAGACCCACTTGCAGAT
GATCAGAAGGTGAAGGAACAACTCCTGCGTAAGTACAGTGGTTACATATACAAACTTAAGCAGGAG
TTCCTTAAAAAGAAGAAAAAAGGAAAGTTGCCAAAGAATGCACGTGAGAAGCTGCTTGACTGGTGG
AATCAGCATTACAAATGGCCATATCCATCTGAAGCAGAGAAAGCGGCCTTAGCCGAAACCACAGG
ACTCGATCAGAAGCAGATAAATAATTGGTTTATAAACCAGCGGAAGCGGCACTGGAAACCATCGG
AA
>CrKNOX2
GACCTGATCAGAACCAAGATTGTATCTCATCCATCTTACCCTCGGCTTGTGATGGCTTACGTGAAT
TGCTACAAGATTGGAGCTCCAGAAGACGCGGCCTTGATTCTAGAAGAAGTATCAAGAAAATACCAG
GAAATCAGATCGGGAGCGGACCCCGAGCTTGACAACTTCATGGAATTATATTGCAATGTGCTACA
GAGGTATCACGAAGAACTCACACATCCTTACAAG------------GAGGCAATGGCGTTCTTCAAGAAGAT
AGAGCTGCAATTGGATGCTATCAGCAAGGGCAGCCTGGATGAGATGATTGACCCACTTGCAGAGG
ACCAAAAGCTCAAAGAACAGCTGCTTCGAAAGTACAGTGGCTACATATTCAAACTCAAACAGGAAT
TCCTTAAAAAGAAGAAAAAGGGAAAGCTTCCAAGGGAAGCTCGGCAAATGTTGCTTGACTGGTGG
ACCCAGCATTACAAATGGCCATATCCATCTGAGGCTGAAAAGACAGCTTTGGCAGAATCAACAGGA
CTTGACCAAAAGCAGATAAATAACTGGTTCATCAATCAGCGAAAACGGCACTGGAAGCCATCGGA
G
>CrKNOX3
GCGCGGCTCAAAGCAGACATCACCATGCATCCGCTCTATGACCAACTGCTTGCTGCTCACGTAGC

CTGCCTCCGCATAGCCACACCTGTGGATCAACTTCCTCGTATCGACGCGCAGATCGCGCAGGCAT
CGCAAATTGTAGCCGAGGAGAAAGACGAACTCGATCAATTCATGGCTCATTATGTACTCTTGCTCT
GCACATTCAAAGAGCAGCTTCAGCAACATGTGAAGGTTCATGCAATGGAAGCAGTAATGGCATGC
TGGGAGTTGGAGCAATCTTTGCTCACATTAACAGGTGTATCCCCTACAGAACGCACATTGATGGAA
CGTGTCAGGCAGGAGCTGAAGAACGAGCTGAAAAACGGCTACAAAGACAGGATTGTGGATGTCA
GAGAAGAAATCTTAAGGAAACGTCGCGCCGGTAAGCTTCCTGGCGATACTACGTCTGTGCTCAAA
GCATGGTGGCATGCTCATTCAAAATGGCCTTATCCAACGGAAGATGAAAAGGCGCGACTCGTTCA
AGAGACTGGACTTCAATTGAAGCAAATAAATAATTGGTTCATCAACCAGCGGAAGCGTAATTGGCA
TAGTAATCCA
>CspVAZE-2007516_streptophytes
AATCGAATGAAGGCGAAGATCGTATCACACCCTCTGTACGAGCAGCTGCTGGATGCTCACGTATC
CTGCCTCAAGGTGGCGACGCCTCTTGATCAGCTACCGCTTCTGGAGGTCCAGCTCGAAAGGGTG
GACTTAGTCGCTGGAGACGAGAGGCTGGAGTTGGATCGCTTCATGGCACAATTTGTGGTCCTCCT
GCGCTCCTTCAAGGCGCAATTGGAGGACCACGTGAAGGTTCACGCGAGGGAGGCTGTGGTCGCC
TGCTGGGAGCTCGAGCAATCGCTGGTCTCCATCACAGGAATGGAGCCGAACGAGCGCTCGATCA
TGGAGCGCATGAAGGAGGAGCTCAAGATGGAACTCAAGAATGGCTACCGTGACAAGATTTGCGAT
GTGAGGGACGAAATCATGCGCAAGCGGCGTGCGGGGAAGCTTCCCGGGGACACGACGGCTGTC
CTGAAGACGTGGTGGAATGCTCACTCTAAGTGGCCCTACCCCACGGAGGAGGAAAAGACAAACCT
GGTGATGCAGACGGGCTTGGAGCTGAAGCAGATCAACAACTGGTTCATCAACCAGCGCAAACGCA
ACTGGCACACCTCGCCC
>DfoAWOI-2000571_mosses
GCCAGAGAGAAGGCTTTGATTGTGGCTCATCCACTTTATCCAGATTTGCTGATGGCTCACGCTGC
GTGTCTTCGAGTAGGAACCCCTGTGGATCAGCTGCCACAAATTGAAGCCCAATTGGCTCAGGCTC
CTCATGTTACAGCAGAGGAGAAAGCCGAGCTTGACAATTTCATGACTCAATATATCATGCTTCTGT
GTAACTTCAAAGAAACTTTGCAGCAGCATGTTTACACTGATGTAACTGAGGCAATGATGTCCTGCT
GGGAGATAGAGCAGGCGCTTCACGGCCTCACTGGGGTCTCAGCGTCGGAGCGCACACTCATGGA
ACGAGTGCGCCAAGAACTCAAATATGAGCTCAAGCAGGGTTATAGAGCAAGAATTGTTGACGTGC
GAGAGGAAATCCTTCGCAAACGTCGTGCTGGGAAGTTACCGGAAGGCACAACAACGGTCTTGAAG
GCATGGTGGCAAGCGCACTCAAAGTGGCCTTACCCGACGGAAGATGAGAAAGAACGCTTGATTCA
AGAAACAGGGCTGGAGCTGAAGCAAGTGAACAACTGGTTCATCAACCAGCGCAAGCGAAACTGG
CACAGCAACCCT
>EdiCAPN-2003280_ferns
AGAATATTGAAAGGTGAGATCCTGAAGCACCCCCTCTGTGACCAGATCCTGGCGGCTCACATTGC
CTGCCTCCGCATCGCCACACCTGTCGACCACCTCCCTTCCATTGATGCTCAGTTGCTCAATACCCC
ACATGTTATCGCCGACCACAGTGGCGAGCTCGATCAATTCATGACTCACTTTGCACTTCTTCTTTG
GTCTTTCAAAGAACATCTTCAACAACACATGCGGGTTCATGTGATGGAAGCTGTTATGGCCTGTTC
TGAGCTAGAGCAATCCTTGCTAGCATTAACAGGTGTTTCAGCGGCGGAGCGCTCCTTAATGGAAC
GTGTAAGACAGGAGCTAAAAGTTACACTAAAGCAGGGATACAAAGACAGGATAGTCGATGTCAGG
GAAGAAATTCTACGAAAGCGTAGAGCTGGCAAACTTCCGGGTAACACGACGACCGTTTTAAAGGA
CTGGTGGGATGCGCACTCAAAATGGCCATATCCTACTGAGGATGAAAAGGCCAGACTGGTTCAAG
AAACGGGATTGCAATTGAAACAAATAAACAATTGGTTTATAAACCAAAGAAAGAGGAATTGGCATAA
CAACCCT
>EdiCAPN-2006400_ferns
GAAGCATTGAGAGCACAAATCATGTCACATCCACAGTACCCCCAGCTGGTTACAGCATATTTAAAC
TGCCAGAAGGTTGGTGCTCCTCCTGAGATAGTTGTGGGGCTTGATGAGCTTAGTAGAGAATATCC
AAATCACCTTTCTGGATCAGATCCGGACTTGGACCAGTTCATGGAGGATTATTGCAACTTGTTGCA
ACGATATCACGAGGAGCTAACAACGCCATTCAAA------------GAAGCCATGTCCTTCTTTCGAAAGAT
AGAGTTGCAGCTTAACAGCCTAAGTAAAGGAAGCATCTTTCACGATGCTGACCCTCTTGCAGAAGA
AGCAAAACTGAAGGAGAAACTTTTGCGCAAGTATAGTGGATATATCTCTGCACTGAAACATGAGTT
TCTGAAAAAGAAGAAGAAGGGGAAACTTCCAAAAGAGGCGAGGCAGAAACTTCTAGACTGGTGGA
ATCAGCATTACAAGTGGCCATATCCCTCTGAGACAGAGAAAACGTGGCTGGCAGAGAGCACTGGG
CTGGATCAGAAGCAAATAAACAACTGGTTCATCAATCAGAGGAAGCGCCATTGGAAGCCGTCAGA
G
>EdiCAPN-2010377_ferns
GCCAGAATAAAAGCAGAGATCCTGGCTCACCCCCTCTGCGAGCAGCTCCTCGCTGCGCATGTTGC
CTGTCTCCGTATCGCCACACCTGTCAACCAGCTCCCCTCTATCGACAACCAGCTTGCCAATTTCAA
CCAGGTTGTTGCCGACGAAAAGGACGAACTCGATCAGTTCATGACTCATTACGTGCTGCTTCTCTG

GTCTTTTAAAGAACATCTTCAGCAGCACATGCAGGTGCATGTAATGGAAGCTGTTATGGCCTGCTG
CGAGCTAGAGCAATCCTTGCTAGCCATAACAGGTGTTGCACCGACTGAGCGCTCTCTAATGGAGC
GTGTTAGACAGGAGCTAAAAGTTCAACTAAAGAATGGATACAAGGACCGGATTGTTGATGTCAGG
GAAGAAATTCTACGAAAGCGTAGAGCTGGAAAGCTTCCTGGCGACACTACAACAGTTTTGAAGGG
CTGGTGGGATGCACACTCAAAATGGCCATATCCTACTGAGGATGAAAAGTCCCGGCTGGTTCAAG
AAACAGGATTGCATTTGAAACAGATAAACAATTGGTTTATTAACCAAAGAAAGAGGAACTGGCACA
GCAACCCT
>EdiCAPN-2039743_ferns
GCGCGGATGAAGGCGGATATACTGGGGCACCCCCTCTACGAGCAGCTGCTGTCTGCGCACGTGG
GGTGCCTTCGCATTGCCACGCCTGTGCACCACCTCCCGCGCATCGATGCACAGCTTGCTGATGCA
CAGCATGTTGTCGCCCAAGATAAAGACGAACTCGATCAATTCATGACTCATTATGTGTTGCTTCTCT
GCTCTTTTAAAGAACATCTTCAACAACATGTGAGGGTACATGCGATGGAAGCGGTTATGGCCTGTT
GGGAACTGGAGCAATCCTTGCTAGCTTTAACAGGGGTTGCGCCAACAGAGCGTTCATTAATGGAG
CGTGTAAGGCAGGAGCTAAAAACTGAACTAAAGCAGGGATACAAAGAAAGGATTGTTGATGTTAGA
GAAGAAATTCTACGAAAGCGTAGAGCTGGCAAACTTCCTGGCGATAGCACTTCTGTTTTGAAGACT
TGGTGGGATTCGCACTCCAAATGGCCTTATCCCACTGAAGATGAAAAGGCTAGGCTGGTTCAGGA
AACAGGGTTGCAGCTCAAACAAATCAACAACTGGTTTATAAACCAAAGGAAGAGGAATTGGCATAG
CAACCCC
>LdePQTO-2010278_lycophytes
GTGAGATTGAAAGGAGAGATCGTGTCGCATCCTTTGTACAACCAGTTGGTGACTGCACATGTCGA
GTGTTTGCGCACTGCCACACCAGTGGACCAGCTGCCAAAGATCGATGCCCGATTGGCTAATGCCG
GCCACATAACAGCGGAAGACAGGGATGAGTTGAATGGGTTCATGGCCAACTATGTTCTTCTTCTCC
AATCTTTTAAGGATCAATTGCAACGACATGTACGAGTCCATGCAATGGAAGCTGTTATTGCCTGTT
GGAAGTTGGAGCAGTCCCTTTTGAATCTAACAGGGGCTACACCTACTGAGCGCACTCTCATGGAG
CGAGTGCGCCATGAATTGAAGCTTGAATTGAAGCAGGGTTATAAGGCAAGGATCAATGATGTAAG
GGAAGAAATTTTGCGCAAGCGTCGTGCTGGCAAGTTGCCAGGCGATACAACATCAGTACTTAAAT
CTTGGTGGTTTGCACACTCAAAGTGGCCGTATCCTTCGGAAGAAGAAAAGGCAAGGCTTGTCCAG
GAGACTGGCTTAGAGTTGAAACAAATCAACAACTGGTTCATCAATCAACGAAAGAGAAACTGGCAT
AACAATGCA
>LdePQTO-2010435_lycophytes
GATGAGATTGAGGCGGCGATTCTCTCTCATCCTCATTATTCGAGTCTCGTTGCAGCTCATATGAAT
TGTCACAAGGTAGGAGCTTCCCCAGAAGTTGTATCCCATATCGATAGCCTCATCAGGAAATTTCAG
GAGAGGCAACCTGGTGTTGATCCAGAGCTCGATCAGTTTATGTACACCTACTGCAATATGCTTCTG
AACTATCAACGAGAGATCACCAGAACGTTTGAG------------GAAGCGATGGCTTGTTGTCAGAAGCTA
GAAACTCAGCTGAATATCATCTCTACCGGAAGCATCGACTTAGAAATTGACCCTTTCGCAGAAGAG
AAGGAGATAAAAGAACATCTTATGCGAAAGTACAGTGGCTACATTGGGAGCTTGAAGCAAGAGTTT
TCGAGAAAGAAGAAGAAAGGGAAGCTTCCAAGAGAGTCCAGGCAACAGCTACTGAACTGGTGGTC
TATGCATATTAAATGGCCTTACCCCTCGGAGGTGGAGAAGGCAAGCCTAGCAGAGTCTACTGGAC
TGGATCAAAAACAAATCAACAACTGGTTTATAAATCAGAGGAAGAGGCACTGGAATCCTTCGGAT
>LdePQTO-2081329_lycophytes
AGCGAAATCAGGGCTGCCATCCTTGCACATCCTCACTATCCTCGGTTGGTTGCAGCCCACATGAA
CTGCCACAAGGTGGCAGCTTCTTCGGAAGTTGTTTCTCAGATTGACGATCTCATACGGGAATTCCA
TGAGAGGCAGTTTGGAGCAAATCCAGAACTCGATCACTTCATGGTTTCCTATTGCAACATGCTGAT
GAGCTACGAGCAAGAGGTTAGCAGGACATACAAG------------GATGCGATGTCTTTCTGCAGGAAAT
TGGAACAGCAGCTCAGTGTTTTCTCAACTGGAAGTATCGATTTGGAAATTGATCCTTTAGCAGAAG
AGAAAGAATTAAAGGAACAACTGATGCGCAGATACAGTGGGCGTATTGGTAGCTTAAAGCAGGAA
TTTATGAAGAAAAAGAAAAAAGGGAAGCTTCCAAAAGAGTCCAGGCAACAACTCCTAGACTGGTGG
TCAGGGCATATAAAATGGCCTTATCCCTCGGAGGAAGAGAAGGCCAGCTTAGCGGAATCTACAGG
CTTGGGCCAGAAACAAATCAACAACTGGTTTATAAACCAGAGGAAGAGGCACTGGAGGCCTGCGG
AA
>LdePQTO-2081967_lycophytes
GTGGGATTGAAAGCGGACATTGTGTCTCACCCTCTCTATGAACAGCTTCTGGAAGCTCATGTGGC
ATGCTTACGCATTGCAACTCCTGTGGATCATCTGGCAAAGATAGACGGCCAGCTTGTCCACTGTCA
TCACATCACAGCCAACAACAAGGATGAGCTTGATCAATTTATGACACAATACCTATTATTGCTCCGG
TCGTTTAAGGAAAGGTTGCAACATCATGTGTGTGTGCATGCGAAAGAAGCTGTCATTGCTTGCCGA
GAGCTCGAATATTCACTTTTGAGTTTAACTGGTGCATCCCCAACTGAGAGGACACTCATGGAGCGG
GTTCGACAAGAGCTTAAGCATGAACTTAAACAGGGATATAGAGATAGAATAGCGGATGTGAGAGA

GGAGATACTTCGGAAGCGCCGTGCTGGAAAATTACCTGGGGATACAACGTCTGTCCTAAAAGCCT
GGTGGCAAGCTCATTCAAAATGGCCTTATCCCACGGAAGATGAGAAGGCTCGGTTAGTGCAAGAG
ACCGGCCTTGAAATGAAGCAAATCAACAATTGGTTCATAAATCAAAGGAAACGAAACTGGCATCAC
CACCCT
>LdePQTO-2083351_lycophytes
GTAAGACGGAAAGCTGAAATTGTATCACATCCACTGTATGATCAATTATTGACGGCACATGTTGCA
TGCTTGAGAACCGCAACACCAGTTGATCAACTATCGAAAATTGACGCACAATTGGCACATGCCAGC
CATGTAGCGGCAGAAGATAAGGATGAGCTAAATCAGTTCATGGCTCAATATGTGATGCTTCTCCAC
TCCTTCAAGGAGCAATTGCAACAACATGTGCGGGTTCATGCTATGGAGGCTGTTGTCGCATGCTG
GGAGATGGAGCAGTCACTTCTAAATTTGACAGGGGTGACCCCTACAGAGAGAACTCTTATGGAGC
GAGTACGACATGAGCTGAAGCTTGAATTGAAACAAGGATATCGAGATCGGATCAATGATGTTAGG
GAGGAGATCCTTCGAAAGCGTCGTGCTGGCAAGTTACCTGGAGACACTACATCAGTACTCAAGGC
CTGGTGGCATGCACACGCAAAGTGGCCTTACCCATCGGAAGATGAAAAAGCAAGGTTAGTGCAAG
AGACTGGACTAGAACTGAAACAGATCAACAATTGGTTCATCAATCAGCGAAAGAGAAATTGGACCA
ACAACCCA
>LspCHJJ-2136614_liverworts
ATCAAACTTAAAGCGAAGGCCCTCACGCACCCGTTGTTTCCTCAGATGCTCGCCGCACATGTTGC
CTGTTTGAGAGCCTGCACGCCGATCGACCAGCTTTCAACTATTGACGAGCATCTCAGTAATGCTCA
AGCTGTGATAGATGCTGACATGGACGATCTGGACTTCTCCATCAGGACCTACACGATGATGGTGG
TGTATCTTCATTTCGAGTTACAGCATCATATACGTATGCATACTGCAGAAGCAATGCTGGGATGCT
GGGACCTGGAGCAGAAGCTTCACAATCTAACCGGCGTCCCTCCGTCTGAGCGCTCTCTGATGGA
GCGCGTTCGTCGAGATCTGAAGGCCGAACTCGAGGAGGGCTATCGGAAAAAGATTATCGAGGTG
AGGGAGGAGATTCTCCGCAAACGCCGAGCTGGAAAGCTACCTGAAGGGACAACGTCAATCTTAAA
ATCCTGGTGGAACGATCATGCAAAGTGGCCCTACCCTACCGAGGACGAGAAGGAATATTTGATCC
AAGAGACAGGGCTTGAATTGAAGCAGGTCAACAACTGGTTCATTAATCAACGCAAGAGAAACTGG
CACAGCAACCCT
>AXG93_606s1230
TTAGACCTTCAGTGTCTCATCGTGCATCATCCTGACCTCCCTAAGATAATTGTGGCAAGCCTTGCC
TGCTATAAGATAAGGGCTGATGAAGAGGAGAAAAAGCAGCTGGACGAGGTGGCCAACTTATCTCT
TTCCAAGCTCCCAGGTAAAGACCCACGTCTCGACTTATGCTTGGATAAGTATCTGAAAGCCCTCGA
TGAGTTGGAGAAACACATTTCGAAGCTGGTCACG------------TCAGCCGACACTCACTGTGTTCAGAT
GAAACAGAACATTCTTATACAGTGTCCAGAACTTGGTCCCCAGCGATCTCTCCCCATGTTGACAGA
TCAAGAATGGAGAGGAGCAATCTTAGCTGCATATAATCCAAGTCTTCTCAGGTTGAAAACGAGGCA
TCTGAAAGAAGGCCGCAGAAACAAGCTGCCCGATCCGGCTCGACGTATTTTGCTCAATTGGTGGA
CCGAACATCAGGGGAAGCCTTACCCGTCGGAGGAGGAGAAACAAATTCTGGCCAAGTGCTCGGG
CCTTGAGATTGATCAGATCAATAACTGGTTCATCAATCAGAGGAAAAGACACTGCTGCTCTGCGTT
C
>AXG93_3769s1000
GCCAGATTCAGGGCTAGACTCATTACCCATCCATTATTTCCTCAACTCCTCAAGGCACATGTAGAC
TGCATCCGTGTGATGACTCCAGCATCCAAGATACGTGACATCAATCTGCAGCTGGTTCAGGCTCAA
AAGTTTTTCGAACCTGATGATTTTGAATTCGACTACTTCCTGGAAAATTTCCTGGTTCGGATGAAAC
ATTTCCAATCGAAATTGGAGATGCAGCTCAAGGTTCACACCACTTTGGCTTACAGCAAATTATGGC
GCATCGAGCGGGTACTCGTCGACATGACAGGTCAGCGCATTCCAGATCGTGCTGTCATGGAGAG
CATCAGGCGAGAGTTGATTGCATCACTTTTTGAAAAGTACAAGAAGAAGATCAAAGAAGTGAGAGA
TGAGATTTTGAGAAAACGAAGAGCGGGCAAATTGCCAGACGGAACCACACAAATACTCAAGGCGT
GGTGGGATGCTCATTCAAAGTGGCCTTATCCAACGGAAGATGAGAAAGAAGTTCTTCTCAAAACGA
CGGGATTGGAACTGAAACAAATCAACAACTGGTTCATAAATCAAAGGAAAAGAAATTGGCACAACA
ATCCA
>Mpu_69285
------------------ATGCGCGTCCACACGATGTACCCCGCGCTTCTTCGCGCGTACTTCGCGTGCCGAAG
CGCGGGCGCGGACCGCGCGACGGAGCTCGCGCTGCGGCGGCGGAGAGACGCGCTCCTCGAAG
ACGCGCGCCAGGGCGGCGAGGAGATGGACGACTTCATCGCGCGATGCACCGCGGAGTTGAGAG
CGTATTCCGTGGAGTTGAACGCCATCGCGCGG------------GAGAGCGAGGACGCGTGCGCGGAGTT
TGAACGACGCGTCGCTACGATCACCGCCTCCGCGGCGCCGTCTCTCACCCACGTGGACACGCGA
GAGGACGACCTGCGCAAGTCTCTCAAGCGGAAGTACGCGACGTCGATATCGTCCCTTCGAGACG
AGTTCCTCCGCAAGAGGAAGAAAGGCAAGCTCCCGACGGACGCCACGGAGGCGCTGAAAAAATG
GTGGAGCGACAACGTCGTATGGCCGTATCCGTCCGAAGACGACAAGCGCGCGCTGTCAAAGTCT

ACCAACCTCAGCGCGACGCAGATCAACAACTGGTTCATCAACCAGCGGAAGCGACACTGGCACAA
GCTGTTT
>Msp._62285
GGCGCCATGCGCGCGGCGGTGCGCGCGAACCCGCGGTACCCGAAGCTCCTCGACGCGTACTTC
GCGTGCCGCCGGGTGGGCGCGGACGCGACGTCCAAGGCGTCGCTCGCCCGACGCAGGCGACA
GTTGCTGCGAGAAGCCACCCGGTACGGCGCCGAGCTCGACGAGTTCATGGACAACGTCACCGAC
GAGCTCACCGCGTACGCCGAGGAGCTCGGCGCCTGTTTCGAC------------GAGGTGGACGCGGCGT
GTCGAGAGGCGGAGGCTCGCGTGGCGGCGACGGCGGCTAAAAAATTGGCGAAGGAGTCCATCC
CCGACACCCGCGAAGACGACCTGCGCAAGTCTCTGAAGCGCAAGTACGCCTCCTCCATCCTCGC
GCTCAAGGATGAGTTTCTGAAGAAGACGAAGAAGGGAAAGCTGCCGTCCTCCGCGACGAAAACGT
TGAAGGAGTGGTGGCTGGCCAACCTGCTGTGGCCTTACCCGAGCGAAGACGCGAAAAGAGCGTT
GATGAAACTCGCGGGCCTCAACCAGACGCAGATCAACAACTGGTTCATCAACCAGCGGAAGCGG
CACTGGCACAGGCTGTTC
>MspZRMT-2001684_streptophytes
GTTGAAGCAAAAAAATCTGTTCTCACACACCCGTTATATTCTGAGCTCATTAAGACTTATTTTGATT
GCAAGAAGGTAATTACCCCATCAGAGCTAGAGGAGCTGAACAGCCTCATGCGTCGTTTGATTCAG
CTCGCACCACCAAACCCAAGTCCAGAAATGGATTCCTTCATGGAAGAATATGTGGGGCTTTTGAAG
TTTTACTCAAACGAGCTTAGTAAGACGTACAAG------------GAAGCCTCAATTGTGTGCAATAATTTTG
AGGAGAAATTGAAGACCCTTTCGCTGTCCTTCACAAGCAGCGATGCAAACCCCAATACCGACGAG-
--GCGCTGAAAGAAGAGCTGAAGAAAAGGTACGCGTCGTACCTGTGTTCCCTCAAACAAGACTACT
TGAAGAAGAAGAAGAAAGGGAAGTTGCCCAAGGGAGCAACGGAGACTCTTCTAGAGTGGTGGCA
GGCTCATATCAAGTGGCCGTACCCAACAGAGGCGGAAAAAGTACAGCTGGCGGAGGGCACAGGT
TTGGATTCGAAACAAATCAACAATTGGTTCATTAATCAGCGGAAACGACACTGGTCGCACGGTGAT
>Olu_5313
GAGGAGATGACGCTCGCGGTGCGCGCTCATCCCATGTATCCGCGGCTTGTGGAAGCGTATTACG
AGTGCCGGCAAATTGGCGCTGAGGGTGATGTGTTGGAAGCGCTCGATCGAGAGCGCGATGCGAT
GTTGTACTCGGTTCAGGTACCGCAACGAGATTTGGACCGGTTCATGCGAGAGTGCACGCACGAAC
TTGAATCGTACGTCAAGGAGCTACACGCGCTATACGAA------------GACGCGAAGTCGTGCTGTCGA
GAACTCGAGACGCGCGCGCGGAAAGTGCGTTCGGATGTGGTGTCTAACTCGCAACAGCGTCGCG
ACCACGAGGAACGACTCAGAGAAGCCCTTAAACGTAAGTACGCGAGCAGTATTATGACGCTCAAG
TCGGAGTTCATGCGAAAACGAAAGAAAGGCAAGCTTCCAGACCAATCCACAGAGGTGCTAAAGAA
CTGGTGGTCCGAAAACATCGTGTGGCCGTACCCGACTGAAGATGATAAGCGCGAACTCATCGCGC
AAACAAAACTCGACGCGACGCAGGTCAATAACTGGTTCATTAACTTTCGAAAGCGCCACTGG--------
----
>OsHOS58
GAGAGGGAGAAGGCGGCCGTCGCGGCGCACCCGCTCTACGAGCGCCTGCTCGAGGCGCACGTC
GCCTGCCTCCGCGTCGCCACCCCCGTGGACCAGCTCCCCCGCATCGACGCGCAGATCGCCGCG
CGCCCGCCTCCCCTCGCATCCGGTGGCGAGGAGCTCGACCTCTTCATGACACATTATGTGTTGCT
TCTTTGCTCATTCAAGGAACAACTTCAGCAGCATGTGCGTGTTCATGCAATGGAAGCAGTGATGGG
GTGCTGGGAACTTGAACAGTCTTTACAAAGTCTAACAGGGGCATCTCCTGGTGAAAGATCCTTGGT
TGAGCGTGTACGGCATGAGCTGAAGAACGAGCTTAAGCAGGGGTACAAAGAAAAGCTTGTGGATA
TTAGGGAAGAGATCCTTCGCAAGCGGAGAGCTGGTAAACTTCCTGGTGATACTGCATCTATACTAA
AAGCTTGGTGGCAAGCTCATTCTAAGTGGCCATACCCAACTGAAGACGACAAGGCCCGCCTGGTG
CAGGAAACGGGGTTACAACTGAAGCAAATCAACAATTGGTTCATTAACCAACGCAAACGAAACTGG
CATAGCAACCCA
>OsHOS59
GAGCGGGAGAAGGCCGCCATCGCGGCGCACCCGCTGTACGAGCGGCTGCTGGAGGCGCACGTC
GCGTGCCTCCGCGTCGCCACCCCCGTCGACCAGCTGCCCCGCATCGACGCGCAGATTGCGGCG
CGCCCCCCGCCGCTGGCCTCCGGCGGCGAGGAGCTCGACCTCTTCATGACCCATTATGTATTGC
TCCTTTGTTCGTTCAAGGAACAACTACAGCAACATGTGCGTGTTCATGCAATGGAAGCAGTAATGG
CTTGCTGGGAACTTGAACAAACTTTACAGAGCCTTACAGGGGCATCTCCTGGCGAGAGATCATTA
GTTGAGCGTGTACGGCAAGAGCTGAAACATGAGCTTAAACAGGGGTACAGAGAAAAGCTTGTGGA
CATTAGGGAAGAGATACTCCGAAAGCGAAGAGCTGGAAAACTCCCAGGAGATACAGCGTCTACTT
TGAAAGCATGGTGGCAGGCTCACTCTAAATGGCCATACCCAACTGAGGAGGACAAGGCTCGCTTG
GTGCAGGAAACAGGGTTGCAACTAAAACAGATCAATAATTGGTTTATCAACCAACGTAAACGGAAC
TGGCACAGCAATCCT
>OsHOS66

CAGCTGCTCAAGGGGGAGATCGCGGTGCACCCGCTGTGCGAGCAGCTGGTGGCGGCGCACGTG
GGCTGCCTGCGCGTGGCGACGCCCATCGACCACCTCCCGCTGATCGACGCGCAGCTCGCGCAG
TCCAGCGGCCTCCTCCACCACGACAAGCAGGAGCTCGACTCCTTCCTCGCGCAGTACATGATGCT
GCTGTGCTCGTTCAGGGAGCAGCTGCAGCAGCACGTCCGGGTGCACGCCGTGGAGGCCGTCAT
GGCGTGCCGCGAGATCGAGCAATCCTTGCAGGACCTAACCGGTGCAACTTTGTCCGAGCGATCC
CTCATGGAGCGGGTCAGGCAAGAGCTCAAGATTGAGCTCAAGCAGGGCTTCAAGTCAAGGATTGA
GGATGTGAGAGAAGAAATATTGAGGAAGAGGAGGGCAGGAAAGTTGCCTGGGGACACCACCACC
ATACTCAAGCAATGGTGGCAGCAACACTCCAAGTGGCCATACCCCACGGAGGATGATAAGGCAAA
GCTTGTTGAAGAGACTGGCCTGCAGCTCAAGCAAATCAACAACTGGTTCATCAATCAGAGAAAGC
GTAACTGGCACAACAACTCA
>OsOSH1
GAGGCCATCAAGGCCAAGATCATCTCCCACCCCCACTACTCCTCCCTCCTCGCCGCCTACCTCGA
CTGCCAGAAGGTTGGGGCGCCGCCGGAGGTGGCGGCGAGGCTGACGGCGGTGGCGCAGGACC
TGGAGCTTCGCCAGCGCGCCACGGAGCCGGAGCTGGACCAGTTCATGGAGGCGTACCACGAGAT
GCTGGTGAAGTACAGGGAGGAGCTGACGAGGCCGCTGCAG------------GAGGCCATGGAGTTCTTG
AGGAGGGTGGAGACGCAGCTCAACACGCTCTCCATCTCCGGCAGACTACCTGAGATTGATGCACA
TGGTGTGGATCAGGAGCTCAAGCACCATTTGCTGAAGAAGTACAGTGGATACCTGAGCTCCCTGA
AGCAAGAACTGTCAAAGAAGAAGAAAAAAGGGAAGCTCCCCAAGGATGCTCGTCAACAGCTCCTC
AACTGGTGGGAGCTGCACTACAAATGGCCTTACCCCTCGGAGAGTCAGAAGGTGGCACTGGCGG
AGTCGACGGGGCTGGACCTGAAGCAGATCAACAACTGGTTCATCAACCAGAGGAAGCGGCACTG
GAAGCCCTCCGAC
>OsOSH10
GATGCAATCAAAGCCAAGATCATGTCGCACCCCCTCTACCCGGCTCTCCTCAGAGCCTTCATAGAT
TGCCAGAAGGTCGGAGCTCCGCCGGAGGTCGTCGGCCGGCTTTCCGCCCTCGCCGGCGAGCTC
GACTCGCGTGCAGAATCGTCAGACCCGGAGCTCGACGAGTTTATGGAAACCTACATTGATATGCT
GGTGAGCTACAGGCAGGAGCTGACAAGACCAATTCAA------------GAGGCCGACCAGTTCTTCAGAA
ACATGGAGGCACAGATCGACTCGTTTACACTAGATGACAACCCGGAGATCACCAGCCCCTGCGCG
GAGGACAAGGAGCTGAAGAGCCACCTCCTGAACAAGTACAGCGGCTACCTGAGCAGCCTATGGA
GGGAGCTCTCCAAGAAGAAGAAGAAAGGCAAGCTGCCCCGGGACGCTCGCCAGAAGCTCCTCCA
CTGGTGGCAGCTTCACTACCGATGGCCCTACCCATCGGAGCTGGAGAAGGCGGCGCTGGCGGA
GTCGACGGGGCTGGACGCGAAGCAGATCAACAACTGGTTCATCAACCAGCGGAAGCGCCACTGG
AAGCCGACGCCG
>OsOSH15
GAGTCCATCAAGGCCAAGATCATGGCGCACCCCCAGTACTCCGCCCTCCTCGCAGCCTACCTCGA
CTGCCAGAAAGTCGGAGCGCCGCCGGAGGTGCTGGAGAGGCTGACCGCCACGGCGGCAAAGCT
GGACGCCCGCCCTCCCGCGCGCGACCCGGAGCTCGACCAGTTCATGGAGGCGTACTGCAACAT
GCTGGCCAAGTACAGGGAGGAGCTGACGCGGCCGATCGAC------------GAGGCCATGGAGTTCCTC
AAGAGGGTGGAGTCGCAGCTCGACACCATCGCCGGCGGCGCCCATCCGCCTGAGATCGACCCG
CGCGCTGAGGATAAGGAGCTCAAGTTTCAGCTTCTGAAGAAGTACAGTGGCTACTTGAGCAGCCT
AAGGCAAGAATTTTCCAAGAAAAAGAAGAAAGGAAAGCTGCCTAAGGAGGCCAGGCAGAAGCTGC
TTCACTGGTGGGAGCTGCACTACAAGTGGCCTTACCCCTCAGAGACGGAGAAGATTGCGCTTGCG
GAATCGACAGGACTAGATCAGAAGCAGATCAACAACTGGTTCATCAACCAGAGGAAACGGCACTG
GAAGCCATCGGAG
>OsOSH3
GACCCTGTCAAGGCCAGGATCGTCTCCCACCCTCGCTACCACCGCCTCCTCGCCGCCTTCCTCGA
CTGCCACAAGGTTGGCTGCCCGGCGGAGGCGGCCGAGGAGATCGCGGCGGCGGCGCGGGTGC
GGGAGGCGCGGCAGCGGCCGGAGGACCCGGAGCTCGACCAGTTCATGGAGGATTACTGCAAGC
TGCTCGTGGAGTGTAAGGAGGAGCTCAGCCGGCCGCTCCAG------------GAGGCCGAGGAGTTCCT
CAGGACGGTGGAGTCAGAGCTCAACTCCATCAATTCTGGGCCACCCCTCGGCATCATCGACCCTC
GCTCCGACGACAAAGCGCTGAAGAGGCACCTGCTGAGGAAGTACAGCGGCTACCTGGGAGGCCT
GAGGAAGGAGCTGTCGAAGAAGAGGAAGAAAGGGAAGCTCCCCAAGGAGGCGAGGCAGAAGCT
GCTCACCTGGTGGGAGCTCCATTACCGGTGGCCGTACCCGTCGGAGATGGAGAAGATCGCCCTC
GCCGAGTCGACGGGGCTGGAGCAGAAGCAGATCAACAACTGGTTCATCAACCAGCGGAAGCGCC
ACTGGAAGCCCACGGAG
>OsOSH45
gcgcggtgcaaggcggagatactggcgcacccgctgtacgagcagctgctgtcggcgcacgtggcgtgcctccgcatcgccaccccggtcga
ccagctcccccgcatcgacgcgcagctcgcgcagtcgcagggcgtcgtcgccgacgacggccgcgagctcgaccagttcatgacgcactac

gtcttgctattatgttcgtttaaagagcagctacagcagcatgtccgtgttcatgctatggaagcagtaatggcatgctgggaacttgaacaaaacct
tcagagtttgacaggtgcctcgcccagcgagcgatccctaatggagcgtgtccgtcaagagctaaagcatgaactaaagcagggttacaaaga
gaagctgattgacataagagaagaaattcttcgcaagcgaagagcgggaaagctaccgggagacacgacttctacgttaaaagcttggtggc
agtctcatgcaaagtggccatatccaacagaggaagacaaggcacggctagtccaagaaacagggctacagctaaagcagatcaacaact
ggttcatcaaccaaaggaaaagaaactggcacagtaaccca
>OsOSH6
GAGCTCATGAAGGCTCAGATCGCCGGTCACCCGCGCTACCCGACCCTCCTCTCCGCCTACATCG
AGTGTCGCAAGGTGGGAGCGCCTCCGGAGGTGGCGTCGCTGCTGAAGGAGATCGGCCGGGAGA
GGCGCGCCGGCGGCGGCGGCGTCGACCCCGAGCTCGACGAGTTCATGGAGGCGTACTGCCGG
GTGCTGGTGCGCTACAAGGAGGAGCTGTCCCGGCCGTTCGAC------------GAGGCGGCGTCTTTCC
TCAGCAGCATCCAGACGCAGCTCAGCAACCTCTGCAGCGGCGCCACCGGGCAAGAGCAAAGCTC
CCGCTTAGCTGACCACGAACTCAAGGAAATGCTTCTGAAGAAGTACAGTGGCTGCCTCAGCCGGC
TCCGGTCCGAGTTCCTGAAGAAGCGGAAGAAAGGGAAGCTACCAAAGGACGCACGGTCGGCACT
GCTTGAATGGTGGAACACACACTACCGCTGGCCATATCCCACGGAAGAAGATAAGCTGAGGCTTG
CAGCGAGGACAGGTCTTGACCCAAAGCAGATCAACAATTGGTTCATCAACCAGAGGAAGAGGCAC
TGGAAGCCATCGGAC
>OsOSH71
gagctgatgaaggcgcagatcgccggccacccgagctacccttccctcctctccgcctacatcgaatgccgcaaggttggtgcgccgccggag
gtgaccacgctgctggaggagatcggccgggaggggcgcggcggcggcggcggcctcgatccggagctcgacgagttcatggagacgtac
tgccgggtgctggagcggtacaaggaagaactcacacggccgttcgac------------gaggcggcctccttcctcaccggcatccacacccag
ctcgcctccctctgtggcggcgcgccggggcaagaacacagctcccgcttggctgaccatgagctcaaagagatgttgctcaagaagtacagt
ggctgcctcagccgcctccggtccgagttcttgaagaagaggaagaaagggaaactaccgaaagacgcgcgatcggcacttatggactggtg
gaacacacattaccgttggccttaccccacggaagaggataaggtgaggctggccgcgatgactggcctcgacccgaagcagatcaacaact
ggttcatcaaccagaggaagaggcactggaagccatcggag
>PcaRXRQ-2002267_hornworts
GCAAAGAGGAAAGGAGACATTGTTTCTCATCCACTCTTCCCGGAGCTCCTCTCAGCTCATGTGGCT
TGTCTGCGCATCGCGACACCTGTTGACCAGCTCCCGACAATAGATGCACAATTGGCACAGTCACA
CCTTGTCATTTCCTCCGAGAAGGAGGAGTTGGAGCATTACATGGCAGAGAGTGTCATGCTTCTCC
GGTCCTTCAAAGATCAACTGCAGCAGCATGTTCGAGTGCACGCCATGGAAGCTGTGATGGCTTGC
TTCGACATTGAGAAGCAACTTGAAGATTTGACTGGCGTTCGAGTTACAGAGCGCACACTTATGGAA
CGCGTGCGGCAGGAGCTGAAATCTGAGCTCAAACAGGGTTACAGAGCCAAGATCATCGACGTGA
GGGAGGAGATATTGCGCAAGCGCCGGGCCGGAAAATTGCCAGGTGATACCACTACGGTGCTGAA
AACCTGGTGGCATGCGCATGCTAAGTGGCCCTATCCTACGGAGGACGAGAAGGCGCGGTTGGTC
CAGGAAACGGGCCTGGAGCTGAAACAGATAAATAACTGGTTCATCAACCAACGCAAACGCAATTG
GCATGCCAACCCC
>PcaRXRQ-2002409_hornworts
GCAAAGAGAAAAGCGGATATTGTAGCGCATCCTCTGTTTCCCGAGCTTCTGTCTGCTCATGTCGCT
TGCTTGCGCATCGCGACACCTGTGGACCAGCTGGGAACCATCGATGCACAGCTGTCGCAGTCAC
ACCTTGTAATCAGCCCAGACAAGGAGGAGCTGGAGCAATATATGGCAGACAACGTCATGCTGCTC
CGTTCTTTCAAGGACCAGCTGCAGCAACACGTTCGAGTACATGCCATGGAGGCTGTAATGTCTTG
CTACGACCTGGAGAAGCAACTTGAAGAGCTGACTGGCGTTCGGGCAACAGAGCGCACTCTCATG
GAACGCGTGCGGCAGGAGCTTAAGGCAGAGCTCAAACAGGGATACAAAGCCAAGATCATAGATGT
GAGGGAGGAGATACTGCGAAAGCGTCGAGCCGGCAAGCTGCCAGGTGATACAACTACGGTGCTG
AAAACATGGTGGCATGCGCATGCTAAGTGGCCCTACCCTACGGAAGACGAGAAGGCACGCCTAG
TGCAGGAGACTGGTCTGGAGCTGAAGCAAATCAACAACTGGTTCATCAACCAGCGCAAACGCAAC
TGGCATGCTAATCCT
>PpMKN1
GCCAGAGACAAGTTTCTGATTGTTGCTCATCCACTATATCCAGATTTATTGAATGCACACGCTTCTT
GTCTTCGGGTGGGGACCCCGGTAGATCAGCTTCCACATATTGAGGCGCAGTTAACTCAAGCTCGA
CATGTTACAAGCGATGAAAAAACAGAACTTGATCAATTCATGGCCCAGTATATCATGCTCCTTTGTT
CGTTCAAAGATCACCTTCAACAACACGTATATTATGATGTTACTGAGGCCATGATGTCTTGTTGGGA
GTTGGAGCAAGCACTACACAATCTCACTGGAGTCTCTGCATCGGAGAGAACTCTTATGGAACGTG
TTCGCCAAGAACTCAAGTACGAGCTGAAGCAGGGATACAGAGCAAGAATTGTTGATGTGCGAGAG
GAGATCCTACGCAAACGGCGTGCTGGAAAGCTGCCTGAGGGAACTACAACAGTCTTGAAGGCTTG
GTGGCAAGCACATTCAAAGTGGCCATATCCCACGGAGGATGAAAAGGAGCGGCTGATTCAGGAG
ACAGGTTTGGAATTGAAGCAAGTGAATAATTGGTTCATAAACCAACGCAAGCGGAACTGGCACAG
CAATCCA

>PpMKN2
CTAGAGCTTCGGGCTGCTATCATCGACCACCCTTTCTATCCGGAGATGGTCCTAGCCCACGTTAG
GGTCTTCAAGATTGGAGCACCCAGACGGTTACGCCGCAAACTGGACGAACTAGCAAAGAAGTTTC
AACGTTTCCAAGATGGCTCTGATCCTGAGCTCGATCACTTCATGAGATCATACGTGGGCGTGCTTA
CGAAGTTCGCGGAGGACTTGGAAGAACCTTTCAAT------------AAGTTTATCCAGTTCACAGATAACA
CCTCCAAGGCTCTGGAAGAAATCTGCGGCCACTACGTTAGCGTCATTGTTGACCCAATGGCATCG
GACGAAGATATCAAGAAAGCCCTGAGGAAGAAATATGGTCGCCACATCGGAGAGCTCAAGGCTGA
ATTCAACCGGGTGCGCAAGAAAGGGAAGTTGCCAACGAGTGCTCGCACCATCTTGAAGGACTGGT
TCAATCGCCATTCACACTGGCCTTACCCTTCAGAGATGGAGAAACAATACCTGCAAAGAATCTGCG
GCTTGAATTTGAAGCAAATCAACAACTGGTTCATTAATGAGCGCAAGAGGCACTGGAGCTGCGAA
GGT
>PpMKN4
-------------------------------AGTTATCCAGACCATGAAATTTCTTTTGTG---TTTCACAGGATTGGTGCGC
CGAAAGGTCTTCTTATCAAACTAGACGAGATGGAGAAGAAGTTCCAACGATTCCAATACGGCCAG
GATCCTTCACTTGATTTCTTCATGAGATCGTACATAGACTTGCTAACAAAATTTCGAGAGGATTTGG
AGAACCCTTACAAC------------AAATTCGCGCAGTATAAGGATAAGGTTACCAAGGATTTGGAAGATC
TTTGCGGCCACTATATTAGCATAGTAATCGATCCTGACGCAGCGGACGAAGAACTCAAGAAAATGC
TGAGGTTGAAATACGGG---AAGCTGCCCACCAATGCTCGTCAG
ATCTTGAAGGACTGGTTTAGCCGTCATTCTTACTGGCCTTATCCTTCGGAAATGGAAAAAGCATAC
CTGCAGCGACTCTGTGGCTTAAATTTGAAGCAAATCAACAACTGGTTCATCAATGAGCGCAAGCGA
CACTGGAGCTGCGAGGGT
>PpMKN5
CCAGAACTACGGGCTGCGATCATCGATCATCCTTTCTATCCAGAGATGGTTCTTGCCCATGTCAGA
GTCTTTAAGATCGGTGCCCCTAGACGTCTGATCAACAAATTAGATGACCTGACGAGGAAGTTTCAA
CAGTACCAGAACGGCACCGACCCTGCACTCGATCACTTCATGAGGTCATACGTGGACATGCTCAC
TAAATTCGCTGAGGACTTGGAGGAGCCTTTCAAC------------AAGTTCATGCAGTTCAAGGATAGCAC
TACCAAAGCTCTGGAAGGCATCTGCGGCCACTACGTTAGTGTTGTTGTTGACCCTATGGCATCTGA
CGAAGAGATCAAGAAAGCCCTGAGGAAGAAATATGGTCGCCACATCGGAGAGCTCAAAGCTGAGT
TCAATCGTGTGCGCAAGAAAGGGAAGCTGCCTTCCAGTGCTCGCTCCATCTTGAAGGACTGGTTC
AACCGCCATTCTTACTGGCCTTACCCTTCAGAGATGGAGAAACAGTACCTGCAAAAACTCTGCGGT
CTAAACCTAAAGCAAATTAACAACTGGTTCATCAATGAGCGCAAGCGGCACTGGAGCTGCGAGGG
T
>PpMKN6
ATTAGAAACCGAGCACTGATAGTAAATCATCCCTTGTATCCAGAAATGCTCATGAATCATGCGGCT
TGCCTTCGAGTTGGGACCCCTGTTGATCAACTTCCAAGCATTGAAGCCCAATTGGCTCAAGCACCT
AATATCATTGAGGACGAGAAAGTGGAGCTTGACCGCTTCATGACCGAATATACAGCCCTACTGGG
TGATTTCAAGGACGTATTGCAACATCATGTCTACACAGACGTAGCAGAAGCAATGATAGGATGCTG
GGAGCTGGAACAAGCGCTTCATGCTCTCACTGGAGTATCTCCGACGGAGCGTAGTTTAATGGAGC
GGGTGCGCCAGGAGCTCAAGCATGAACTCAAACAGGGCTACAGATCTAAAATAGAGGACGTTCGT
GAGGAAATTTTGCGCAAACGGAGGGCTGGGAAGTTGCCTGAGGGCACTACAACTGTCCTCAAGG
CGTGGTGGCAAGCACATTCGAAATGGCCCTACCCAACGGAGGATGAGAAAGAACAATTAATACAG
GAGACAGGATTGGAGCTCAAGCAAGTCAACAACTGGTTTATAAACCAGCGAAAGCGAAACTGGCA
TAGCAACCCC
>PpuHPXA-2020612_liverworts
AACCGGGCCAGGAGAGCGATTCAGACGCATCCACTCTACCCATATCTCGTTTCTGCCCACATGGA
CTGTTATAAAGTAGGCGCAGAAGCAGCAGATCTCCAAAAACTGGAGGAGGTCAATTGGAAATATCA
GCACCTTCAGCTAAATTCTCATTCCGAGTTGGACAGCGTGATGTCGTGGTACCTCGGCATTCTTCT
CGACTTGAAAGGAAAGGTGGAGGAGCCATTTTTG------------GCGGAGATGGAATGGTGCAAAGAAA
TCTATGAAGAGGTGAAGCTTCTGGGTCCAGTAATTGCTGGTGTAACAGTCGATCGCACAGCAGAC
GAGTATGCTGTTAAGGAGTGGCTAAAATGGAGGCATGACCCTTGCATACGCAACTTGAGAGAGCA
ATGCAGGAAGAAACGGAGGCAAGGGAAACATTCCGAAACAGCTCGAGAACGTCTAGTGGAGTGG
TTCAATGGTCATGGGGAATGGCCTTACCCCTCGGAGGACGAGAAGCTGCAGCTGGCGGAGCAGA
CCAAGCTGGAGGGTCGGCAAATCAACAACTGGTTCATAAACCAGCGGAAAAGGCATTGGGAGAAT
TCTGCA
>SfaIGUH-2020050_mosses
GCCAGAGACAAAGCATTGATTGTGGCTCATCCACTATACCCAGAGTTGCTGAATGCACACGCGTC
TTGTCTTCGAGTGGGAACCCCAGTGGATCAGCTCCCACATATTGAGGCACAATTAGCTCAAGCTG

GATATGTCACAAACGATGAAAAAGTTGAACTTGATCAGTTCATGACTCAGTATATCATGCTTCTTTG
CTCGTTTAAAGAACATCTACAACAACACGTCTACTCTGATGTTACTGAGGCAATGATGTCTTGCTG
GGAGCTTGAGCAGGCCTTACATTCACTCACTGGGGTCTCTGCCTCAGAACGTACTCTCATGGAAC
GAGTTCGCCAAGAACTCAAATACGAGCTGAAGCAGGGTTACAGAGCAAGAATCGTTGATGTACGA
GAGGAGATTCTGCGCAAACGACGAGCTGGGAAGCTGCCTGAGGGTACAACAACAGTTTTGAAAGC
TTGGTGGCAAGCACACGCTAAGTGGCCTTATCCAACGGAGGATGAGAAGGAGAAGCTGATACACG
AGACTGGTCTGGAATTGAAGCAAGTGAATAACTGGTTCATTAATCAGCGTAAGCGGAACTGGCACA
GCAATCCG
>SfaIGUH-2165279_mosses
GCCAGGAAGTGCTGTATGATTGTAAATCATCCCCTCTATGAAGAAATGCTCGCAAATCACGCGGAT
TGCCTCCGTGTTGGAACTCCAACAGATTTAATTTCAAATATCGAAGCCCAGATGTCTCAGGCCCCG
ATAGTCATTGAGGACGAGCAATTGGATCTAGACCGCTGCATGACCGATTACACTATGGTCCTGGCT
GCATTCAAGGATCTCTTGCAGCATCACGTGTTCACAGACGTCACGGAGGCAATGATGTCATGCTG
GGAGTTGGAACAGGCTCTTTATGCTCTCACTGGAGCCACTCCAACAGAGCGAACTCTCATGGAGC
GCGTGCGCCAGGAACTCAAGCATGAACTCAAGCAGGGCTATAAATCCAAAATTGAGGATGTTCGA
GAGGAGATTTTGCGCAAACGGCGCGCTGGAAAACTTCCTGAGGGCACCACAACTGTTCTCAAAGC
TTGGTGGCACGCACATTCGAAGTGGCCTTACCCAACGGAGGATGAGAAAGAACTCTTAATACAGG
AGACAGGATTGGAGCTGAAGCAAGTCAACAATTGGTTCATTAATCAAAGGAAGCGAAAC-------------
-
>SkKNOX1
GAGATGCTTCGGGCTGCCATTGTGTCTCATCCCCACTACCCCGAACTCGTGGTTGCACATATGAA
CTGTCACAAGGTGGCTGCGTCACCAGAAGTCGTTTCCCAGATTGACGAGATTATCCAGAACTTCAA
GGACTTCCAGCCGGGGGCGAACCCGGAACTCGACCAGTTCATGGTTGCATACTACTCGATGCTCC
TGAAATGCGAGAAGGAAGTCAGGAAGACGTTCAAG------------GAAGCCGTGGCATTTTGCAAGAAA
CTGGACCAGCAGTTTCAGGTCATCACCAACGGAAGTGCGGAAATCGAAGTTGATCCCATGGCCAA
AGATAAAGAGCTCAAGGAGCAGCTCATGAGGAAGTACAGCGGATATATCAGCAGCCTCAAGCACG
AGTTCCTGAAGAAGAAAAAGAAAGGTAAGCTTCCCAAGGACTCGAGGCAGATACTGCTCAACTGG
TGGTCCGTTCACTACAAATGGCCGTATCCCTCGGAATCGGAGAAAGCAAGCTTGGCCGAATCGAC
TGGACTCGACCAGAAGCAGATCAACAACTGGTTTATCAACCAGAGGAAGCGCCACTGGAAGCCTT
CGGAC
>SkKNOX2
AAAGAGATGAAGGCTGCAATCAGCGGCCATCCTCAGTACTTGGAGCTCATCAAAGCACATATGAG
TATCAAGAAGGTCGGAGCGTCGTCCCAGAAGGTGGCTGAAATAAACGAGGTCATTCGTATGCACC
AGGATTCACAACCAGGAGCAAATCCTGAACTGGACCAGTTTATGGTTGCGTATTGCGATGTTCTGA
ACATGTACGAGAACCAACTGAACAAGGCATTCACA------------GGCGCAATTGAGTATTGCAAGCAGC
AGGAGCAGGAGCTTAAGCTGGTTTCCGTTTCTGATGAAGGCGGCGACATTGACCCTCTCATTGGT
GACAAGGAGATTAAGCGGGCGCTTATGAAGAAATACGGGGGTTATCTTGGTGGCTTAACGCAAGA
GTACCTCAAAAAGAAGAAGAGCAAGCTACCTTCTGCTGCGACAAAGACACTTCGGGACTGGT
GGTTTCAGCACCTGGAACATCCTTATCCCTCGGAAGCTCAGAAGGCAACTCTTGCGGCTACTACA
AAGCTCGATCCTAAGCAAATCAACAACTGGTTCATTAACCAAAGGAAGAGGCACTGGGATCCTTCT
GCA
>SkKNOX3
GCTAAGCTCAAGGCCGACATAGTGACGCACCCTCTTTACGAGCAGTTGCTAGAGGCGCACGTTTC
ATGTCTCCGGATTGCTACTCCAGTGGACCAGCTGGGTAAGATCGATGGACAAATCGCACAGTGTC
ATCAACTCATTGCAAACAGCAAGGACGAACTGGACCAATTTATGGCTCACTACGTTATGCTCCTGA
GGTCTTTCAAGGACCAGCTGCAGCACCATGTCCGAGTTCATGCCAAAGAGGCCGTCATGGCTTGC
TGGGAGCTGGAGCAGTCCCTGCTCGGACTAACCGGTGTATCACCCACGGAACGGACTCTCATGG
AGCGAGTCCGGCAAGAGCTCAAGCACGAGCTGAAACACGGCTACAGAGCGAGGATTGTAGACGT
TCGGGAKGAGATACTACGCAAACGTCGGGCTGGAAAGCTTCCGGGAGACACCACATCTGTGCTTA
AAGCATGGTGGCATGCACATTCCAAATGGCCGTATCCGACGGAGGATGAAAAGGCACGCCTTGTT
CAAGAGACGGGGTTGGAGCTGAAGCAGATCAACAACTGGTTCATCAACCAACGGAAACGTAACTG
GCATCACCATCCA
>SpKNOX1
AATGCTATCAAAGCTGAAGTTGTTTCTCATCCTCTGTTTAATCAGCTACTCGAGGCTCATGTGGCGT
GTCTTAAGGTTGCAACGCCTCTCGATCAAATCCCCTTGCTAGACCTCCAATTAGAAAAGAAAAATC
TCGTGATTGAAGAAGAAACAACCGAGCTTAATTTTTTTATGACGCATTTCGTGTGTTTACTTCAATC
TTTCAAAAGCCAACTAGAGCAACATGTATTGATTCAAGCCAGAGAAGCAGTAATGTCATGTTGGGA

ATTAGAACAATCCCTCATTTCGTTAACAGGTATGGAGCCTAACGAACGCAGCATTATGGAACGTAT
GAGGACGGAACTTAAAAAACAGTTAGATCAAGGCTATCGTACCAAAATAGCAGACGTGCGTGAGG
AAATTATGAGAAAAAGAAGGGCAGGAAAGTTGCCTAGCGATACGACCCAAATTTTAAAGAGTTGGT
GGAATGCCCACTCAAAATGGCCGTATCCTACAGAAGACGAAAAGACTACTCTGGTTCGGGAAACC
GGTTTGGAACTGAAGCAGATAAACAACTGGTTTATAAACCAGAGGAAAAGAAACTGGCACAACAAT
AGC
>SpKNOX2
TGTGGGTATAAAGAGCAAATTATTTCACACCCATTGTTTCCTGACCTCGTTTCAAAATTCATCGAAT
TACGCAAAGTAGGGGCACCTGAAGAAAAAATGAAAGAACTAGAAAATGAACGTGAAAGTCTTTTAA
ATTTAATGAATGATCATGATGAAAATTTGGATTCTTTTATGAATGGTTTTTTGGTTCAATTGTCTGAA
TCGAAAGTTGAAATTCAAAAAATCTTTGAC------------GAGGCTACTAAATTTTGCTCAGATTTTGAGA
AGAATATTTGCCAAAATTTGATGTCAAATAAATTTCAAGACGTAGATCCTACCGCTGAAGACAGGGA
ATTGAAGGAGCAGCTGAAAAGAAAGTATAGAGCTTATTTGACGGGTTTAAAACAAGAATTCTTAAA
GAAGAGAAAGAAGGGGAAGCTTCCAAAAGAAGCCACCATGATTTTGCATAACTGGTGGTTTTCTCA
CTTAAATTGGCCTTATCCGTCGGACGCGGAGAAAACACAGCTAGCGGAAATGACCCGTCTTGACC
CGAAGCAAATTAATAACTGGTTTATAAATCAACGGAAGCGACATTGGAACAACGCACCT
>SteHERT-2043328_liverworts
TGCCGTTTCAAGGCTAGAGTGTCGAGTCATCAGCTGTTTCCTCAACTGTTGAAAATACACATTGAT
ATCATCCGCTTCATGACCCCATTCAACCTGCAGCGTGAGATTGACGTACAGCTCAAGAAGTCTCAG
GTCGTCCACGACCCCGACGACAAAGCGTTTGATGCTTTTCTGATGCTATACCTGAAGGAGCTCAC
CGACTATCGAGACCGTCTTGTGAAGGACCCAGAGGCGTCAACTACTCGTGCTTTTGTTCACATGT
GGCATTTCGAGCGTGCAGTTTTTGAAATGACTGGAATCCCTCCCTCAGAGCGCTCGCTGATGGAG
CGCGTGAAAAAAGAGCTCAAAGCGAGTCTCGAGGAAGGATATCGAGCGAAGATTTCAGAAGTAAG
GGAGGAGATCATGCGAAAGCGCAGAGCAGGAAAACTTCCCGAAGGAACCACGACAACACTGAAA
GCGTGGTGGGATGCACATGCGAAGTGGCCTTATCCTACGGAAGATGAGAAAGAAGTGCTGATCCA
AAAGACAGGACTAGAGCTGAAGCAAGTCAACAACTGGTTCATCAACCAGCGCAAGAGGAACTGGC
ATAATAACCCT
