# Supplementary material for: Nonreciprocal complementation of KNOX gene function in land plants
Source: New Phytol. 2016 Nov 25;216(2):591–604. doi: 10.1111/nph.14318 (PMC5637896; doi:10.1111/nph.14318)
Supplement: Supplementary file 8 — Notes S6 Edited BELL alignment. [file NPH-216-591-s008.pdf]

Supporting Information Notes S6 Edited BELL alignment.

>AXG93_855s1120
-------IREIEALFREVLSFSRSRGVTLHDRKKMFRAYIDEIDDWYYEYRSTLENINDETDDSFGHFIGNIYT
EYPLMELSRRLRRYRDTFLEHLVCVKKVLGDST-----------RQQRMYSGRLSTWRPQRGLPDRAVSVLK
AWLFTNFLHPYPKDVDKESLASATGLTRSQVSNWFINARVRVWKPMIEAMYLMDFP
>AWOI-2011929
HFSASRFLRSAQSILNEVCRVTQDNREDLELKKGKLGIMLDEVDARYRRYCDHLQLLITGFNSQAGPNT
ATPYTILALQAMSRHFRCLRDAIGAQLRVVKRALGEDDTSRLRYVDQQIRQQRALLGMLHAWRPQRGL
PERAVSVLRAWLFEHFLHPYPKDVDKMSLAKQTGLTRSQVSNWFINARVRLWKPMVEEMYLEEQR
>Aagrestis34670
------------------------MMEELDNQYHRASSAYIEVDGTYRGYSEQMRVVITTMDVALGKGAAQSYTNLAFE
AMSRHFRGLRDTIEGQLRVCNRLLGEEDIPRLRFVDQQLRQQRALLGMLHAWRPQRGLPERAVTVLR
AWLFEHFLHPYPKDGDKMSLARQTGLTRSQVSNWFINARVRLWKPMVEEMYLEESK
>BEL1
QIGSSKYLSPAQELLSEFCSLGVLEFMELQKRKAKLLSMLEELKRRYGHYREQMRVAAAAFEAAVGLG
GAEIYTALASRAMSRHFRCLKDGLVGQIQATSQALGERETPRLRLLDQALRQQKSYMTLVHPWRPQR
GLPERAVTTLRAWLFEHFLHPYPSDVDKHILARQTGLSRSQVSNWFINARVRLWKPMIEEMYCEETR
>BEL10/BLH10
SVLRSRYLKPAQNLLDEVVSVKKIEREELQNKKNKLLTMVDEVDKRYNQYYHQMEALASSFEIVAGLGS
AKPYTSVALNRISRHFRALRDAIKEQIQIVREKLGEKGIPRLRYLDQRLRQQRALLGMVPAWRPQRGLP
ENSVSVLRAWLFEHFLHPYPKESEKIMLAKQTGLSKNQVANWFINARVRLWKPMIEEMYKEEFG
>BLH1/EDA29
NLVSSKYLKAAQELLDEVVNADSAERQEIQMKKAKLSNMLHEVEQRYRQYHQQMQMVISSFEQAAGI
GSAKSYTSLALKTISRQFRCLKEAIAGQIKAANKSLGEEDGSRLKFVDHHLRQQRALLGMINAWRPQRG
LPERAVSVLRAWLFEHFLHPYPKDSDKHMLAKQTGLTRSQVSNWFINARVRLWKPMVEEMYMEEMK
>BLH2/SAW1
ILRNSRYTTAAQELLEEFCSVGRSDRIEHQRRKVKLLTMLEEVDRRYNHYCEQMQMVVNSFDIVMGHG
AALPYTALAQKAMSRHFRCLKDAVAAQLKQSCELLGDKDTPRLRLLEQSLRQNRAFMGMMEAWRPQ
RGLPERSVNILRAWLFEHFLHPYPSDADKHLLARQTGLSRNQVSNWFINARVRLWKPMVEEMYQQES
K
>BLH3
SVLRSRYLKPTQQLLDEVVSVRKSERQELQSKKSKLLTMVDEVDKRYNQYHHQMEALASSFEMVTGL
GAAKPYTSVALNRISRHFRCLRDAIKEQIQVIRGKLGEREIPRLRYLDQRLRQQRALLGMVPAWRPQRG
LPENSVSILRAWLFEHFLHPYPKESEKIMLSKQTGLSKNQVANWFINARVRLWKPMIEEMYKEEFG
>BLH4/SAW2
TLRNSKYTKPAQELLEEFCSVGRADRIEHQRRKVKLLSMLEEVDRRYNHYCEQMQMVVNSFDQVMGY
GAAVPYTTLAQKAMSRHFRCLKDAVAVQLKRSCELLGDKETPRLRLLEQSLRQQRAFMGMMEAWRP
QRGLPERSVNILRAWLFEHFLNPYPSDADKHLLARQTGLSRNQVSNWFINARVRLWKPMVEEMYQQE
AK
>BLH5
RALAPIYLKAAQELLNEIVNVGNPGVAALQMKKAKLISM---VEQRYKQYHDQMQTIISSFEQAAGLGSAN
SYTHMALQTISKQFRAVKDMISLQIKQINKLLGQKE------FDEQLKK----LGKMNAWRPQRGLPEKAVSVL
RSWLFEHFLHPYPRDLDKVMLAKQTGLTKSQVSNWFINARVRMWKPLVEELYSEEMD
>BLH6
TIPNSKYLKAAQQLLDEAVNVKKSERQEMQSKLTKLLSMLDEVDRRYKQYYQQMQIVVSSFDVIAGYG
AAKPYTALALQTISRHFRSLRDAISGQILVLRKCLGEQQISRLKYVDQHLRQQR---GFMQAWRPQRGLP
ENSVLILRAWLFEHFLHPYPKDSDKIMLARQTGLSRGQVSNWFINARVRLWKPMVEEIYKEEFT
>BLH7
TIHNSKYLKAAQELLDETVNVKKAERQELQSKLSKLLSILDEVDRNYKQYYHQMQIVVSSFDVIAGCGAA
KPYTALALQTISRHFRCLRDAISGQILVIRKSLGGEQISRLRNVDQQVRQQRALLGVMHTWRPQRGLPD
SSVLVLRAWLFEHFLHPYPKDSDKIMLARQTGLSRGQVSNWFINARVRLWKPMVEEMYKEEFT
>BLH8/PNF
ILKSSRFLEPAQKMLEEFCISYA-------LKKAKLLFLQEEVCKWYKLYNHQLQTVMSSFNTVAGLNTATPY
ISLALKRTSRSFKALRTAIAEHVKQIS------------------KRQRSLVGFEHIWRPQRGLPERAVAVLRAWLFD
HFLHPYPTDSDKQMLATQTGLSRNQVSNWFINARVRLWKPMVEEIHTLETK
>BLH9/BLR/PNY/RPL/VAN
ILKGSRFLKPAQMLLDEFCNVGR-------KKKSKLISMLDEVYKRYKQYYEQLQAVMGSFECVAGLGHAA

PYANLALKALSKHFKCLKNAITDQLQ--------------------------HGFPPVWRPHRGLPERAVTVLRAWLFDHF
LHPYPTDTDKLMLAKQTGLSRNQVSNWFINARVRVWKPMVEEIHMLETR
>CAPN-2001274
-----------------------SERQDLQMKKTKLMAMLEEVDRRYRQYYFQMQMVVSSFESAAGVGSAKTYTSLAL
KTISRHFRCLRNAIGGQVQAACKSLGEAD------------------GMMHVWRPQRGLPERSVSILRAWLFEHFL
HPYPKDVDKVLLAKQTGLSRSQVSNWFINARVRLWKPMVEEMYTEEMK
>CAPN-2002967
-----------------------SERQELQMKKAKLMAMLDEVDRRYRQXYFQMQMVVSSLDSAAGIGSAKTYTSLALK
TISRHFRCLRDAICSQIRAACDSLGETE-----------------LGILHVWRPQRGLPERSVSILRAWLFEHFLHPY
PKDGDKVMLARQTGLSRSQVSNWFINARVRLWKPMVEEMYTEEMK
>CAPN-2007100
LMRNSKYLKPVQHLLDEFCNVRVEERSQLQMKKTRLISMVEEVDRRYRQYRDQMQTVITSFESATGL
GGAAPYTTLARQTMSRHFKCLRDAISNEIRAICGTLGEEPTPRLRILDQQLRHQRSLMGMLQPWRPQR
GLPERAVSVLRAWLFEHFLHPYPKDSEKLMLARQTGLTRNQVSNWFINARVRLWKPMVEEMYVEEQK
>CAPN-2007101
-----------------------EERSQLQMKKTRLISMVEEVDRRYRQYRDQMQTVITSFESATGLGGAAPYTTLARQ
TMSRHFKCLRDAISNEIRAICGTLGEEPTPRLRILDQQLRHQRSLMGMLQPWRPQRGLPERAVSVLRA
WLFEHFLHPYPKDSEKLMLARQTGLTRNQVSNWFINARVRLWKPMVEEMYVEEQK
>CrBELL1.Transcript_12985
YLTNSKYLRVAQLLLEEVVNIGCQENQELQLKKAKLVAMLDEVDRRYKLYFSQMEAVVNTFESAAGLG
AAKTYTALALQTISKHFRGLRDAIGSQIRAASRALGQEDFGRLRYVDQQLCQPRTFLGMMHAWRPQR
GLPERSVSVLRAWLFDHFLHPYPKDADKHMLAEQTGLTRNQVSNWFINARVRLWKPMVEEMYLEELK
>CrBELL2.Transcript_22605
DLWTSKYLKPAQQLLDEVVSVVKTDRKEWQIKKAKLLAMVDEVDQRYRLYIRQMQNLEAPFQTIVGLG
AAKAYTALASQTISKHFRCLKDAMKVQLQAVCKTLGEEVRSGLLDVDKELYQHHALLGIIHPWRPQRGL
PERSVSVLRAWLFEHFLHPYPKDSDKVRLARQTGLTRNQVSNWFINARVRLWKPMVEEMYTEVKN
>CrBELL3.Transcript_69280
ILRNSKYLKPAQLLLNEFCNVCKDERCHLQLRKARLIAMVEELDRRYQQYRDQMQLIVTSFESATGIGG
AAPYTTLAKRAMSRQFRSLRDAIGEHIKAVCRTLGEEVTPRLRLIDQRLRHQRSLISMLQAWRPQRGLP
ERSVSVLRAWLFEHFLHPYPKDSEKAMLARLTGLSRNQVSNWFINARVRLWKPMVEEMYLEESK
>CrBELL4.Transcript_82348
GSYGSKYLRPAQELLNEVVNVGQADRQELQAKRAKLVGMRDEVDRRYKQYYHQMQAVATSFESVAG
LGAAAAYTALALQTISKHFRCLRDAITGQIQATCKALGEEETSRLRFIDQQLRQQRALLGMMQAWRPQ
RGLPEHSVSILRAWLFEHFLHPYPKDADKIMLAKQTGLNRNQVSNWFINARVRLWKPMVEEMYLEETK
>LOC_Os01g62920.1
VLGRSRFLGPAEKLFEEICDVGGISGAEQQWKKTKLISMMEEVCKRYRQYYQQVQAVMASFETVAGF
SNAAPFAALALRAMAKHFKCLKSMILNQLRNTSDGLNKEI-----------------FGQPNIWRPQRGLPERAVSV
LRAWLFEHFLHPYPTDGDKQMLAKQTGLTRNQVSNWFINARVRLWKPMVEEIHNLEMR
>LOC_Os03g03260.1
HLRSSRFLLPTQQLLQEFCSLPVMEALELQRLKDKLYIMLEEVDRRYRRYCEQMRAVAGGFEAVAGER
AAGAYTAVAARTISRHFRSLRDGIVAQLQAARKALGEKDTPRLRVIDQCIRHHKSLVAAMHPWRPQRG
LPDRAVTILRAWLFEHFLHPYPSDVDKHILARQTGLSRSQVSNWFINARVRLWKPMVEEMYVEEMK
>LOC_Os03g06930.1
YVQNSRFLRAARELLDEVVNVRDSERQDLQNKVTALMAMLDQVDRRYRHYHHQMQIVMSSFDAVAG
GGAARPYTALALQTISRHFRSLRDAIGAQAQAARRGLGEQDLSRLRYIDQQLRQQRAMFGMMHAWRP
QRGLPESAVSVLRAWLFEHFLHPYPKDSEKLMLARQTGLSRGQVSNWFINARVRLWKPMIEEMYKEE
FG
>LOC_Os03g47740.1
AIRNSKYLKAAQELLDEVVSVWKAEKQELQNKMAKLMAMLDEVDRKYKHYYHQMQIVVSSFDMVAGS
GAAKPYTAVALQTISKHFRCLKDAINDQINVIRKKLGEEELTRLRYIDQQLRQQRAFYGLLNAWRPQRG
LPENSVSILRAWLFEHFLHPYPKDSEKLMLARQTGLTRSQISNWFINARVRLWKPMIEDMYKEEIG
>LOC_Os03g52239.1
VLRNSKYTRAAQELLEEFCSVGRADRFEHQRKKAKLISMLDEVDRRYNHYCDQMQMVVNFFDSVMGF
GAATPYTALAQKAMSRHFRCLKDAIAAQLRGTCEALGEKDTPRLRAIDQSLRQQRAFMGIMEAWRPQ
RGLPERSVNILRSWLFEHFLHPYPSDADKHLLARQTGLSRNQVSNWFINARVRLWKPMIEEMYQQEC
K
>LOC_Os05g38120.1
VLGRSRFLGPAQKLLEEICDVGGVSGAEQQWRKTRLISLMEDVCKRYRQYYQQLQAVVSSFETVAGL

SNAAPFASMALRTMSKHFKYLKGIILNQLRNTGDGLGKED-----------------FSQPNIWRPQRGLPERAVSV
LRAWLFEHFLHPYPTDSDKQMLAKQTGLTRNQVSNWFINARVRLWKPMVEEIHNLEMR
>LOC_Os06g01934.1
QLRGSKYLGPVKALLAEFCSLDVMDLLDLERRKARILSMVEEVDRRYRRYREQMRAVEVSFEAVAGG
GAAQVYTKLAMRAMSRHFRCLRDALVGQVRALRNAMGESQTPRLRVLDQCLRQQRAFSGAVFPWRP
QRGLPERAVAVLRAWLFEHFLHPYPNDVDKHILARQTGLSRSQVSNWFINARVRLWKPMIEDMYKEET
K
>LOC_Os10g39030.1
HLRGSRFLLPTQQLLQEFCSLPVMDAAELQRLKGKLYTMLEEVDRRYRRYCEQMRALAASFEAVAGE
RAAASYTRLASRTISRHFRSLRDGVVAQLQAVRKQLGEKDTPRLRVLDQCLRQHKAYAGMLHPWRPQ
RGLPERAVSILRAWLFEHFLHPYPSDVDKHILARQTGLSRSQVANWFINARVRLWKPMVEEMYAEEMK
>LOC_Os11g06020.1
MVMSSKYLKAAQELLDEVVSVSKAERQELQMKKGKLINMLDEVEQRYRQYHQQMQVVVASFEAVAG
GGSARTYTALALRTISRQFRCLRDAIAGQVRAASRALGEAVGSRLRYIDHQLRQQRALLGMMSAWRP
QRGLPERSVSILRAWLFEHFLHPYPKDSDKIMLAKQTGLTRSQVSNWFINARVRLWKPMVEEMYLEET
K
>LOC_Os12g43950.1
AIRNSRYLKAAQELLDEVVSVWKAEKQELQNKMAKLMAMLDEVDRKYKHYYHQMQTVVSSFDVVAGP
GSAKPYTAVALQTISRHFRCLKDAINDQINVIRKKLGEEELTRLRYIDQQLRQQRAFYGMINAWRPQRG
LPENSVTILRAWLFEHFLHPYPKDSEKLMLARQTGLTRSQISNWFINARVRLWKPMIEDMYKEEIG
>MA_10435446g0010
VLKSSKYLKPAQQLLDEFCNVGRGDRFEIQRRRAKLLSMLEEVHRRYGQYCEQMKMVVTSFESIAGLG
AATPYTALALKAMSRHFKCLRDAINNQLRVTSKALGEDSTPRLRFIDQNIRQQRAVLGMLHAWRPQRG
LPERSVSVLRAWLFEHFLHPYPTDADKHMLARQTGLSRSQVSNWFINARVRLWKPMVEEMYLQESK
>MA_111188g0010
VLTGSKYLKAAQQLLDEVVNVGKAERQELQMKKAKLVAMLDEVDQRYRQYYHQMQIVVSSFEATAGF
GAAKTYTSLALQTISRHFRCLRDAISGQIRMTSKSLGEEDTSRLRFVDQQLRQQRALLGMIHAWRPQR
GLPERSVSVLRAWLFEHFLHPYPKDADKHMLARQTGLTRNQVSNWFINARVRLWKPMVEEMYNEETK
>MA_18002g0010
SLTGSKYFKAAQQLLDEVVNVGKADRQELQMKKAKLVAMLDEVDRRYRQYYHQMHIVVSSFEAAAGF
GAAKTYTSLALKTISRHFRSLRDAIAGQIRLTSKNLGEEDTSRLHFVDQQLRQQRALLGMIHAWRPQRG
LPERAVSILRAWLFEHFLHPYPKDADKHLLARQTGLTRSQVSNWFINARVRLWKPMVEEMYMEELR
>MA_28447g0010
LLENPKYMKVTQELFDELSNVGLGDLLALQKRKKELLWLLDEVQRQYRQYCDHREMVISSFESIAGVG
AATPYLAPASKAMSRHFRCLKDAIKDQVKATCELLGEKETPRLRFLEQSLRQQRAFVGMIEAWRPQKG
LPQHSISVLRAWLFEHFLNPYPSDADKHMLARKTGLTRNQVSNWFINARVRLWKPMIEDMYQEELK
>MA_6763g0010
SLKNSRYLKAAQEILYEFCNVGREDRFELQRRKAKLVSMLDEVDRRYRNYCDQMHLVVSSFESVSTLG
AAAPYTALALKAMSRHFRCLKDAITGQLQVTIKALGEKGTPRLGFLERSIRQQRAFLGLMHPWRPQRG
LPERSVSVLRAWLFEHFLHPYPTDADKHILAKQTGLTRSQVSNWFINARVRLWKPMVEEMYMEEIK
>MA_76835g0010
---MQIVVSSFETVTGFGTAKTYTSLALGTISRHFRCLRDAIVGQIC
VSSKSLGEEDTSRLRFVDQQLRQQRALLGMIPAWRPQRGLPERAVSVLRAWLFEHFLHPYPKDADKH
MLARQAGLTRSQVSNWFINARVRLWKPMVEEIYMEEMK
>PQTO-2005856
FLSRSKYLKAAQQLLDEVCSVRMEERKDCETKKGKLLAMLQEVDRRYRIYSDQMHAVVASFDIVVGVG
AATPYTALALQAMSRYFRCLRDAISSQIQLICKALGEEEISRLRYVDQQLRQQRSFLGMLHAWRPQRGL
PERSVSVLRAWLFEHFLHPYPKDADKIMLARQTGLTRSQVSNWFINARVRLWKPMVEEMYQEEAR
>PQTO-2013388
TLSSSKYLKVAHRLLDEVCSVLGEERQTSQIRKTKLLSMLQEVDRQYRQYYDNMQALIFSFDSAFGIGA
ARPYTRMAVKAMSRHFRSLRDTIRNQIQITCKSLGEEYTTRLRFVDQQLRQQRALLGMLPAWRPQRGL
PEQSVSVLRAWLFEHFLHPYPKDADKIMLAKQTGLSRSQVSNWFINARVRLWKPMVEEMYTDENR
>PQTO-2013389
TLSSSKYLKVAHRLLDEVCSVLGEERQTSQIRKTKLLSMLQEVDRQYRQYYDHMQAVIFSFDSAVGVG
AARPYTRLALKAMSRHFRCLRDTISNQIQITCKALGEEDTSRLRFVDQQIRQQRALLGMLPAWRPQRG
LPEHSVSILRAWLFEHFLHPYPKDADKMMLARQTGLSRSQVSNWFINARVRLWKPMVEEMYLEENR
>PQTO-2013390
MLSSSRYLRVVQHLLDEVCSV-GEERQQYQIRKTQLLSMLQEVDRQYRQYYDHMQAVIFSFDSAVGV

GAARPYTRLALKAMSRHFRCLRDTISNQIQITCKALGEEDTSRLRFVDQQIRQQRALLGMLPAWRPQR
GLPEHSVSILRAWLFEHFLHPYPKDADKMMLARQTGLSRSQVSNWFINARVRLWKPMVEEMYLEENR
>PpBELL1
HISASKFLRSAQAILNEVCRVTPESRDDLELKKQKLSLMLDEVEARYRRYCDHLQLVITGFNSQAGPNT
ATPYTILALQAMSRHFRCLKDAIGSQLRIVKRTLGEDDTSRLRYVDQQIRQQRALLGMLHAWRPQRGL
PERAVSVLRAWLFEHFLHPYPKDVDKLSLAKQTGLTRSQVSNWFINARVRLWKPMVEEMYVEEQK
>PpBELL2
HISASKFERSAQAILNEVCSVTPENRVDLELKKQKLNLMLDEVETRYRRYCEHLQLVITGFNSQAGPST
ATPYTILALQAMSRHFRCLKDAIGSQLKIVKRSFGEDETSRIRYVDQQIRQQRTLLGMLHAWRPQRGLP
ERAVSVLRAWLFEHFLHPYPKDVDKMSLAKQTGLTRSQVSNWFINARVRLWKPMVEEMYVEEQK
>RXRQ-2024990
------YARAGRELLHEICSPCTEQRSQIEAKKARLTGMMEEVDGTYRGYSEQMRVVITTMDVALGKGAA
QSYTNLAFEAMSRHFRGLRDTIEGQLRVCNRLLGEEDIPRLRFVDQQLRQQRALLGMLHAWRPQRGL
PERAVTVLRAWLFEHFLHPYPKDGDKMSLARQTGLTRSQVSNWFINARVRLWKPMVEEMYLEESK
>RXRQ-2142126
------YAKSGQDLLLEICSPAGDQKAQVEARKTRLISMIGDVDGQYRTYIDHMKVVMVTIDTVMGKSSSLP
YTKLALNAMSRHFRALRDAIEGQLRVCNRLLGEPNLPRLKYLDAQLRQQKAMLGMLHAWRPQRGLPE
KAVILLRAWLFEHFLHPYPKDSDKITLAKKTGLTRSQVSNWFINARVRLWKPMVEEMYMEESR
>SkBEL1
IISSSRYLKACQQLLEEVCSVGREERQEFELKKSKLIAMLQEVDRRYRQYNDQMQVVISSFDAVAGTGS
ATPYTALALQAMSRYFRCLKDAITGQIQLVCKALGEEDTSRLRFIDQQIRQQRAFLGMLHAWRPQRGLP
ERSVSILRAWLFEHFLHPYPKDADKMMLARQTGLTRSQVSNWFINARVRLWKPMVEEMYQEETR
>SkBEL2
FVSGSRYLRAAQQLLDEVCSVGREEKQDHEGKKTKLLAMLQEVDRRYRQYYDQMQVVITSFDTVAGV
GAATPYTALALQAMSRYFRCLRDAITGQIQITCKALGEEDTSRLRFIDQQIRQQRAYYGMLHAWRPQR
GLPERSVSILRAWLFEHFLHPYPKDADKMMLARQTGLTRKQVSNWFINARVRLWKPMVEEMYQEETK
>ZTHV-2017075
HIATSRFLKTAQDVLNEVCRVSQESREELELKKGRLILMLDEVEARYRRYCDHLQVVITRFNSRAGPGA
ATPYTILALHAMSRHFRCLRDAIRHQLKVVKRALGEEETTRLRYIDQQMRQQRTLMQMQNAWRPQRG
LPERAVSVLRGWLFEHFLQPYPKDVDKLSLAKQTGLTRSQVSNWFINARVRLWKPMVEEMYLEEQK
>evm_27.model.AmTr_v1.0_scaffold00001.384
EIKNSRYLVPAQKLLYEFCNLGSSDLFELQKRK---------VDKRYKQYCERMRSVASSFEAVAGAGAAHV
YLGMASRAMSRHFRCLRDGIVAQLRATRVALGEKETPSLKLVDQRLRRQRALMGMMHPWRPQRGLP
ERSVSLLRAWLFEHFLHPYPSDVDKHILARQTGLSRSQVSNWFINARVRLWKPMVEEMYLEEAK
>evm_27.model.AmTr_v1.0_scaffold00002.320
TISNSKYLKAAQQLLDEVVNVGNTERQDLQTKLTKLLGMLDEVDRRYKQYYHQMQIVVSSFDAIAGAG
AAKTYTALALQTISKHFRCLRDAINGQIRATQKSLGEQDLSRLRFVDQQLRQQRALLGMMHAWRPQRG
LPESSVSILRAWLFEHFLHPYPKDSDKLMLARQTGLSRSQVSNWFINARVRLWKPMVEEMYKEEIG
>evm_27.model.AmTr_v1.0_scaffold00009.52
ILKSSKFLRPAQLILSEFCSVTKGDGIEFHKKKTRLMALFDEVYRRYRIYQHQMQSVISSFESIAGLSAAT
PYTSLALKAMSKHFRCLKNVISDQLKQMSSLLGEEEIPRLRFLDQRIRHQQQQMGLEHVWRPQRGLPE
RSVAVLRAWLFEHFLHPYPTDNDKHVLASQTGLSRNQVSNWFINARVRLWKPMVEEIHMLESK
>evm_27.model.AmTr_v1.0_scaffold00030.108
VLMGSKYLKAAQELLDEVVSVGKVERQEVQMKKAKLVAMLDEVDQRYRQYHHQMHLVVSSFEAAAG
LGAAKTYTALALQTISRQFRCLRDAISSQIKSASKALGEEDGSRLRYVDHQLRQQRALLGMINAWRPQR
GLPERSVSVLRAWLFEHFLHPYPKDADKHMLAKQTGLTRSQVSNWFINARVRLWKPMVEEMYMEEM
R
>evm_27.model.AmTr_v1.0_scaffold00059.154
-----------MELLEEFCSVGRADRFEHQRRKAKLLSMVDEVDRRYSHYCDQMQMVVNSFDSIVGIGAATPY
TVLAQKAMSRHFRCLRDAINGQLKLISELLGEKETPRLKLLEQSLRQQRAFLGMMEAWRPQRGLPERS
VNILRAWLFEHFLHPY-------------------VSNWFINARVRLWKPMVEEMYQEESK
>evm_27.model.AmTr_v1.0_scaffold00165.33
ILKSSTFLKPAQQLLDEFCNVGQSDRAENHKKKTKLVSMLNEVFQRYKLYYNQMQMVMTSFECVAGL
SAAAPYTALALRAMSKHFKCLKNAIADQLLSTSRALGEDCSTSLRILDQSLRHQRTVSAIIHVWRPQRGL
PERAVAVLRAWLFEHFLHPYPTDTDKQMLAKQTGLTRNQVSNWFINARVRLWKPMVEEMHMLENK
